# Dose rate effect on mortality from ischemic heart disease in the cohort of Russian Mayak Production Association workers

**DOI:** 10.1038/s41598-023-28954-w

**Published:** 2023-02-02

**Authors:** Tamara V. Azizova, Evgeniya S. Grigoryeva, Nobuyuki Hamada

**Affiliations:** 1 Clinical Department, Southern Urals Biophysics Institute, Ozyorsk, Chelyabinsk Region Russia; 2 Biology and Environmental Chemistry Division, Sustainable System Research Laboratory, Central Research Institute of Electric Power Industry (CRIEPI), Tokyo, Japan

**Keywords:** Diseases, Health care, Risk factors

## Abstract

For improvement of the radiation protection system it is crucial to know the factors that modify the radiation dose–response relationship. One of such key factors is the ionizing radiation dose rate. There are, however, very few studies that examine the impact of the dose rate on radiogenic risks observed in human cohorts exposed to radiation at various dose rates. Here we investigated the impact of the dose rate (in terms of the recorded annual dose) on ischemic heart disease (IHD) mortality among Russian nuclear workers chronically exposed to radiation. We observed significantly increased excess relative risks (ERR) of IHD mortality per unit of external gamma-ray absorbed dose accumulated at higher dose rates (0.005–0.050 Gy/year). The present findings provide evidence for the association between radiation dose rate and ERRs of IHD mortality in occupationally chronically exposed workers per unit total dose. IHD mortality risk estimates considerably increased with increasing duration of uninterrupted radiation exposure at high rates. The present findings are consistent with other studies and can contribute to the scientific basis for recommendations on the radiation protection system.

## Introduction

Estimates of the radiation-related health detriment on cancer have largely been based on the findings obtained from the Life-Span Study (LSS) of Japanese atomic bomb survivors in Hiroshima and Nagasaki^1–3^. Members of the LSS cohort acutely received ionizing radiation exposure within 10 s at high dose rates^4^. However, dose rates of concern in radiation protection system for public and occupational exposures that are protracted exposures at low dose rates are markedly lower than those experienced in the LSS cohort.

The question remains open about the impact of dose rate on radiation health risk for outcomes (i.e., cancer and non-cancer incidence and mortality) in various exposure scenarios. Epidemiological studies focusing on this important issue are sparse^5–8^. For instance, the study of leukemia mortality risks following chronic external radiation exposure in the French cohort of nuclear workers demonstrated the association of the radiogenic risk of leukemia except for chronic lymphoid leukemia (non-CLL leukemia) with an annual radiation dose rate, such that excess relative risk per unit equivalent dose (ERR/Sv) for non-CLL leukemia were − 8.02 at < 10 mSv/year, 3.44 at 10–20 mSv/year and 16.66 at > 20 mSv/year^5^. In the mortality studies of UK Hanford nuclear workers, the ERR/Sv estimates differ significantly with dose rates for diseases of the circulatory system (DCS), but not for all cancers (excluding leukemia) and non-CLL leukemia^6,7^. That is why studies on risks of cancer and non-cancer outcomes in various cohorts following high- and low-dose rate radiation exposures are so important for preparing the next set of recommendations for radiation protection by the International Commission on Radiological Protection (ICRP)^9,10^.

The present study aims to investigate the impact of radiation dose rates on mortality from ischemic heart disease (IHD) in the Russian cohort of nuclear workers in the Mayak Production Association (PA) chronically exposed to ionizing radiation.

## Materials and methods

### Ethics declarations

The study was performed in line with the principles of the Declaration of Helsinki.

### Informed consent statement

This was a retrospective record-based epidemiological study that did not involve human participants and was based on depersonalized data stored in the database maintained by the Southern Urals Biophysics Institute (SUBI). The need for informed consent was waived due to retrospective nature of the study. The study was reviewed and approved by the Ethics Review Board of SUBI.

### The study cohort and the follow-up

This retrospective record-based study considered depersonalized data on the cohort of workers at the Russian nuclear production facility Mayak PA that had started its operation in the Southern Urals close to the city of Ozyorsk in 1948. Mayak PA included main facilities (reactors, radiochemical and plutonium production plant) and auxiliary facilities (e.g., mechanical repair plant, water treatment facility, electric power network department)^11^. The cohort comprised all workers that had been hired at main facilities of Mayak PA in 1948–1982 regardless of their sex, age, ethnicity, education level, social status or other characteristics.

The cohort follow-up began on a date of hire at one of the main facilities and ended at the earliest of the following dates: date of death; 31 December 2018 for those workers who were known to be alive and residing in Ozyorsk (residents); 31 December 2005 for those workers who were known to be alive but who had left Ozyorsk for another place of residence (migrants); date of the latest registered medical information for those workers whose vital status was unknown. Inconsistent end dates of the follow-up between residents and migrants are due to the fact that after 31 December 2005 when the personal data protection legislation had been implemented in the Russian Federation it became impossible to obtain any information about migrants.

It should be noted that all Mayak PA workers were living in Ozyorsk while working at the facility. Workers who had been living in Ozyorsk throughout the whole follow-up period (until death or 31 December 2018) are referred to as ‘residents’. Workers who had left the Mayak PA and the city for another place of residence are referred to as ‘migrants’.

A criterion for assigning a migrant status to a worker was a date of leaving Ozyorsk city for another permanent place of residence registered by a special state service. It should be noted that Ozyorsk has been an administrative territorial unit with restricted access since it was founded.

43 workers who suffered from acute radiation sickness following acute gamma-neutron high dose-rate exposure were excluded from the dataset for the analysis.

Mortality from IHD as a main cause of death (coded in accordance with the 9th revision of the International Classification of Diseases (ICD-9) as 410–414^12^) was considered as an outcome in this study.

The only available source of information on causes of death for migrants was medical death certificates, while for residents additional sources were available (autopsy and forensic reports, hospital charts and records). They allowed to verify a cause of death^13^. These diversities were one of the reasons why IHD incidence was analyzed for the resident subcohort separately. In the main text of the paper, we present the results of the analysis of the IHD mortality association with radiation dose rate only in the resident subcohort. This is due to the following reasons:

- the follow-up period was complete for the resident subcohort, which started on a date of hire and continued until a date of death with annual routine health check-ups;
- the quality of clinical verification for causes of death in residents was higher due to a large number of autopsy examinations performed for members of the resident subcohort (52.0%) than the migrant subcohort (12.0%);
- the number of workers who had potentially received internal radiation exposure and for whom bioassay alpha activity measured was higher in the resident subcohort (72.2%) than the migrant subcohort (6.0%);
- the number of workers for whom multiple bioassay measurements of alpha-activity were above the detection limit was higher in the resident subcohort (mean 6.32, standard deviations 6.41) than in the migrant subcohort (mean 3.16, standard deviations 5.27); and
- as a consequence, more data on organ and tissue absorbed alpha doses with lower uncertainties were available for the resident subcohort than for the migrant subcohort.

Results of the analysis for the entire cohort of Mayak PA workers are summarized in Supplementary Information (Tables S1–13, Figure S1).

### Dosimetry

The dosimetry system for the Mayak PA worker cohort has been updated several times over recent decades and this study is based on the Mayak Worker Dosimetry System 2013 (MWDS-2013)^14,15^ that provides improved individual estimates of annual gamma-ray, neutron and alpha-particle doses from external and internal exposures.

The majority of Mayak PA workers (76.1% of the entire cohort and 76.2% of the resident subcohort) received combined (both external and internal) radiation exposures and the rest of the workers received only external exposures to gamma-rays and/or neutrons. As noted earlier^16^, some Mayak workers were internally exposed to radionuclides other than plutonium, but the contribution of plutonium to the alpha dose in the Mayak worker cohort was the largest (> 90%).

Consistent with the previous study^16^, the present analyses considered doses from external and internal exposures absorbed in liver because MWDS-2013 provides no dose estimates for the circulatory system organs such as the heart. However, it should be noted that the biokinetic model underlying MWDS-2013 consists of three main parts: a systemic model, a gastrointestinal tract model and a respiratory tract model. The systemic model describes plutonium metabolism within the liver and other organs excluding the respiratory and gastrointestinal tracts. All the organ dose estimates based on the systemic biokinetic model are highly correlated (with a Pearson’s correlation coefficient of 0.99).

### Statistical analysis

The analysis considered the datasets each for the entire cohort and for the resident subcohort. The data for the analyses were compiled as Table S14.

Doses considered in all analyses were lagged for 10 years. At the first stage both sexes were considered together except that while performing the baseline analysis the heterogeneity between sexes was checked. Then all the analyses considered males and females separately. In this study, a lag period refers to a period of time just before death when it is thought that exposure can have no further effect on its occurrence. To conduct analyses considering lagged doses from exyetnal gamma rays, person-years were included in the analyses, beginning from the start date of employment, with the first x years included in the zero gamma dose category when the radiation dose was lagged for x years. The estimates of excess relative risk per unit absorbed dose (ERR/Gy) were based on the Poisson regression and computed with the AMFIT module of the EPICURE software^17^. 95% confidence intervals (CI) and *p* values demonstrating the statistical significance were computed with AMFIT module using the likelihood techniques. All statistical significance criteria were two-sided. The differences were considered significant at *p* < 0.05.

First, similarly to the previous study^16^, the ERR/Gy estimates were obtained using the conventional linear model that did not consider the dose rate. Adjustments via stratification were made for the following non-radiation factors: sex, attained age (< 20, 20–25, …, 80–85, > 85), calendar period (1948–1950, 1951–1955, 1956–1960, …, 2011–2015, 2016–2018), smoking status (never smoker, ever smoker, unknown), alcohol consumption (seldom drinker, moderate drinker, heavy drinker, unknown) and migration status (when the entire cohort was considered in the analyses) and for alpha dose from internal exposure. The analysis with the adjustment for alpha dose did not exclude from the dataset those workers who had not been monitored for internal exposure to alpha particles, instead they were assigned to “unknown” dose category (all workers with unmeasured bioassay alpha activity). So, the Poisson regression model used was

$$\uplambda =\uplambda _{0} \left( {{\text{s}},{\text{aa}},{\text{ct}},{\text{smok}},{\text{alc}},{\text{mig}},{\text{d}}_{\upalpha } } \right) \cdot \left( {1 +\upbeta \cdot {\text{D}}_{\upgamma } } \right),$$

where λ denotes the IHD mortality in the study cohort; λ_0_ denotes the background IHD mortality assuming the zero radiation dose; s denotes sex; aa denotes attained age; ct denotes calendar period; smok denotes smoking status; alc denotes alcohol consumption; mig denotes migration status (in the analysis considering the entire cohort), d_α_ denotes a categorical variable for the cumulative liver absorbed alpha dose from internal exposure (Gy); β denotes ERR/Gy; and D_γ_ denotes the cumulative liver absorbed gamma-ray dose from external exposure (Gy).

Then the analysis considering the dose rate based on annual doses recorded with individual film badges (as a sum of individual daily doses measured with a film badge dosimeter) was carried out using the following model:

$$\uplambda =\uplambda _{0} \left( {{\text{s}},{\text{aa}},{\text{ct}},{\text{smok}},{\text{alc}},{\text{mig}},{\text{d}}_{\upalpha } } \right) \cdot \left( {1 +\upbeta _{{\mathrm{L}}} \cdot {\text{D}}_{{\upgamma {\text{L}}}} +\upbeta _{{\text{H}}} \cdot {\text{D}}_{{\upgamma {\text{H}}}} } \right),$$

where D_γL_ denotes the total dose accumulated at a dose rate lower than a dose rate cutpoint, and D_γH_ denotes the total dose accumulated at a dose rate higher than a cutpoint (illustration of two dose-rate windows shown in Table 1)^18^, β_L_ and β_H_ denote the ERR/Gy estimates based on D_γL_ (ERR_L_/Gy) and D_γH_ (ERR_H_/Gy), respectively. The dose rate cutpoints were examined from 0.005 to 0.050 Gy/year with a 0.005 Gy/year interval. The comparison was made between the conventional model and the model considering the dose rate, using maximum likelihood techniques.

**Table 1:** Illustration of two dose-rate windows (e.g., at a cutpoint of 0.005 Gy/year)^18^.

Dose, Gy	Year							
	1971	1972	1973	1974	1975	1976	1977	1978
Annual dose	0.001	0.003	0.006	0.010	0.008	0.006	0.004	0.002
Annual dose < 0.005 Gy	0.001	0.003					0.004	0.002
Annual dose ≥ 0.005 Gy			0.006	0.010	0.008	0.006		
Cumulative dose	0.001	0.004	0.010	0.020	0.028	0.034	0.038	0.040
D_γL_—Cumulative dose received at annual dose rate < 0.005 Gy/year	0.001	0.004	0.004	0.004	0.004	0.004	0.008	0.010
D_γH_—Cumulative dose received at annual dose rate ≥ 0.005 Gy/year	0.000	0.000	0.006	0.016	0.024	0.030	0.030	0.030

Deviations from the conventional (linear) model of the dose–response were tested by fitting the dataset using alternative (linear-quadratic) models:

$$\begin{aligned}\uplambda & =\uplambda _{0} \left( {{\text{s}},{\text{aa}},{\text{ct}},{\text{smok}},{\text{alc}},{\text{mig}},{\text{d}}_{\upalpha } } \right) \cdot \left( {1 +\upbeta _{{{\text{L1}}}} \cdot {\text{D}}_{{\upgamma {\text{L}}}} +\upbeta _{{{\text{L2}}}} \cdot {\text{D}}_{{\upgamma {\text{L}}}}^{2} +\upbeta _{{{\text{H1}}}} \cdot {\text{D}}_{{\upgamma {\text{H}}}} } \right), \\\uplambda & =\uplambda _{0} \left( {{\text{s}},{\text{aa}},{\text{ct}},{\text{smok}},{\text{alc}},{\text{mig}},{\text{d}}_{\upalpha } } \right) \cdot \left( {1 +\upbeta _{{{\text{L1}}}} \cdot {\text{D}}_{{\upgamma {\text{L}}}} +\upbeta _{{{\text{H1}}}} \cdot {\text{D}}_{{\upgamma {\text{H}}}} +\upbeta _{{{\text{H2}}}} \cdot {\text{D}}_{{\upgamma {\text{H}}}}^{2} } \right),\;{\text{and}} \\\uplambda & =\uplambda _{0} \left( {{\text{s}},{\text{aa}},{\text{ct}},{\text{smok}},{\text{alc}},{\text{mig}},{\text{d}}_{\upalpha } } \right) \cdot \left( {1 +\upbeta _{{{\text{L1}}}} \cdot {\text{D}}_{{\upgamma {\text{L}}}} +\upbeta _{{{\text{L2}}}} \cdot {\text{D}}_{{\upgamma {\text{L}}}}^{2} +\upbeta _{{{\text{H1}}}} \cdot {\text{D}}_{{\upgamma {\text{H}}}} +\upbeta _{{{\text{H2}}}} \cdot {\text{D}}_{{\upgamma {\text{H}}}}^{2} } \right). \\ \end{aligned}$$

The comparison between the linear and the linear-quadratic models was based on the difference between the corresponding maximum likelihoods.

While assessing the ERR/Gy, the following sensitivity analyses were carried out:

- the effect of uninterrupted high dose rate exposure during 5 years was assessed;
- various lag periods (0, 5, 20 and 30 years) for external and internal occupational radiation doses were examined;
- an adjustment (via stratification) for internal alpha dose was excluded;
- an alternative adjustment for internal alpha dose was made using the approach when those workers who had not been monitored for internal alpha exposure were considered in two categories: one category included reactor workers exposed only externally, the other category included all the rest workers with unmeasured alpha activity;
- the linear trend with the weighted cumulative gamma-neutron dose (with a radiation weighting factor of 10 for the absorbed neutron dose^19^) was analyzed. The radiation weighting factor for neutrons was chosen in accordance with ICRP Publication 103 taking into account the energy of neutron spectrum in the Mayak PA workplace^19,20^. To assess the weighted cumulative gamma-neutron dose the unmeasured neutron dose was given 0.00 value;
- adjustments (via stratification) for additional factors were included: period of hire (1948–1958, 1959–1972, 1973–1982), age at hire (< 20, 20–30, ≥ 30);
- the dataset considered in the analysis was limited to workers who had been employed for > 1 year.

## Results

At the end of the follow-up period, 3824 deaths from DCS were registered as the main cause of death in the resident subcohort over 622,199 person-years of the follow-up, among which there were 2267 (59.3%) deaths from IHD. Tables S15 and S16 summarize distributions of person-years and numbers of deaths from IHD within various gamma dose categories by dose rates.

Tables 2 and 3 summarize main characteristics of the Mayak worker cohort and the resident subcohort.

**Table 2:** Main characteristics of the Mayak PA worker cohort.

Characteristics	Entire cohort			Resident subcohort		
	Both	Males	Females	Both	Males	Females
Number of workers comprising the cohort	22,377	74.6%^a^	25.4%^a^	13,156	72.1%^b^	27.9%^b^
Number of workers excluding those diagnosed with acute radiation sickness	22,334	74.6%^c^	25.4%^c^	13,124	72.1%^d^	27.9%^d^
Number of workers with known vital status at the end of the follow-up^e^	95.4%	95.2%	95.6%	99.9%	100%	99.9%
Number of workers who died^f^	67.2%	69.3%	61.4%	69.3%	71.0%	64.9%
Number of those who died for whom a cause of death was available^g^	99.8%	99.7%	99.8%	99.8%	99.8%	99.8%
Means and standard deviations (SDs) of age at hire, years	24.9 (7.5)	24.1 (7.13)	27.3 (8.0)	25.5 (7.9)	24.4 (7.4)	28.4 (8.3)
Means and SDs of duration of employment, years	18.1 (14.3)	18.4 (14.8)	17.4 (12.8)	26.1 (12.9)	27.2 (13.2)	23.2 (11.7)
Means and SDs of age at death for those workers who died, years	64.6 (14,1)	62.3 (13.8)	72.1 (12.6)	65.4 (14.5)	62.8 (14.1)	72.6 (13.0)

^a^From number of workers comprising the cohort (both sex).

^b^From number of workers comprising the resident subcohort (both sex).

^c^From number of workers comprising the cohort excluding those diagnosed with acute radiation sickness (both sex).

^d^From number of workers comprising the resident subcohort excluding those diagnosed with acute radiation sickness (both sex).

^e^From number of workers comprising the cohort.

^f^From number of workers with known vital status at the end of the follow-up.

^g^From number of workers who died.

**Table 3:** Distribution of the Mayak PA workers by various parameters.

Parameter	Entire cohort			Resident subcohort		
	Both	Males	Females	Both	Males	Females
Calendar period of hire, years						
1948–1953	8406	5485	2921	3693	2336	1357
1954–1958	3891	3233	658	2010	1572	438
1959–1963	3837	3221	616	2149	1662	487
1964–1972	2769	2197	572	2202	1682	520
1973–1978	2371	1770	601	2087	1523	564
1979–1982	1103	782	321	1015	709	306
Age at hire, years old						
≤ 20	7804	6642	1162	4431	3744	687
21–30	10,348	7491	2857	5792	4113	1679
31–40	3002	1795	1207	2093	1157	936
> 40	1223	760	463	840	470	370
Duration of employment, years						
< 1	1057	840	217	201	133	68
1–10	8174	6160	2014	1706	1152	554
> 10	13,146	9688	3458	11,249	8199	3050
Age at death of those who died, years						
≤ 20	16	15	1	15	14	1
21–30	318	294	24	216	197	19
31–40	572	520	52	332	290	42
41–50	1255	1137	118	734	656	78
51–60	2926	2536	390	1765	1501	264
61–70	3995	3314	681	2410	1943	467
71–80	3567	2393	1174	2327	1543	784
81–90	1546	734	812	1182	537	645
> 90	167	74	93	131	49	82
Age at the end of the follow-up of those who were alive, years						
≤ 60	1150	1050	100	691	625	66
61–70	2593	2153	440	1383	1073	310
71–80	2256	1282	974	1175	742	433
81–90	874	353	521	697	285	412
> 90	102	32	70	97	29	68

**Figure 1:**
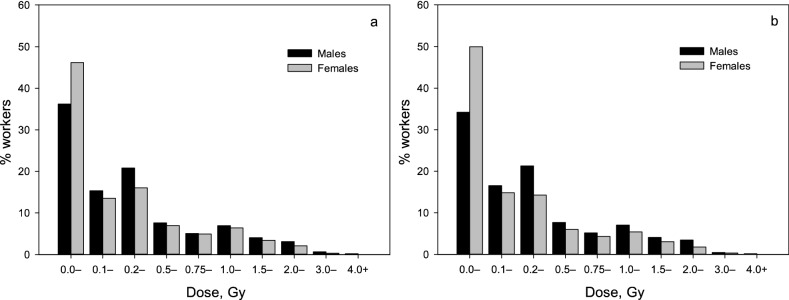
Distribution of workers in the entire cohort (**a**) and the resident subcohort (**b**) by cumulative liver absorbed gamma-ray dose from external exposure.

At the end of follow-up the means (standard deviations) of cumulative liver absorbed gamma-ray doses from external exposure were 0.43 (0.63) Gy for both sexes, 0.45 (0.65) Gy for males and 0.37 (0.56) Gy for females in the entire cohort, and 0.42 (0.60) Gy for both sexes, 0.45 (0.63) Gy for males and 0.33 (0.53) Gy for females in the resident subcohort. Figure 1 demonstrates the distribution of the entire cohort workers and resident subcohort workers by external gamma-ray dose. The distributions of workers by the cumulative liver absorbed gamma-ray dose from external exposure did not significantly differ between the entire cohort and the resident subcohort (*p* = 0.09).

**Figure 2:**
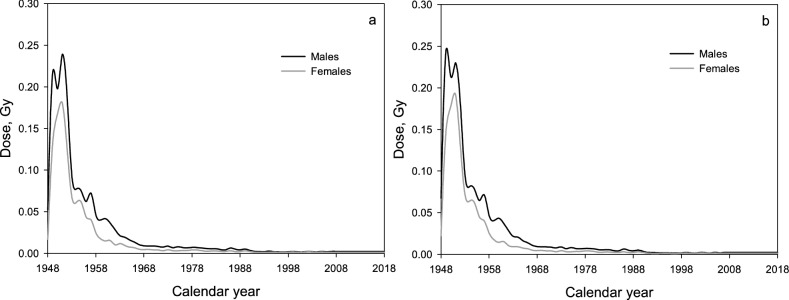
Changes in the mean annual liver absorbed gamma-ray dose from external exposure in the entire cohort (**a**) and the resident subcohort (**b**) of Mayak PA workers in relation to a calendar year.

Means (standard deviations) of annual liver absorbed gamma-ray doses from chronic external exposure were 0.053 (0.110) Gy for both sexes, 0.054 (0.114) Gy for males and 0.050 (0.096) Gy for females in the entire cohort, and 0.030 (0.076) Gy for both sexes, 0.030 (0.078) Gy for males and 0.030 (0.068) Gy for females in the resident subcohort. The changes in mean annual gamma-ray doses over the whole employment period are demonstrated in Fig. 2. It should be noted that in early years of Mayak PA operation mean annual gamma-ray doses from external exposure were the highest. In 1951 the mean dose rate was 0.25 Gy/year; during the following decade the dose sharply declined down to 0.05 Gy/year by 1960. The annual doses continued leveling down gradually in the 1960s–1980s and thereafter remained stable at approximately 0.008 Gy/year.

It should also be noted that 4083 (18.2%) workers of the entire cohort and 2583 (19.6%) workers of the resident subcohort who had been working at reactors and at some departments of the radiochemical and the plutonium production plants had been exposed to neutrons. Means (standard deviations) of cumulative liver absorbed neutron doses were 0.0011 (0.0042) Gy for both sexes, 0.0011 (0.0044) Gy for males and 0.0013 (0.0048) Gy for females in the entire cohort, and 0.0012 (0.0045) Gy for both sexes, 0.0012 (0.0044) Gy for males and 0.0014 (0.0052) Gy for females in the resident subcohort. Neutron dose distributions in the entire cohort and resident subcohort of Mayak workers are summarized in Table S14.

In accordance with MWDS-2013, bioassay alpha activity due to incorporation of plutonium (24-h urine) was measured only in 44.8% (42.0% of males/52.6% of females) of the entire cohort workers and in 72.2% (70.3% of males/76.9% of females) of the resident subcohort workers who had been exposed to combined radiation. Means (standard deviations) of cumulative liver absorbed alpha doses from internal exposure to incorporated plutonium were 0.25 (1.19) Gy for both sexes, 0.18 (0.65) Gy for males and 0.40 (1.92) Gy for females in the entire cohort, and 0.22 (1.12) Gy for both sexes, 0.17 (0.62) Gy for males and 0.34 (1.79) Gy for females in the resident subcohort. Distributions of internal alpha dose in the entire cohort and the resident subcohort by alpha dose from internal exposure are shown in Table S17.

In the previous studies of DCS mortality, including IHD mortality^16^, while using a conventional linear model that included adjustments for non-radiation factors (sex, attained age, calendar period, smoking status and alcohol consumption status, migration status for the analysis considering the entire Mayak worker cohort) and for alpha dose from internal exposure, there was no significant association of IHD mortality with the cumulative liver absorbed gamma-ray dose from external exposure: the ERR/Gy was 0.06 (95% CI − 0.04; 0.18) in males and 0.14 (95% CI − 0.07; 0.45) in females.

### The baseline analysis

**Figure 3:**
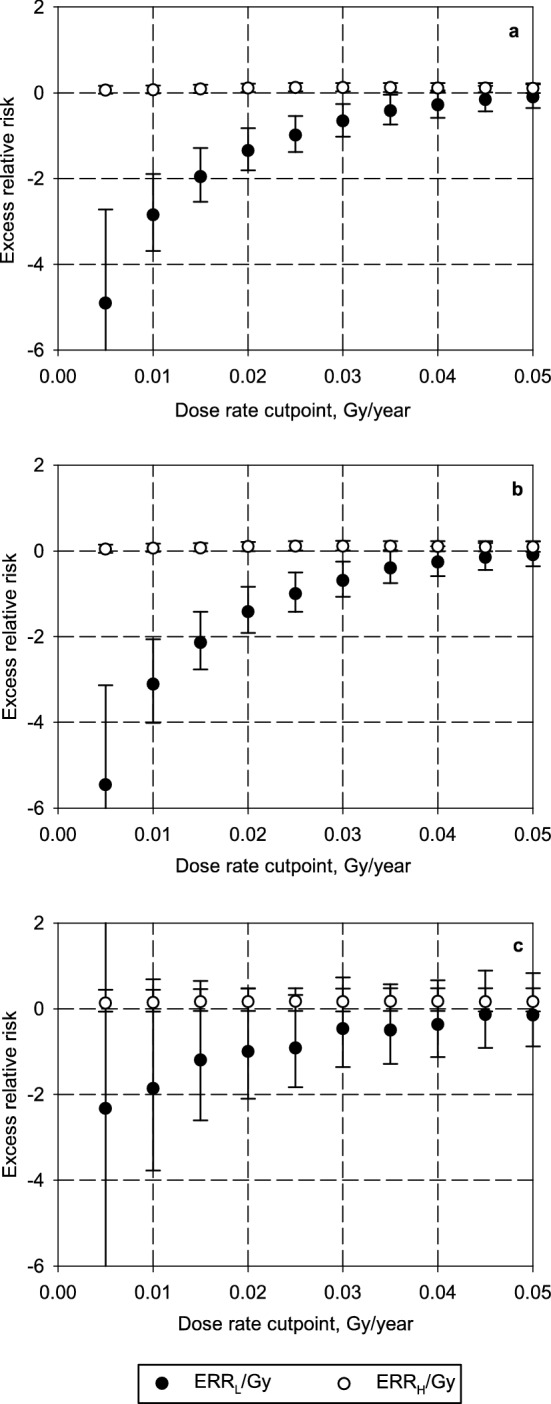
Excess relative risk per Gy of IHD mortality in the resident subcohort in relation to 10-year lagged cumulative liver absorbed doses from external gamma-ray exposure, adjusted for various non-radiation factors and alpha absorbed dose to the liver (main analysis, residents): a—both sexes, b—males, c—females.

In this study the ERR/Gy of gamma-ray dose for IHD mortality was assessed with another model that took into account the dose rate. The results of the baseline analysis are presented in Table 4 and Fig. 3.

**Table 4:** Excess relative risk per Gy of IHD mortality in relation to 10-year lagged cumulative liver absorbed doses from external radiation exposure, adjusted for various non-radiation factors and alpha absorbed dose to the liver (main analysis, residents).

Cutpoint, Gy/year	Model parameters	Both sexes	Males	Females	*p* value^a^
0 (without cutpoint^16^)	ERR/Gy	0.07 (− 0.02; 0.18)	0.06 (− 0.04; 0.18)	0.14 (− 0.07; 0.45)	> 0.50
0.005	ERR_L_/Gy	**− 4.91 (− 6.78, − 2.72)**	**− 5.46 (− 7.41, − 3.13)**	− 2.33 (− 6.98, 4.01)	0.425
	ERR_H_/Gy	0.06 (− 0.02, 0.16)	0.04 (− 0.04, 0.15)	0.13 (− 0.07, 0.44)	
	*p* value^b^	**< 0.001**	**< 0.001**	0.402	
0.010	ERR_L_/Gy	**− 2.85 (− 3.69, − 1.89)**	**− 3.11 (− 4.01, − 2.06)**	− 1.86 (− 3.77, 0.69)	0.457
	ERR_H_/Gy	0.07 (− 0.01, 0.17)	0.06 (− 0.02, 0.17)	0.14 (− 0.07, 0.44)	
	*p* value^b^	**< 0.001**	**< 0.001**	0.112	
0.015	ERR_L_/Gy	**− 1.96 (− 2.54, − 1.29)**	**− 2.14 (− 2.76, − 1.42)**	− 1.20 (− 2.60, 0.65)	0.417
	ERR_H_/Gy	**0.09 (+ 0.00, 0.19)**	0.07 (− 0.01, 0.18)	0.16 (− 0.05, 0.46)	
	*p* value^b^	**< 0.001**	**< 0.001**	0.135	
0.020	ERR_L_/Gy	**− 1.35 (− 1.81, − 0.82)**	**− 1.42 (− 1.91, − 0.84)**	− 1.00 (− 2.10, 0.48)	> 0.50
	ERR_H_/Gy	**0.11 (0.02, 0.21)**	**0.10 (+ 0.00, 0.21)**	0.16 (− 0.05, 0.47)	
	*p* value^b^	**< 0.001**	**< 0.001**	0.111	
0.025	ERR_L_/Gy	**− 0.99 (− 1.38, − 0.54)**	**− 1.00 (− 1.42, − 0.50)**	− 0.92 (− 1.83, 0.32)	> 0.50
	ERR_H_/Gy	**0.12 (0.03, 0.23)**	**0.11 (0.01, 0.23)**	0.17 (− 0.05, 0.48)	
	*p* value^b^	**< 0.001**	**< 0.001**	0.079	
0.030	ERR_L_/Gy	**− 0.66 (− 1.02, − 0.26)**	**− 0.69 (− 1.07, − 0.25)**	− 0.47 (− 1.36, 0.73)	> 0.50
	ERR_H_/Gy	**0.12 (0.03, 0.23)**	**0.11 (0.01, 0.24)**	0.16 (− 0.06, 0.47)	
	*p* value^b^	**< 0.001**	**0.001**	0.268	
0.035	ERR_L_/Gy	**− 0.42 (− 0.74, − 0.04)**	− 0.40 (− 0.75, 0.02)	− 0.50 (− 1.29, 0.57)	> 0.50
	ERR_H_/Gy	**0.12 (0.02, 0.23)**	**0.11 (+ 0.00, 0.23)**	0.17 (− 0.05, 0.48)	
	*p* value^b^	**0.009**	**0.026**	0.196	
0.040	ERR_L_/Gy	− 0.28 (− 0.58, 0.06)	− 0.26 (− 0.58, 0.11)	− 0.37 (− 1.13, 0.66)	> 0.50
	ERR_H_/Gy	**0.11 (0.02, 0.23)**	0.10 (− 0.01, 0.23)	0.17 (− 0.05, 0.48)	
	*p* value^b^	**0.033**	0.072	0.278	
0.045	ERR_L_/Gy	− 0.16 (− 0.43, 0.16)	− 0.15 (− 0.44, 0.19)	− 0.14 (− 0.91, 0.89)	> 0.50
	ERR_H_/Gy	**0.11 (0.01, 0.23)**	0.09 (− 0.01, 0.23)	0.16 (− 0.06, 0.48)	
	*p* value^b^	0.12	0.175	> 0.50	
0.050	ERR_L_/Gy	− 0.10 (− 0.36, 0.20)	− 0.09 (− 0.36, 0.23)	− 0.15 (− 0.88, 0.83)	> 0.50
	ERR_H_/Gy	**0.10 (+ 0.00, 0.22)**	0.09 (− 0.02, 0.22)	0.16 (− 0.06, 0.48)	
	*p* value^b^	0.209	0.31	0.498	

Numbers in bold indicate significant differences. The dataset for the analysis was stratified by sex, attained age, calendar period, smoking status, alcohol consumption, alpha dose.

*ERR/Gy* excess relative risk per unit gray of gamma-ray dose, *IHD* ischemic heart disease (ICD-9 codes: 410–414).

^a^Test for heterogeneity between sexes.

^b^Likelihood ratio test comparing the models with and without cutpoint.

IHD mortality risks estimated with the conventional model (without considering dose rate cutpoints) were significantly different from the corresponding risks estimated with the model used in this study (considering dose rate cutpoints) for both sexes (except for cutpoints of 0.045 and 0.050 Gy/year) and for males (except for cutpoints of 0.040, 0.045 and 0.050 Gy/year) in the resident subcohort. Females of the resident subcohort showed no significant differences in the risk estimates between the models (Table 4).

It should be noted that for all dose rate cutpoints, the estimates of ERR_H_/Gy (due to higher dose rates) were higher than ERR_L_/Gy (due to lower dose rates) for both sexes, males and females in the resident subcohort.

IHD mortality risks in the resident subcohort significantly increased at dose rates of > 0.015, > 0.020, > 0.025, > 0.030, > 0.035, > 0.040, > 0.045, and > 0.050 Gy/year when compared to exposures at dose rates below the specified cutpoints. There was no significantly increased risk in females of the resident subcohort, but there was no significant difference between sexes at any dose rate cutpoint (Table 4).

### Sensitivity analyses

We performed a sensitivity analysis to assess the effect of uninterrupted duration of high dose-rate exposure over 5 years on the risk estimate. Table 5 summarizes the results of this analysis. The analysis demonstrated that high dose rate exposure during 5 years notably increased the IHD mortality risk (ERR_H5_/Gy, Table 5) compared to the risk estimate due to high dose rate exposure during 1 year (ERR_H_/Gy, Table 4).

**Table 5:** Excess relative risk of IHD mortality per Gy in relation to 10-year lagged cumulative liver absorbed doses from external radiation exposure, adjusted for various non-radiation factors and alpha absorbed dose to the liver (sensitivity analysis, residents).

Cutpoint, Gy/year	Model parameters	Both sexes	Males	Females	*p* value^a^
0 (without cutpoint^16^)	ERR/Gy	0.07 (− 0.02; 0.18)	0.06 (− 0.04; 0.18)	0.14 (− 0.07; 0.45)	> 0.50
0.005	ERR_L5_/Gy	− 0.03 (− 0.14, 0.11)	− 0.07 (− 0.17, 0.08)	0.12 (− 0.14, 0.51)	0.487
	ERR_H5_/Gy	**0.27 (0.07, 0.50)**	**0.28 (0.07, 0.52)**	0.24 (− 0.48, 1.14)	
	*p* value^b^	**0.030**	**0.017**	> 0.50	
0.010	ERR_L5_/Gy	− 0.06 (− 0.16, 0.07)	− 0.09 (− 0.19, 0.04)	0.10 (− 0.15, 0.47)	0.419
	ERR_H5_/Gy	**0.36 (0.14, 0.61)**	**0.36 (0.14, 0.62)**	0.33 (− 0.47, 1.36)	
	*p* value^b^	**0.003**	**0.002**	> 0.50	
0.015	ERR_L5_/Gy	− 0.06 (− 0.15, 0.06)	− 0.09 (− 0.18, 0.04)	0.05 (− 0.18, 0.41)	0.482
	ERR_H5_/Gy	**0.39 (0.16, 0.62)**	**0.38 (0.15, 0.64)**	0.58 (− 0.31, 1.71)	
	*p* value^b^	**0.002**	**0.002**	0.340	
0.020	ERR_L5_/Gy	− 0.07 (− 0.16, 0.05)	− 0.09 (− 0.18, 0.03)	0.03 (− 0.19, 0.36)	0.444
	ERR_H5_/Gy	**0.46 (0.21, 0.75)**	**0.43 (0.19, 0.72)**	0.81 (− 0.17, 2.08)	
	*p* value^b^	**< 0.001**	**< 0.001**	0.181	
0.025	ERR_L5_/Gy	− 0.05 (− 0.14, 0.07)	− 0.07 (− 0.17, 0.06)	0.02 (− 0.20, 0.35)	0.464
	ERR_H5_/Gy	**0.45 (0.18, 0.75)**	**0.42 (0.15, 0.72)**	0.91 (− 0.12, 2.25)	
	*p* value^b^	**0.002**	**0.004**	0.142	
0.030	ERR_L5_/Gy	− 0.04 (− 0.13, 0.08)	− 0.06 (− 0.16, 0.07)	0.04 (− 0.18, 0.38)	> 0.50
	ERR_H5_/Gy	**0.48 (0.19, 0.81)**	**0.45 (0.16, 0.79)**	0.82 (− 0.25, 2.22)	
	*p* value^b^	**0.003**	**0.004**	0.218	
0.035	ERR_L5_/Gy	− 0.01 (− 0.11, 0.12)	− 0.02 (− 0.13, 0.11)	0.05 (− 0.17, 0.38)	> 0.50
	ERR_H5_/Gy	**0.39 (0.09, 0.74)**	**0.36 (0.06, 0.72)**	0.82 (− 0.30, 2.31)	
	*p* value^b^	**0.028**	**0.040**	0.243	
0.040	ERR_L5_/Gy	− 0.01 (− 0.10, 0.11)	− 0.03 (− 0.13, 0.10)	0.08 (− 0.14, 0.42)	> 0.50
	ERR_H5_/Gy	**0.45 (0.13, 0.82)**	**0.43 (0.11, 0.82)**	0.61 (− 0.53, 2.13)	
	*p* value^b^	**0.014**	**0.015**	0.437	
0.045	ERR_L5_/Gy	0.01 (− 0.08, 0.13)	− 0.01 (− 0.11, 0.12)	0.09 (− 0.13, 0.43)	> 0.50
	ERR_H5_/Gy	**0.37 (0.05, 0.76)**	**0.36 (0.03, 0.75)**	0.56 (− 0.62, 2.12)	
	*p* value^b^	0.055	0.058	> 0.50	
0.050	ERR_L5_/Gy	0.03 (− 0.07, 0.15)	0.01 (− 0.09, 0.14)	0.08 (− 0.14, 0.41)	> 0.50
	ERR_H5_/Gy	0.32 (− 0.00, 0.72)	0.29 (− 0.04, 0.69)	0.74 (− 0.49, 2.42)	
	*p* value^b^	0.125	0.161	0.361	

Numbers in bold indicate significant differences. The dataset for the analysis was stratified by sex, attained age, calendar period, smoking status, alcohol consumption, and alpha dose.

Assessment of the effect of the uninterrupted high dose rate exposure over 5 years.

*ERR/Gy* excess relative risk per unit gray of gamma-ray dose, *IHD* ischemic heart disease (ICD-9 codes: 410–414).

^a^Test for heterogeneity between sexes.

^b^Likelihood ratio test comparing the models with and without cutpoint.

Tables 6, S18 and S19 summarize IHD mortality risks analyzed for associations with the dose rate lagged for various periods (0, 5, 20, 30 years). First, it should be noted that differences in IHD mortality risks for the resident subcohort between the conventional model and the alternative model were significant at every cutpoint with any of the lag period (except at some certain cutpoints with some certain lag periods) (Table 6). The same tendency was observed separately for males of the resident subcohort (Table S18), and there were significant differences in females only at two cutpoints with a zero lag-period (0.020 and 0.025 Gy/year) (Table S19). However, the IHD mortality risk estimates at higher dose rates for both sexes (Table 6) and for males (Table S18) in the resident subcohort while being lagged for more than 10 years decreased down to the non-significance level with the increasing lag period.

**Table 6:** Excess relative risk per Gy of IHD mortality in relation to cumulative liver absorbed doses from external gamma-ray exposure, adjusted for various non-radiation factors and alpha absorbed dose to the liver (sensitivity analyses—various lag periods, both sexes, residents).

Cutpoint, Gy/year	Model parameters	Lag periods			
		0 years	5 years	20 years	30 years
0 (without cutpoint^16^)	ERR/Gy	0.09 (− 0.00, 0.19)^16^	0.08 (− 0.01, 0.18)	0.06 (− 0.03, 0.17)	0.06 (− 0.04, 0.18)
0.005	ERR_L_/Gy	**− 5.72 (− 7.13, − 4.04)**	**− 5.12 (− 6.78, − 3.18)**	**− 4.33 (− 7.06, − 1.09)**	− 5.20 (− 9.57, 0.13)
	ERR_H_/Gy	0.06 (− 0.02, 0.16)	0.06 (− 0.02, 0.16)	0.05 (− 0.04, 0.15)	0.05 (− 0.04, 0.16)
	*p* value^a^	**< 0.001**	0.152	**0.01**	0.053
0.010	ERR_L_/Gy	**− 3.18 (− 3.88, − 2.37)**	**− 2.96 (− 3.72, − 2.08)**	**− 2.79 (− 3.87, − 1.53)**	**− 2.89 (− 4.47, − 1.01)**
	ERR_H_/Gy	**0.09 (0.01, 0.19)**	**0.08 (+ 0.00, 0.18)**	0.06 (− 0.03, 0.16)	0.05 (− 0.04, 0.16)
	*p* value^a^	**< 0.001**	**0.05**	**< 0.001**	**0.003**
0.015	ERR_L_/Gy	**− 2.18 (− 2.68, − 1.60)**	**− 2.06 (− 2.59, − 1.44)**	**− 1.83 (− 2.56, − 0.97)**	**− 2.08 (− 3.05, − 0.92)**
	ERR_H_/Gy	**0.11 (0.02, 0.21)**	**0.10 (0.01, 0.19)**	0.07 (− 0.02, 0.17)	0.06 (− 0.03, 0.17)
	*p* value^a^	**< 0.001**	**0.023**	**< 0.001**	**< 0.001**
0.020	ERR_L_/Gy	**− 1.52 (− 1.92, − 1.05)**	**− 1.42 (− 1.85, − 0.93)**	**− 1.28 (− 1.83, − 0.63)**	**− 1.44 (− 2.17, − 0.58)**
	ERR_H_/Gy	**0.13 (0.04, 0.23)**	**0.11 (0.03, 0.22)**	0.08 (− 0.01, 0.19)	0.07 (− 0.03, 0.18)
	*p* value^a^	**< 0.001**	**0.009**	**< 0.001**	**0.001**
0.025	ERR_L_/Gy	**− 1.13 (− 1.47, − 0.73)**	**− 1.05 (− 1.42, − 0.63)**	**− 0.88 (− 1.35, − 0.33)**	**− 1.14 (− 1.72, − 0.45)**
	ERR_H_/Gy	**0.15 (0.05, 0.26)**	**0.13 (0.04, 0.24)**	0.09 (− 0.00, 0.20)	0.08 (− 0.02, 0.20)
	*p* value^a^	**< 0.001**	**0.005**	**0.001**	**0.001**
0.030	ERR_L_/Gy	**− 0.80 (− 1.12, − 0.44)**	**− 0.73 (− 1.06, − 0.35)**	**− 0.57 (− 0.99, − 0.09)**	**− 0.76 (− 1.27, − 0.15)**
	ERR_H_/Gy	**0.15 (0.05, 0.27)**	**0.13 (0.04, 0.24)**	0.09 (− 0.00, 0.21)	0.08 (− 0.02, 0.20)
	*p* value^a^	**< 0.001**	**0.005**	**0.01**	**0.009**
0.035	ERR_L_/Gy	**− 0.56 (− 0.85, − 0.23)**	**− 0.50 (− 0.81, − 0.15)**	− 0.33 (− 0.72, 0.11)	− 0.50 (− 0.97, 0.05)
	ERR_H_/Gy	**0.15 (0.05, 0.27)**	**0.13 (0.03, 0.25)**	0.09 (− 0.01, 0.20)	0.08 (− 0.02, 0.21)
	*p* value^a^	**< 0.001**	**0.007**	0.072	**0.044**
0.040	ERR_L_/Gy	**− 0.41 (− 0.67, − 0.11)**	**− 0.36 (− 0.63, − 0.04)**	− 0.21 (− 0.56, 0.19)	− 0.36 (− 0.78, 0.14)
	ERR_H_/Gy	**0.15 (0.05, 0.27)**	**0.13 (0.03, 0.24)**	0.09 (− 0.01, 0.20)	0.08 (− 0.02, 0.21)
	*p* value^a^	**0.001**	**0.01**	0.153	0.087
0.045	ERR_L_/Gy	− 0.28 (− 0.52, 0.01)	− 0.23 (− 0.49, 0.06)	− 0.09 (− 0.41, 0.28)	− 0.18 (− 0.58, 0.28)
	ERR_H_/Gy	**0.14 (0.04, 0.27)**	**0.12 (0.02, 0.24)**	0.08 (− 0.02, 0.20)	0.08 (− 0.03, 0.20)
	*p* value^a^	**0.009**	**0.015**	0.377	0.268
0.050	ERR_L_/Gy	− 0.20 (− 0.43, 0.07)	− 0.16 (− 0.40, 0.12)	− 0.02 (− 0.32, 0.33)	− 0.11 (− 0.48, 0.33)
	ERR_H_/Gy	**0.14 (0.03, 0.26)**	**0.12 (0.02, 0.24)**	0.07 (− 0.03, 0.19)	0.07 (− 0.03, 0.20)
	*p* value^a^	**0.03**	0.074	> 0.50	0.405

Numbers in bold indicate significant differences. The dataset for the analysis was stratified by sex, attained age, calendar period, smoking status, alcohol consumption, alpha dose.

*ERR/Gy* excess relative risk per unit gray of gamma-ray dose, *IHD* ischemic heart disease (ICD-9 codes: 410–414).

^a^Likelihood ratio test comparing the models with and without cutpoint.

The sensitivity analysis of the IHD mortality that considered the weighted cumulative gamma-ray and neutron liver absorbed dose (weighting factor of 10) provided similar results for both sexes (Table 7), males (Table S20) and females (Table S21) in the resident subcohort.

**Table 7:** Excess relative risk per Gy of IHD mortality in relation to 10-year lagged cumulative liver absorbed gamma-ray doses from external exposure (sensitivity analyses—various parameters of the adjustment for alpha and neutron dose, both sexes, residents).

Cutpoint, Gy/year	Model parameters	Sensitivity analysis type		
		Exclusion of the adjustment for liver absorbed alpha dose	The alternative adjustment for liver absorbed alpha dose^a^	Association with the weighted sum of liver absorbed gamma-ray + neutron dose (Gy)^b^
0 (without cutpoint^16^)	ERR/Gy	0.06 (− 0.01, 0.15)	**0.11 (0.01, 0.22)**	0.08 (− 0.01, 0.18)
0.005	ERR_L_/Gy	**− 4.77 (− 6.57, − 2.68)**	**− 5.30 (− 7.12, − 3.15)**	**− 4.80 (− 6.69, − 2.58)**
	ERR_H_/Gy	0.05 (− 0.02, 0.13)	0.08 (− 0.00, 0.19)	0.06 (− 0.02, 0.16)
	*p* value^c^	**< 0.001**	**< 0.001**	**< 0.001**
0.010	ERR_L_/Gy	**− 2.69 (− 3.51, − 1.74)**	**− 2.92 (− 3.77, − 1.94)**	**− 2.81 (− 3.65, − 1.85)**
	ERR_H_/Gy	0.06 (− 0.01, 0.14)	**0.10 (0.01, 0.21)**	0.07 (− 0.01, 0.17)
	*p* value^c^	**< 0.001**	**< 0.001**	**< 0.001**
0.015	ERR_L_/Gy	**− 1.79 (− 2.38, − 1.13)**	**− 1.88 (− 2.49, − 1.18)**	**− 1.96 (− 2.54, − 1.29)**
	ERR_H_/Gy	**0.07 (+ 0.00, 0.15)**	**0.11 (0.02, 0.22)**	**0.09 (+ 0.00, 0.19)**
	*p* value^c^	**< 0.001**	**< 0.001**	**< 0.001**
0.020	ERR_L_/Gy	**− 1.17 (− 1.64, − 0.64)**	**− 1.20 (− 1.69, − 0.62)**	**− 1.33 (− 1.79, − 0.80)**
	ERR_H_/Gy	**0.08 (0.01, 0.16)**	**0.13 (0.03, 0.24)**	**0.10 (0.02, 0.21)**
	*p* value^c^	**< 0.001**	**< 0.001**	**< 0.001**
0.025	ERR_L_/Gy	**− 0.81 (− 1.21, − 0.36)**	**− 0.82 (− 1.25, − 0.32)**	**− 0.96 (− 1.36, − 0.51)**
	ERR_H_/Gy	**0.08 (0.01, 0.17)**	**0.14 (0.04, 0.26)**	**0.11 (0.02, 0.22)**
	*p* value^c^	**< 0.001**	**< 0.001**	**< 0.001**
0.030	ERR_L_/Gy	**− 0.52 (− 0.87, − 0.11)**	**− 0.47 (− 0.87, − 0.01)**	**− 0.68 (− 1.03, − 0.28)**
	ERR_H_/Gy	**0.08 (0.01, 0.17)**	**0.14 (0.04, 0.26)**	**0.12 (0.03, 0.23)**
	*p* value^c^	**0.005**	**0.013**	**< 0.001**
0.035	ERR_L_/Gy	− 0.29 (− 0.60, 0.08)	− 0.22 (− 0.59, 0.22)	**− 0.44 (− 0.76, − 0.06)**
	ERR_H_/Gy	**0.08 (+ 0.00, 0.17)**	**0.13 (0.03, 0.25)**	**0.12 (0.02, 0.23)**
	*p* value^c^	0.056	0.123	**0.007**
0.040	ERR_L_/Gy	− 0.17 (− 0.45, 0.17)	− 0.08 (− 0.42, 0.32)	− 0.28 (− 0.58, 0.06)
	ERR_H_/Gy	**0.08 (+ 0.00, 0.17)**	**0.12 (0.02, 0.25)**	**0.11 (0.02, 0.23)**
	*p* value^c^	0.154	0.324	**0.032**
0.045	ERR_L_/Gy	− 0.07 (− 0.34, 0.24)	0.05 (− 0.27, 0.42)	− 0.19 (− 0.46, 0.13)
	ERR_H_/Gy	0.07 (− 0.00, 0.16)	**0.11 (0.01, 0.24)**	**0.11 (0.01, 0.23)**
	*p* value^c^	0.358	> 0.50	0.082
0.050	ERR_L_/Gy	− 0.03 (− 0.28, 0.26)	0.10 (− 0.20, 0.45)	− 0.11 (− 0.37, 0.19)
	ERR_H_/Gy	0.07 (− 0.01, 0.16)	**0.11 (+ 0.00, 0.23)**	**0.10 (+ 0.00, 0.23)**
	*p* value^c^	> 0.50	> 0.50	0.179

Numbers in bold indicate significant differences.

*ERR/Gy* excess relative risk per unit gray of gamma-ray dose, *IHD* ischemic heart disease (ICD-9 codes: 410− 414).

^a^Unmonitored for plutonium alpha activity workers divided into two subgroups: only workers of reactors and the rest of unmonitored workers.

^b^For all workers.

^c^Likelihood ratio test comparing the models with and without cutpoint.

Meanwhile the exclusion of the adjustment for alpha dose from the model resulted in the decrease of the ERR_H_/Gy due to higher dose rates (by 30–35%) and even in the loss of significance at two dose rate cutpoints (0.045 and 0.050 Gy/year). In contrast, such exclusion of this adjustment resulted in the increase of risk estimates due to lower dose rates at all cutpoints (> 10%) without changes in significance of the risk estimates (the resident subcohort, Table 7). In males of the resident subcohort the exclusion of the adjustment for alpha dose from the model did not change markedly the magnitude of the IHD mortality risk due to higher dose rates but at certain cutpoints (0.010, 0.025, 0.040, 0.045 and 0.050 Gy/year) the risk gained significance (Table S20). In females of the resident subcohort the exclusion of the adjustment for alpha dose considerably changed the magnitude of the risk estimate due to higher dose rates and the risk became negative, but non-significant, at every dose rate cutpoint (Table S21).

The sensitivity analysis performed with the model that included the alternative adjustment for alpha dose demonstrated the increase in IHD mortality risks due to both higher and lower dose rates at every cutpoint for both sexes (Table 7) and for males in the resident subcohort (Table S20). In females the risk estimate remained stable when analyzed with the model including the alternative alpha dose adjustment (Table S21).

The consideration of the limited dataset that included only those workers who had worked at the Mayak PA for > 1 year (the sensitivity analysis for which workers with duration of employment < 1 year were excluded from the analyzed dataset) did not affect considerably the result for both sexes (Table 8), males (Table S22) and females (Table S23) in the resident subcohort.

**Table 8:** Excess relative risk per Gy of IHD mortality in relation to 10-year lagged cumulative liver absorbed gamma-ray doses from external exposure (sensitivity analyses—dataset restricted and additional inclusion of the adjustment, both sexes, residents).

Cutpoint, Gy/year	Model parameters	Sensitivity analysis type		
		Dataset restricted to workers employed longer than one year	Inclusion of the adjustment for period of hire	Inclusion of the adjustment for age at hire
0 (without cutpoint^16^)	ERR/Gy	0.08 (− 0.01, 0.19)	0.08 (− 0.02, 0.20)	**0.12 (0.01, 0.25)**
0.005	ERR_L_/Gy	**− 5.08 (− 6.94, − 2.89)**	**− 4.51 (− 6.49, − 2.17)**	**− 4.53 (− 6.60, − 2.07)**
	ERR_H_/Gy	0.06 (− 0.02, 0.16)	0.06 (− 0.02, 0.17)	0.10 (− 0.00, 0.22)
	*p* value^a^	**< 0.001**	**< 0.001**	**< 0.001**
0.010	ERR_L_/Gy	**− 2.95 (− 3.79, − 1.99)**	**–2.88 (− 3.74, − 1.90)**	**− 2.85 (− 3.75, − 1.81)**
	ERR_H_/Gy	0.07 (− 0.01, 0.17)	0.08 (− 0.01, 0.18)	**0.11 (0.01, 0.23)**
	*p* value^a^	**< 0.001**	**< 0.001**	**< 0.001**
0.015	ERR_L_/Gy	**− 2.03 (− 2.61, − 1.36)**	**− 2.02 (− 2.60, − 1.34)**	**− 2.04 (− 2.65, − 1.33)**
	ERR_H_/Gy	**0.09 (+ 0.00, 0.19)**	0.09 (− 0.00, 0.20)	**0.12 (0.02, 0.25)**
	*p* value^a^	**< 0.001**	**< 0.001**	**< 0.001**
0.020	ERR_L_/Gy	**− 1.39 (− 1.85, − 0.85)**	**− 1.43 (− 1.89, − 0.89)**	**− 1.48 (− 1.95, − 0.93)**
	ERR_H_/Gy	**0.11 (0.02, 0.21)**	**0.11 (0.01, 0.22)**	**0.15 (0.04, 0.28)**
	*p* value^a^	**< 0.001**	**< 0.001**	**< 0.001**
0.025	ERR_L_/Gy	**− 1.02 (− 1.41, − 0.57)**	**− 1.11 (− 1.49, − 0.66)**	**− 1.13 (− 1.53, − 0.67)**
	ERR_H_/Gy	**0.12 (0.03, 0.23)**	**0.12 (0.02, 0.24)**	**0.16 (0.05, 0.30)**
	*p* value^a^	**< 0.001**	**< 0.001**	**< 0.001**
0.030	ERR_L_/Gy	**− 0.68 (− 1.04, − 0.27)**	**− 0.79 (− 1.13, − 0.38)**	**− 0.83 (− 1.18, − 0.41)**
	ERR_H_/Gy	**0.12 (0.03, 0.24)**	**0.13 (0.03, 0.25)**	**0.17 (0.06, 0.32)**
	*p* value^a^	**< 0.001**	**< 0.001**	**< 0.001**
0.035	ERR_L_/Gy	**− 0.43 (− 0.76, − 0.05)**	**− 0.54 (− 0.86, − 0.16)**	**− 0.60 (− 0.92, − 0.21)**
	ERR_H_/Gy	**0.12 (0.02, 0.24)**	**0.13 (0.02, 0.25)**	**0.18 (0.06, 0.33)**
	*p* value^a^	**0.008**	**0.002**	**< 0.001**
0.040	ERR_L_/Gy	− 0.29 (− 0.58, 0.06)	**− 0.39 (− 0.68, − 0.05)**	**− 0.45 (− 0.74, − 0.10)**
	ERR_H_/Gy	**0.12 (0.02, 0.24)**	**0.13 (0.02, 0.26)**	**0.18 (0.06, 0.33)**
	*p* value^a^	**0.031**	**0.006**	**0.001**
0.045	ERR_L_/Gy	− 0.16 (− 0.44, 0.16)	− 0.26 (− 0.53, 0.06)	**− 0.32 (− 0.60, − 0.00)**
	ERR_H_/Gy	**0.11 (0.01, 0.23)**	**0.12 (0.02, 0.25)**	**0.18 (0.06, 0.34)**
	*p* value^a^	0.116	**0.029**	**0.006**
0.050	ERR_L_/Gy	− 0.10 (− 0.36, 0.20)	− 0.20 (− 0.46, 0.10)	− 0.26 (− 0.51, 0.05)
	ERR_H_/Gy	**0.10 (+ 0.00, 0.23)**	**0.12 (0.01, 0.25)**	**0.18 (0.06, 0.34)**
	*p* value^a^	0.205	0.053	**0.012**

Numbers in bold indicate significant differences.

*ERR/Gy* excess relative risk per unit gray of gamma-ray dose, *IHD* ischemic heart disease (ICD-9 codes: 410–414).

^a^Likelihood ratio test comparing the models with and without cutpoint.

Inclusion of an additional adjustment for the hire period in the model resulted in modest changes in IHD mortality risk estimates (with widening of the corresponding confidence intervals) both due to lower and higher dose rates at all cutpoints for both sexes (Table 8), males (Table S22) and females (Table S23) in the resident subcohort. This sensitivity analysis revealed significant differences in risk estimates between the conventional model and the alternative model at cutpoints 0.045 Gy/year for both sexes, and 0.040 and 0.045 Gy/year for males in the resident subcohort.

The sensitivity analysis that included the adjustment for age at hire in the model demonstrated significant differences between the conventional model and the alternative model at all cutpoints, whereas significantly increased IHD mortality risks were observed only due to higher dose rates at all cutpoints (excluding 0.005 Gy/year). Moreover, ERR_H_/Gy considerably increased (by 30–80%) for both sexes in the resident subcohort (Table 6), and the similar results were observed also in males (Table S21). In females the inclusion of the adjustment for age at hire in the model resulted in a considerable (three–fourfold) increase in ERR_H_/Gy due to higher dose rate and the risks gained significance at every cutpoint (Table S23).

The comparison between the IHD mortality risk in relation to dose rate provided with the linear model and corresponding estimates provided with alternative models did not demonstrate significant differences at any cutpoints for both sexes (Table 9), males (Table S24) and females (Table S25) in the resident subcohort.

**Table 9:** Excess relative risk per Gy of IHD mortality in relation to 10-year lagged cumulative liver absorbed gamma-ray doses from external exposure (non-linear analysis, both sexes, residents).

Cutpoint, Gy/year	Model parameters	Models^a^			
		(1)	(2)^b^	(3)^b^	(4)^b^
0.005	β_L1_	**− 4.91 (− 6.78, − 2.72)**	**− 6.62 (− 12.30, − 0.94)**	**− 4.91 (− 6.95, − 2.86)**	**− 6.57 (− 12.23, − 0.91)**
	β_H1_	0.06 (− 0.02, 0.16)	0.06 (− 0.03, 0.15)	0.03 (− 0.15, 0.22)	0.03 (− 0.15, 0.22)
	β_L2_	–	28.53 (− 61.85, 118.90)	–	27.84 (− 62.16, 117.80)
	β_H2_	–	–	0.01 (− 0.06, 0.08)	0.01 (− 0.06, 0.08)
	*p* value^c^	–	> 0.50	> 0.50	> 0.50
0.010	β_L1_	**− 2.85 (− 3.69, − 1.89)**	− 1.58 (− 4.39, 1.24)	**− 2.86 (− 3.76, − 1.97)**	− 1.58 (− 4.41, 1.24)
	β_H1_	0.07 (− 0.01, 0.17)	0.08 (− 0.01, 0.17)	0.09 (− 0.09, 0.27)	0.09 (− 0.10, 0.27)
	β_L2_	–	− 10.49 (− 31.57, 10.58)	–	− 10.48 (− 31.59, 10.63)
	β_H2_	–	–	− 0.01 (− 0.08, 0.07)	− 0.00 (− 0.08, 0.07)
	*p* value^c^	–	0.346	> 0.50	> 0.50
0.015	β_L1_	**− 1.96 (− 2.54, − 1.29)**	− 1.34 (− 3.34, 0.67)	**− 1.99 (− 2.62, − 1.35)**	− 1.37 (− 3.38, 0.65)
	β_H1_	**0.09 (+ 0.00, 0.19)**	0.09 (− 0.00, 0.18)	0.13 (− 0.05, 0.31)	0.13 (− 0.06, 0.31)
	β_L2_	–	− 3.59 (− 14.18, 7.01)	–	− 3.56 (− 14.21, 7.08)
	β_H2_	–	–	− 0.02 (− 0.09, 0.05)	− 0.02 (− 0.09, 0.05)
	*p* value^c^	–	> 0.50	> 0.50	> 0.50
0.020	β_L1_	**− 1.35 (− 1.81, − 0.82)**	**− 1.89 (− 3.40, − 0.38)**	**− 1.39 (− 1.91, − 0.87)**	**− 1.93 (− 3.45, − 0.40)**
	β_H1_	**0.11 (0.02, 0.21)**	**0.10 (0.01, 0.20)**	0.17 (− 0.02, 0.36)	0.17 (− 0.02, 0.35)
	β_L2_	–	2.29 (− 3.94, 8.52)	–	2.30 (− 3.96, 8.56)
	β_H2_	–	–	− 0.03 (− 0.10, 0.04)	− 0.03 (− 0.10, 0.04)
	*p* value^c^	–	0.467	0.42	> 0.50
0.025	β_L1_	**− 0.99 (− 1.38, − 0.54)**	− 1.12 (− 2.36, 0.12)	**− 1.03 (− 1.47, − 0.60)**	− 1.17 (− 2.42, 0.08)
	β_H1_	**0.12 (0.03, 0.23)**	**0.12 (0.02, 0.21)**	**0.20 (+ 0.00, 0.39)**	**0.20 (+ 0.00, 0.39)**
	β_L2_	–	0.45 (− 3.54, 4.44)	–	0.47 (− 3.55, 4.48)
	β_H2_	–	–	− 0.04 (− 0.11, 0.04)	− 0.04 (− 0.11, 0.04)
	*p* value^c^	–	> 0.50	0.329	> 0.50
0.030	β_L1_	**− 0.66 (− 1.02, − 0.26)**	− 0.92 (− 2.00, 0.16)	**− 0.70 (− 1.10, − 0.31)**	− 0.96 (− 2.05, 0.12)
	β_H1_	**0.12 (0.03, 0.23)**	**0.12 (0.02, 0.22)**	**0.20 (+ 0.00, 0.40)**	0.20 (− 0.00, 0.40)
	β_L2_	–	0.74 (− 2.20, 3.68)	–	0.74 (− 2.21, 3.69)
	β_H2_	–	–	− 0.04 (− 0.12, 0.04)	− 0.04 (− 0.12, 0.04)
	*p* value^c^	–	> 0.50	0.332	> 0.50
0.035	β_L1_	**− 0.42 (− 0.74, − 0.04)**	− 0.63 (− 1.57, 0.31)	**− 0.45 (− 0.81, − 0.10)**	− 0.66 (− 1.60, 0.29)
	β_H1_	**0.12 (0.02, 0.23)**	**0.12 (0.01, 0.22)**	0.20 (− 0.01, 0.41)	0.20 (− 0.01, 0.40)
	β_L2_	–	0.51 (− 1.65, 2.66)	–	0.49 (− 1.67, 2.65)
	β_H2_	–	–	− 0.04 (− 0.12, 0.04)	− 0.04 (− 0.12, 0.04)
	*p* value^c^	–	> 0.50	0.346	> 0.50
0.040	β_L1_	− 0.28 (− 0.58, 0.06)	− 0.64 (− 1.44, 0.15)	− 0.31 (− 0.64, 0.02)	− 0.68 (− 1.47, 0.12)
	β_H1_	**0.11 (0.02, 0.23)**	**0.11 (0.01, 0.21)**	0.20 (− 0.02, 0.41)	0.19 (− 0.02, 0.40)
	β_L2_	–	0.76 (− 0.81, 2.32)	–	0.76 (− 0.82, 2.33)
	β_H2_	–	–	− 0.04 (− 0.12, 0.04)	− 0.04 (− 0.12, 0.04)
	*p* value^c^	–	0.341	0.367	0.424
0.045	β_L1_	− 0.16 (− 0.43, 0.16)	− 0.52 (− 1.22, 0.18)	− 0.19 (− 0.49, 0.12)	− 0.55 (− 1.25, 0.16)
	β_H1_	**0.11 (0.01, 0.23)**	**0.10 (+ 0.00, 0.21)**	0.19 (− 0.03, 0.41)	0.19 (− 0.02, 0.40)
	β_L2_	–	0.65 (− 0.55, 1.85)	–	0.66 (− 0.55, 1.86)
	β_H2_	–	–	− 0.04 (− 0.12, 0.05)	− 0.04 (− 0.12, 0.04)
	*p* value^c^	–	0.285	0.375	0.376
0.050	β_L1_	− 0.10 (− 0.36, 0.20)	− 0.48 (− 1.13, 0.17)	− 0.13 (− 0.41, 0.16)	− 0.51 (− 1.17, 0.14)
	β_H1_	**0.10 (+ 0.00, 0.22)**	0.10 (− 0.00, 0.20)	0.19 (− 0.03, 0.41)	0.19 (− 0.03, 0.40)
	β_L2_	–	0.63 (− 0.39, 1.65)	–	0.64 (− 0.38, 1.66)
	β_H2_	–	–	− 0.04 (− 0.12, 0.04)	− 0.04 (− 0.12, 0.04)
	*p* value^c^	–	0.229	0.361	0.312

Numbers in bold indicate significant differences. The dataset for the analysis was stratified by sex, attained age, calendar period, smoking status, alcohol consumption, alpha dose.

*ERR/Gy* excess relative risk per unit gray of gamma-ray dose, *IHD* ischemic heart disease (ICD-9 codes: 410–414).

^a^Equations of the models: (1) λ = λ_0_(1 + β_L1_D_L_ + β_H1_D_H_), (2) λ = λ_0_(1 + β_L1_D_L_ + β_L2_D_L_^2^ + β_Η1_D_H_), (3) λ = λ_0_(1 + β_L1_D_L_ + β_Η1_D_H_ + β_Η2_D_H_^2^), (4) λ = λ_0_(1 + β_L1_D_L_ + β_L2_D_L_^2^ + β_Η1_D_H_ + β_Η2_D_H_^2^).

^b^Wald-type confidence interval.

^c^*p* value denotes significant differences from a linear model.

## Discussion

For improvement of the radiation protection system it is essential to consider factors that modify the dose–response relationship^9,10^, and dose rate is among such factors. This study examined the impact of dose rate (in terms of annual dose rate) on IHD mortality among chronically exposed Russian nuclear workers. Significantly increased excess relative risks of IHD mortality per unit of total external gamma-ray dose accumulated at higher dose rates were observed for both sexes at 0.015–0.050 Gy/year and for males at 0.020–0.035 Gy/year in the resident subcohort. In females the estimates of ERR/Gy of the cumulative dose for IHD mortality were non-significantly increased due to higher dose rates compared to lower dose rates at all cutpoints of annual doses. There were, however, no significant differences between sexes.

The ERR_L_/Gy and ERR_H_/Gy for IHD mortality increased with increasing dose rate cutpoints from 0.005 through 0.050 Gy/year and the confidence intervals became narrower due to the increment of the number of person-years of the follow-up that corresponded to high dose rates that exceeded occupational annual dose limits^19,21,22^. It should be noted that uninterrupted external high gamma-dose rate exposure over 5 years resulted in a notable (3–4.5 fold) increase in the IHD mortality risk compared to exposure at similar dose rates over 1 year (EER_H5_/Gy > ERR_H_/Gy, *p* < 0.001).

In this study lagging of the gamma-ray dose affected the risk estimate, consistent with the previous study^16^. The increase in the lag period resulted in the decrease (almost two-fold) in the IHD mortality risk due to higher dose rates (ERR_H_/Gy) at all cutpoints except for 0.005 Gy/year, and even to the loss of significance with 20 and 30-year lagging. In our opinion, the observed result was not attributable to the loss of higher dose rates since Mayak workers had been exposed at higher dose rates in early years after hire (Fig. 2). In this study the conventional 10-year lag was used; however, there is ongoing discussion on an appropriate lag period for certain causes of death from non-cancer diseases including IHD.

Neither the conventional model (without considering dose rate) nor the alternative model (considering dose rate) revealed the effect of adjusting for neutron dose on the IHD mortality risk following chronic external gamma-ray exposure.

In contrast, the adjustment for alpha dose (exclusion from the model and the alternative adjusting) changed the ERR/Gy estimates for IHD mortality regardless of whether the conventional model or the alternative model was used. This is why in order to provide more precise and less uncertain risk estimates all radiation types should be considered in analyzing radiogenic risks in individual cohort members exposed to combined radiation.

Experimental studies have shown both sparing and enhancing (inverse) dose protraction effects of radiation exposure on the circulatory system^23–29^, and consensus has not yet been reached regarding dose rate effectiveness^30,31^. Recently Kloosterman and colleagues^32^ have developed a biophysical mathematical model to describe the radiation-promoted atheroslerotic plague development. The authors state that with the adequate experimental data available this model could be further elaborated to take into account the dose rate effect.

In the meantime, studies of dose rate effects on risks of radiation-related health outcomes in human cohorts are very limited^5–7^. On the one hand, there are indications of larger risks per unit dose for lower dose rate and fractionated exposures^33,34^. On the other hand, it should be noted that the results and conclusions of this study of IHD mortality in the cohort of the Russian nuclear Mayak workers are overall in good agreement with those observed in the study of UK nuclear workers of the Hanford site^7^ that gives evidence for an increase in the ERR/Gy estimates at higher dose rates. This is why to improve the radiological protection system it is highly important to continue studies of cancer and non-cancer risks taking into account a dose rate in addition to non-radiation confounding factors and cumulative dose, as well as mechanistic studies for outcome development due to exposures at different dose rates.

This study has a number of strengths: the large size of the Mayak worker cohort (22,377 individuals) and the resident subcohort (13,156 individuals); availability of individual annual gamma-ray doses from external exposure measured with individual film badges over the whole follow-up period; the long follow-up period (70 years); the available vital status (96%) of cohort members, high quality of data on causes of death; available information on acknowledged confounders (e.g., smoking, alcohol consumption that were taken into account in the present study, hypertension, high body mass index); available biological specimens including heart tissues that enable investigation of outcome mechanisms due to chronic radiation exposure^35,36^.

The limitation of this study includes the lack of data on temporal radiation dose distributions in the MWDS-2013 precisely enough to calculate hourly or daily dose rate, wherefore we employed annual dose rate. In addition, it should be noted that alpha actvity was measured in bioassays for only 44.8% of Mayak workers who could have been affected by aerosols containing alpha particles (workers of the radiochemical and plutonium production plants). Despite the fact that dosimetry systems for Mayak PA workers have been updated and improved over many years within the Russian-American cooperation^37^, considerable uncertainties remain in the dose estimates from external and internal exposures. This study used point dose estimates provided by MWDS-2013, and did not consider uncertainties in external gamma and neutron or internal alpha particle dose estimates.

The limitations of this study were the small number of migrants in the Mayak worker cohort whose complete medical information or data on confounding factors were unavailable, and also the low statistical power of the analysis that considered females separately due to the smaller number of females in the Mayak worker cohort and even smaller in the resident subcohort.

## Conclusions

The results of this study provide evidence supporting associations of dose rate and duration of uninterrupted high dose rate exposure with the ERR/Gy estimates for IHD mortality in chronically exposed workers. The observed findings are in good agreement with findings of other studies and considerably contribute to the scientific basis for recommendations of the radiation protection system.

## Supplementary Information

Supplementary Information.

## Data Availability

The dataset is the intellectual property of the Southern Urals Biophysics Institute, Ozyorsk, Chelyabinsk Region, 456,780, Russia. For privacy reasons it is not publicly available. These restrictions on data availability are imposed by Federal Act No. 323 of 21 November 2011 on the basics of health care for Russian citizens and Federal Act No. 152 of 27 July 2014 on personal data. Any access to the Mayak Worker Cohort must be approved by the institutional Ethics Review Board of the Southern Urals Biophysics Institute. To request the data used in the presented analyses, contact Drs. Tamara Azizova (the head of the clinical department of the Southern Urals Biophysics Institute) and Valentina Rybkina (leading researcher of the Southern Urals Biophysics Institute, member of the institutional Ethics Review Boar, rybkina@subi.su).

## References

[1] ICRP (2022). Radiation detriment calculation methodology. ICRP publication 152. Ann ICRP.

[2] Ban N, Cléro E, Vaillant L, Zhang W, Hamada N, Preston D, Laurier D (2022). Radiation detriment calculation methodology: Summary of ICRP Publication 152. J. Radiol. Prot..

[3] Preston DL (2007). Solid cancer incidence in atomic bomb survivors: 1958–1998. Radiat. Res..

[4] Rühm W (2018). Typical doses and dose rates in studies pertinent to radiation risk inference at low doses and low dose rates. J. Radiat. Res..

[5] Metz-Flamant C, Samson E, Caër-Lorho S, Acker A, Laurier D (2012). Leukemia risk associated with chronic external exposure to ionizing radiation in a French cohort of nuclear workers. Radiat. Res..

[6] Sasaki M, Kudo S, Furuta H (2019). Effect of radiation dose rate on cancer mortality among nuclear workers: Reanalysis of Hanford data. Health Phys..

[7] Sasaki M, Kudo S, Furuta H (2020). Effect of radiation dose rate on circulatory disease mortality among nuclear workers: Reanalysis of Hanford data. Health Phys..

[8] Boice JD (2022). Mortality from leukemia cancer and heart disease among U.S. nuclear power plant workers, 1957–2011. Int. J. Radiat. Biol..

[9] Clement C (2021). Keeping the ICRP recommendations fit for purpose. J. Radiol. Prot..

[10] Laurier D, Rühm W, Paquet F, Applegate K, Cool D, Clement C, International Commission on Radiological Protection (ICRP) (2021). Areas of research to support the system of radiological protection. Radiat. Environ. Biophys..

[11] Kruglov A (2002). The History of the Soviet Atomic Industry.

[12] WHO (1980). ICD-9 Guidelines for Coding Diseases, Injuries and Causes of Death/Revision 1975.

[13] Azizova TV (2008). The "clinic" medical-dosimetric database of Mayak Production Association workers: Structure, characteristics and prospects of utilization. Health Phys..

[14] Napier BA (2017). The Mayak worker dosimetry system (MWDS-2013): An introduction to the documentation. Radiat. Prot. Dosim..

[15] Birchall A (2017). The Mayak worker dosimetry system (MWDS-2013) for internally deposited plutonium: An overview. Radiat. Prot. Dosim..

[16] Azizova TV, Bannikova MV, Grigoryeva ES, Briks KV, Hamada N (2022). Mortality from various diseases of the circulatory system in the Russian Mayak nuclear worker cohort: 1948–2018. J. Radiol. Prot..

[17] Preston D, Lubin J, Pierce D, McConney M (1993). Epicure Users Guide.

[18] Furuta, H., Kudo, S., Ishida, J., Yoshimoto, K. & Kasagi, F. Dose-rate effects on cancer mortality risk estimates for Japanese nuclear workers. In *Proceedings of the 2nd European Radiological Protection Research Week, Paris*. Available at: http://www.rea.or.jp/ire/pdf/20171010Furuta.pdf. Accessed 21 March 2022 (2017).

[19] ICRP (2007). The 2007 recommendations of the international commission on radiological protection. ICRP publication 103. Ann ICRP.

[20] Vasilenko EK (2001). Verification if individual doses from external radiation exposure to workers of PA “Mayak” (methods and results). Med. Radiol. Radiat. Saf..

[21] SanPiN 2.6.1.2523–09. Radiation Safety Standards (NRB-99/2009). **(In Russ.)**.

[22] *Radiation Protection and Safety of Radiation Sources: International Basic Safety Standards*. IAEA Safety Standards Series No. GSR Part 3 (IAEA, Vienna, 2014).

[23] Mitchel RE (2013). Low-dose radiation exposure and protection against atherosclerosis in ApoE(-/-) mice: The influence of P53 heterozygosity. Radiat. Res..

[24] Mancuso M (2015). Acceleration of atherogenesis in ApoE^−^^/^^−^ mice exposed to acute or low-dose-rate ionizing radiation. Oncotarget.

[25] Andreassi MG (2015). Subclinical carotid atherosclerosis and early vascular aging from long-term low-dose ionizing radiation exposure: A genetic, telomere, and vascular ultrasound study in cardiac catheterization laboratory staff. JACC Cardiovasc. Interv..

[26] Cervelli T (2014). Effects of single and fractionated low-dose irradiation on vascular endothelial cells. Atherosclerosis.

[27] Hoel DG, Carnes BA (2017). Cardiovascular effects of fission neutron or ^60^Co γ exposure in the B6CF_1_ mouse. Int. J. Radiat. Biol..

[28] Tran V, Little MP (2017). Dose and dose rate extrapolation factors for malignant and non-malignant health endpoints after exposure to gamma and neutron radiation. Radiat. Environ. Biophys..

[29] Hamada N (2021). Vascular damage in the aorta of wild-type mice exposed to ionizing radiation: Sparing and enhancing effects of dose protraction. Cancers (Basel).

[30] Tapio S (2021). Ionizing radiation-induced circulatory and metabolic diseases. Environ. Int..

[31] Little MP, Azizova TV, Hamada N (2021). Low- and moderate-dose non-cancer effects of ionizing radiation in directly exposed individuals, especially circulatory and ocular diseases: A review of the epidemiology. Int. J. Radiat. Biol..

[32] Kloosterman A (2017). How radiation influences atherosclerotic plaque development: A biophysical approach in ApoE^−^^/^^−^ mice. Radiat. Environ. Biophys..

[33] Zablotska LB, Little MP, Cornett RJ (2014). Potential increased risk of ischemic heart disease mortality with significant dose fractionation in the Canadian Fluoroscopy Cohort Study. Am. J. Epidemiol..

[34] Little MP (2016). Radiation and circulatory disease. Mutat. Res. Rev. Mutat. Res..

[35] Azimzadeh O (2020). Chronic occupational exposure to ionizing radiation induces alterations in the structure and metabolism of the heart: A proteomic analysis of human formalin-fixed paraffin-embedded (FFPE) cardiac tissue. Int. J. Mol. Sci..

[36] Papiez A (2018). Integrative multiomics study for validation of mechanisms in radiation-induced ischemic heart disease in Mayak workers. PLoS ONE.

[37] Fountos BN (2017). The department of energy's russian health studies program. Radiat. Prot. Dosim..

