# Supplementary material for: Dose rate effect on mortality from ischemic heart disease in the cohort of Russian Mayak Production Association workers
Source: Sci Rep. 2023 Feb 2;13:1926. doi: 10.1038/s41598-023-28954-w (PMC9895442; doi:10.1038/s41598-023-28954-w)
Supplement: Supplementary file 1 — Supplementary Information. [file 41598_2023_28954_MOESM1_ESM.docx]

Tamara V. Azizova, Evgeniya S. Grigoryeva, Nobuyuki Hamada

**Dose rate effect on mortality from ischemic heart disease in the cohort of Mayak Production Association workers**

**Supplementary Information**

Abbreviations:

ERR, excess relative risk;

Gy, gray;

IHD, ischemic heart disease (ICD-9 codes: 410–414);

Table S1 Excess relative risk per Gy of IHD mortality in relation to 10-year lagged cumulative liver absorbed gamma-ray doses from external radiation exposure, adjusted for various non-radiation factors and alpha absorbed dose to the liver (main analysis, entire cohort)

| Cutpoint,  Gy/year | Model  parameters | Both sexes | Males | Females | *p* value ^a^ |
| --- | --- | --- | --- | --- | --- |
| 0 (without cutpoint [16]) | ERR/Gy | 0.04 (–0.02, 0.11) | 0.02 (–0.05, 0.09) | 0.14 (–0.02, 0.37) | 0.197 |
| 0.005 | ERR_L_/Gy | **–4.71 (–6.58, –2.56)** | **–5.06 (–7.05, –2.72)** | –3.13 (–7.51, 2.70) | 0.318 |
|  | ERR_H_/Gy | 0.03 (–0.03, 0.10) | 0.01 (–0.05, 0.08) | 0.14 (–0.03, 0.35) |  |
|  | *p* value ^b^ | **< 0.001** | **< 0.001** | 0.241 |  |
| 0.010 | ERR_L_/Gy | **–2.77 (–3.59, –1.84)** | **–2.91 (–3.81, –1.88)** | –2.20 (–4.00, 0.14) | 0.353 |
|  | ERR_H_/Gy | 0.04 (–0.02, 0.11) | 0.02 (–0.04, 0.09) | 0.14 (–0.02, 0.35) |  |
|  | *p* value ^b^ | **< 0.001** | **< 0.001** | **0.049** |  |
| 0.015 | ERR_L_/Gy | **–1.98 (–2.53, –1.36)** | **–2.07 (–2.67, –1.39)** | –1.58 (–2.85, 0.06) | 0.328 |
|  | ERR_H_/Gy | 0.05 (–0.01, 0.11) | 0.03 (–0.03, 0.10) | 0.15 (–0.01, 0.37) |  |
|  | *p* value ^b^ | **< 0.001** | **< 0.001** | **0.039** |  |
| 0.020 | ERR_L_/Gy | **–1.37 (–1.80, –0.88)** | **–1.38 (–1.85, –0.85)** | –1.26 (–2.27, 0.05) | 0.454 |
|  | ERR_H_/Gy | 0.06 (–0.01, 0.13) | 0.04 (–0.03, 0.11) | 0.16 (–0.01, 0.38) |  |
|  | *p* value ^b^ | **< 0.001** | **< 0.001** | **0.035** |  |
| 0.025 | ERR_L_/Gy | **–0.98 (–1.35, –0.57)** | **–0.96 (–1.35, –0.50)** | –1.03 (–1.88, 0.10) | > 0.50 |
|  | ERR_H_/Gy | 0.06 (–0.00, 0.13) | 0.04 (–0.02, 0.12) | 0.16 (–0.01, 0.38) |  |
|  | *p* value ^b^ | **< 0.001** | **< 0.001** | **0.041** |  |
| 0.030 | ERR_L_/Gy | **–0.69 (–1.02, –0.33)** | **–0.70 (–1.04, –0.30)** | –0.59 (–1.42, 0.49) | > 0.50 |
|  | ERR_H_/Gy | 0.06 (–0.00, 0.14) | 0.05 (–0.02, 0.13) | 0.15 (–0.01, 0.37) |  |
|  | *p* value ^b^ | **< 0.001** | **< 0.001** | 0.159 |  |
| 0.035 | ERR_L_/Gy | **–0.49 (–0.78, –0.16)** | **–0.47 (–0.78, –0.11)** | –0.54 (–1.30, 0.45) | > 0.50 |
|  | ERR_H_/Gy | 0.06 (–0.00, 0.14) | 0.04 (–0.03, 0.13) | 0.16 (–0.01, 0.38) |  |
|  | *p* value ^b^ | **0.002** | **0.008** | 0.151 |  |
| 0.040 | ERR_L_/Gy | **–0.36 (–0.62, –0.07)** | **–0.35 (–0.62, –0.03)** | –0.39 (–1.12, 0.55) | > 0.50 |
|  | ERR_H_/Gy | 0.06 (–0.00, 0.14) | 0.04 (–0.03, 0.12) | 0.16 (–0.01, 0.38) |  |
|  | *p* value ^b^ | **0.008** | **0.022** | 0.228 |  |
| 0.045 | ERR_L_/Gy | –0.25 (–0.48, 0.03) | –0.24 (–0.49, 0.05) | –0.24 (–0.95, 0.69) | > 0.50 |
|  | ERR_H_/Gy | 0.06 (–0.01, 0.14) | 0.04 (–0.03, 0.12) | 0.16 (–0.01, 0.38) |  |
|  | *p* value ^b^ | **0.037** | 0.073 | 0.373 |  |
| 0.050 | ERR_L_/Gy | –0.18 (–0.40, 0.08) | –0.17 (–0.41, 0.11) | –0.19 (–0.88, 0.70) | 0.493 |
|  | ERR_H_/Gy | 0.06 (–0.01, 0.13) | 0.04 (–0.03, 0.12) | 0.15 (–0.02, 0.38) |  |
|  | *p* value ^b^ | 0.088 | 0.159 | 0.411 |  |
| *Notes:*  Numbers in bold indicate significant differences. The dataset for the analysis was stratified by sex, attained age, calendar period, smoking status, alcohol consumption, and alpha dose.  CI, confidence interval. ERR/Gy, excess relative risk per unit gray of gamma-ray dose. IHD, ischemic heart disease (ICD-9 codes: 410–414). n/a denotes that it was not possible to estimate the lower or upper bound of the confidence interval.  ^a^ Test for heterogeneity between sexes.  ^b^ Likelihood ratio test comparing the models with and without cutpoint. | | | | | |

Figure S1 Excess relative risk per Gy of IHD mortality in the entire cohort in relation to 10-year lagged cumulative liver absorbed doses from external gamma-ray exposure, adjusted for various non-radiation factors and alpha absorbed dose to the liver (main analysis, entire cohort): a – both sexes, b – males, c – females.

Table S2 Excess relative risk per Gy of IHD mortality in relation to cumulative liver absorbed gamma-ray doses from external radiation exposure, adjusted for various non-radiation factors and alpha absorbed dose to the liver (sensitivity analyses – various lag periods, both sexes, entire cohort)

| Cutpoint,  Gy/year | Model  parameters | Lag periods | | | |
| --- | --- | --- | --- | --- | --- |
|  |  | 0 years | 5 years | 20 years | 30 years |
| 0 (without cutpoint) | ERR/Gy | 0.04 (–0.02, 0.11) [16] | 0.04 (–0.02, 0.11) | 0.04 (–0.03, 0.11) | 0.05 (–0.02, 0.13) |
| 0.005 | ERR_L_/Gy | **–5.56 (–6.98, –3.90)** | **–4.93 (–6.59, –3.01)** | **–4.10 (–6.83, –0.96)** | **–5.45 (–9.69, –0.41)** |
|  | ERR_H_/Gy | 0.03 (–0.03, 0.10) | 0.03 (–0.03, 0.10) | 0.03 (–0.03, 0.10) | 0.04 (–0.03, 0.12) |
|  | *p* value ^a^ | **< 0.001** | **< 0.001** | **0.012** | **0.034** |
| 0.010 | ERR_L_/Gy | **–3.06 (–3.76, –2.27)** | **–2.85 (–3.60, –1.99)** | **–2.81 (–3.86, –1.61)** | **–3.33 (–4.78, –1.63)** |
|  | ERR_H_/Gy | 0.05 (–0.01, 0.11) | 0.04 (–0.01, 0.11) | 0.03 (–0.03, 0.10) | 0.04 (–0.02, 0.12) |
|  | *p* value ^a^ | **< 0.001** | **< 0.001** | **< 0.001** | **< 0.001** |
| 0.015 | ERR_L_/Gy | **–2.13 (–2.61, –1.58)** | **–2.03 (–2.54, –1.45)** | **–2.00 (–2.67, –1.22)** | **–2.53 (–3.38, –1.53)** |
|  | ERR_H_/Gy | 0.06 (–0.00, 0.12) | 0.05 (–0.01, 0.12) | 0.04 (–0.02, 0.11) | 0.05 (–0.02, 0.13) |
|  | *p* value ^a^ | **< 0.001** | **< 0.001** | **< 0.001** | **< 0.001** |
| 0.020 | ERR_L_/Gy | **–1.48 (–1.87, –1.05)** | **–1.41 (–1.81, –0.95)** | **–1.41 (–1.91, –0.83)** | **–1.77 (–2.40, –1.03)** |
|  | ERR_H_/Gy | **0.07 (+0.00, 0.14)** | **0.06 (+0.00, 0.13)** | 0.05 (–0.02, 0.12) | 0.06 (–0.01, 0.14) |
|  | *p* value ^a^ | **< 0.001** | **< 0.001** | **< 0.001** | **< 0.001** |
| 0.025 | ERR_L_/Gy | **–1.08 (–1.41, –0.71)** | **–1.02 (–1.37, –0.63)** | **–0.96 (–1.39, –0.47)** | **–1.37 (–1.87, –0.78)** |
|  | ERR_H_/Gy | **0.07 (0.01, 0.15)** | **0.07 (0.01, 0.14)** | 0.05 (–0.01, 0.13) | 0.06 (–0.01, 0.15) |
|  | *p* value ^a^ | **< 0.001** | **< 0.001** | **< 0.001** | **< 0.001** |
| 0.030 | ERR_L_/Gy | **–0.79 (–1.09, –0.46)** | **–0.74 (–1.05, –0.39)** | **–0.70 (–1.07, –0.28)** | **–1.02 (–1.45, –0.51)** |
|  | ERR_H_/Gy | **0.08 (0.01, 0.15)** | **0.07 (0.01, 0.14)** | 0.06 (–0.01, 0.13) | 0.07 (–0.00, 0.15) |
|  | *p* value ^a^ | **< 0.001** | **< 0.001** | **0.001** | **< 0.001** |
| 0.035 | ERR_L_/Gy | **–0.59 (–0.85, –0.29)** | **–0.55 (–0.82, –0.24)** | **–0.50 (–0.83, –0.13)** | **–0.78 (–1.17, –0.33)** |
|  | ERR_H_/Gy | **0.08 (0.01, 0.15)** | **0.07 (0.01, 0.15)** | 0.06 (–0.01, 0.13) | 0.07 (–0.00, 0.15) |
|  | *p* value ^a^ | **< 0.001** | **< 0.001** | **0.006** | **< 0.001** |
| 0.040 | ERR_L_/Gy | **–0.45 (–0.69, –0.18)** | **–0.42 (–0.66, –0.14)** | **–0.37 (–0.67, –0.04)** | **–0.61 (–0.96, –0.21)** |
|  | ERR_H_/Gy | **0.08 (0.01, 0.15)** | **0.07 (+0.00, 0.15)** | 0.06 (–0.01, 0.13) | 0.07 (–0.00, 0.16) |
|  | *p* value ^a^ | **< 0.001** | **0.002** | **0.017** | **0.002** |
| 0.045 | ERR_L_/Gy | **–0.33 (–0.55, –0.08)** | **–0.30 (–0.52, –0.04)** | –0.25 (–0.52, 0.06) | **–0.45 (–0.77, –0.07)** |
|  | ERR_H_/Gy | **0.07 (0.01, 0.15)** | **0.07 (+0.00, 0.15)** | 0.05 (–0.01, 0.13) | 0.07 (–0.00, 0.16) |
|  | *p* value ^a^ | **0.004** | **0.01** | 0.061 | **0.009** |
| 0.050 | ERR_L_/Gy | **–0.25 (–0.46, –0.01)** | –0.22 (–0.44, 0.03) | –0.17 (–0.43, 0.12) | –0.35 (–0.65, +0.00) |
|  | ERR_H_/Gy | **0.07 (+0.00, 0.15)** | 0.06 (–0.00, 0.14) | 0.05 (–0.02, 0.13) | 0.07 (–0.00, 0.16) |
|  | *p* value ^a^ | **0.016** | **0.032** | 0.145 | **0.024** |
| *Notes:*  Numbers in bold indicate significant differences. The dataset for the analysis was stratified by sex, attained age, calendar period, smoking status, alcohol consumption, alpha dose.  CI, confidence interval. ERR/Gy, excess relative risk per unit gray of gamma-ray dose. IHD, ischemic heart disease (ICD-9 codes: 410–414). n/a denotes that it was not possible to estimate the lower or upper bound of the confidence interval.  ^a^ Likelihood ratio test comparing the models with and without cutpoint. | | | | | |

Table S3 Excess relative risk per Gy of IHD mortality in relation to cumulative liver absorbed gamma-ray doses from external radiation exposure, adjusted for various non-radiation factors and alpha absorbed dose to the liver (sensitivity analyses – various lag periods, males, entire cohort)

| Cutpoint,  Gy/year | Model  parameters | Lag periods | | | |
| --- | --- | --- | --- | --- | --- |
|  |  | 0 years | 5 years | 20 years | 30 years |
| 0 (without cutpoint) | ERR/Gy | 0.02 (–0.04, 0.10) [16] | 0.02 (–0.04, 0.10) | 0.02 (–0.05, 0.10) | 0.04 (–0.04, 0.13) |
| 0.005 | ERR_L_/Gy | **–5.88 (–7.31, –4.14)** | **–5.26 (–7.00, –3.21)** | **–4.55 (–7.60, –0.91)** | –5.93 (–11.13, 0.46) |
|  | ERR_H_/Gy | 0.01 (–0.05, 0.09) | 0.02 (–0.05, 0.09) | 0.01 (–0.05, 0.09) | 0.03 (–0.05, 0.12) |
|  | *p* value ^a^ | **< 0.001** | **< 0.001** | **0.016** | 0.066 |
| 0.010 | ERR_L_/Gy | **–3.21 (–3.95, –2.36)** | **–2.98 (–3.79, –2.05)** | **–3.09 (–4.27, –1.70)** | **–4.25 (–5.96, –2.18)** |
|  | ERR_H_/Gy | 0.03 (–0.03, 0.10) | 0.03 (–0.03, 0.10) | 0.02 (–0.05, 0.09) | 0.03 (–0.04, 0.11) |
|  | *p* value ^a^ | **< 0.001** | **< 0.001** | **< 0.001** | **< 0.001** |
| 0.015 | ERR_L_/Gy | **–2.20 (–2.71, –1.61)** | **–2.10 (–2.65, –1.47)** | **–2.18 (–2.93, –1.31)** | **–3.17 (na, –2.01)** |
|  | ERR_H_/Gy | 0.04 (–0.02, 0.11) | 0.04 (–0.03, 0.11) | 0.02 (–0.04, 0.10) | 0.03 (–0.04, 0.11) |
|  | *p* value ^a^ | **< 0.001** | **< 0.001** | **< 0.001** | **< 0.001** |
| 0.020 | ERR_L_/Gy | **–1.48 (–1.89, –1.01)** | **–1.41 (–1.85, –0.91)** | **–1.45 (–2.00, –0.80)** | **–2.00 (–2.71, –1.14)** |
|  | ERR_H_/Gy | 0.05 (–0.01, 0.13) | 0.05 (–0.02, 0.12) | 0.03 (–0.04, 0.11) | 0.04 (–0.03, 0.13) |
|  | *p* value ^a^ | **< 0.001** | **< 0.001** | **< 0.001** | **< 0.001** |
| 0.025 | ERR_L_/Gy | **–1.05 (–1.41, –0.64)** | **–1.00 (–1.37, –0.57)** | **–0.94 (–1.42, –0.39)** | **–1.44 (–2.01, –0.77)** |
|  | ERR_H_/Gy | 0.06 (–0.01, 0.14) | 0.05 (–0.01, 0.13) | 0.04 (–0.03, 0.12) | 0.05 (–0.02, 0.14) |
|  | *p* value ^a^ | **< 0.001** | **< 0.001** | **0.001** | **< 0.001** |
| 0.030 | ERR_L_/Gy | **–0.78 (–1.10, –0.43)** | **–0.74 (–1.06, –0.36)** | **–0.72 (–1.12, –0.25)** | **–1.10 (–1.58, –0.53)** |
|  | ERR_H_/Gy | 0.06 (–0.01, 0.14) | 0.06 (–0.01, 0.14) | 0.04 (–0.03, 0.12) | 0.06 (–0.02, 0.15) |
|  | *p* value ^a^ | **< 0.001** | **< 0.001** | **0.003** | **< 0.001** |
| 0.035 | ERR_L_/Gy | **–0.57 (–0.85, –0.25)** | **–0.53 (–0.82, –0.19)** | **–0.50 (–0.85, –0.09)** | **–0.82 (–1.24, –0.32)** |
|  | ERR_H_/Gy | 0.06 (–0.01, 0.15) | 0.06 (–0.01, 0.14) | 0.04 (–0.03, 0.12) | 0.06 (–0.02, 0.15) |
|  | *p* value ^a^ | **< 0.001** | **0.002** | **0.014** | **0.001** |
| 0.040 | ERR_L_/Gy | **–0.44 (–0.68, –0.15)** | **–0.40 (–0.66, –0.10)** | **–0.37 (–0.68, –0.01)** | **–0.63 (–1.01, –0.19)** |
|  | ERR_H_/Gy | 0.06 (–0.01, 0.14) | 0.05 (–0.02, 0.14) | 0.04 (–0.03, 0.12) | 0.06 (–0.02, 0.15) |
|  | *p* value ^a^ | **0.002** | **0.006** | **0.033** | **0.004** |
| 0.045 | ERR_L_/Gy | **–0.32 (–0.55, –0.05)** | **–0.29 (–0.52, –0.01)** | –0.25 (–0.54, 0.08) | **–0.46 (–0.80, –0.06)** |
|  | ERR_H_/Gy | 0.06 (–0.01, 0.14) | 0.05 (–0.02, 0.14) | 0.04 (–0.03, 0.12) | 0.06 (–0.02, 0.15) |
|  | *p* value ^a^ | **0.01** | **0.024** | 0.092 | **0.016** |
| 0.050 | ERR_L_/Gy | –0.23 (–0.45, 0.02) | –0.21 (–0.43, 0.05) | –0.17 (–0.44, 0.15) | –0.35 (–0.67, 0.03) |
|  | ERR_H_/Gy | 0.05 (–0.02, 0.14) | 0.05 (–0.02, 0.13) | 0.03 (–0.04, 0.12) | 0.06 (–0.02, 0.15) |
|  | *p* value ^a^ | **0.04** | 0.07 | 0.218 | **0.044** |
| *Notes:*  Numbers in bold indicate significant differences. The dataset for the analysis was stratified by sex, attained age, calendar period, smoking status, alcohol consumption, alpha dose.  CI, confidence interval. ERR/Gy, excess relative risk per unit gray of gamma-ray dose. IHD, ischemic heart disease (ICD-9 codes: 410–414). n/a denotes that it was not possible to estimate the lower or upper bound of the confidence interval.  ^a^ Likelihood ratio test comparing the models with and without cutpoint. | | | | | |

Table S4 Excess relative risk per Gy of IHD mortality in relation to cumulative liver absorbed gamma-ray doses from external radiation exposure, adjusted for various non-radiation factors and alpha absorbed dose to the liver (sensitivity analyses – various lag periods, females, entire cohort)

| Cutpoint,  Gy/year | Model  parameters | Lag periods | | | |
| --- | --- | --- | --- | --- | --- |
|  |  | 0 years | 5 years | 20 years | 30 years |
| 0 (without cutpoint) | ERR/Gy | 0.13 (–0.03, 0.34) [16] | 0.13 (–0.03, 0.35) | 0.12 (–0.04, 0.33) | 0.11 (–0.05, 0.34) |
| 0.005 | ERR_L_/Gy | –3.53 (–7.53, 1.81) | –3.22 (–7.40, 2.34) | –2.88 (–8.09, 4.10) | –4.76 (–11.28, 4.53) |
|  | ERR_H_/Gy | 0.13 (–0.03, 0.33) | 0.13 (–0.03, 0.34) | 0.11 (–0.04, 0.32) | 0.11 (–0.06, 0.32) |
|  | *p* value ^a^ | 0.16 | 0.21 | 0.361 | 0.268 |
| 0.010 | ERR_L_/Gy | **–2.32 (–4.02, –0.11)** | –2.24 (–3.99, 0.01) | –2.09 (–4.10, 0.54) | –1.91 (–4.42, 1.43) |
|  | ERR_H_/Gy | 0.13 (–0.03, 0.34) | 0.13 (–0.02, 0.34) | 0.12 (–0.04, 0.32) | 0.11 (–0.05, 0.32) |
|  | *p* value ^a^ | **0.031** | **0.04** | 0.092 | 0.209 |
| 0.015 | ERR_L_/Gy | **–1.73 (–2.92, –0.18)** | **–1.67 (–2.89, –0.08)** | –1.45 (–2.85, 0.37) | –1.38 (–2.99, 0.77) |
|  | ERR_H_/Gy | 0.14 (–0.02, 0.35) | 0.14 (–0.02, 0.35) | 0.13 (–0.03, 0.33) | 0.12 (–0.05, 0.33) |
|  | *p* value ^a^ | **0.021** | **0.028** | 0.083 | 0.154 |
| 0.020 | ERR_L_/Gy | **–1.45 (–2.38, –0.24)** | **–1.36 (–2.32, –0.11)** | –1.24 (–2.32, 0.16) | –1.24 (–2.46, 0.39) |
|  | ERR_H_/Gy | 0.15 (–0.01, 0.36) | 0.15 (–0.01, 0.36) | 0.13 (–0.03, 0.34) | 0.12 (–0.04, 0.34) |
|  | *p* value ^a^ | **0.013** | **0.021** | 0.055 | 0.094 |
| 0.025 | ERR_L_/Gy | **–1.19 (–1.98, –0.15)** | **–1.09 (–1.92, –0.01)** | –0.99 (–1.90, 0.23) | –1.12 (–2.11, 0.23) |
|  | ERR_H_/Gy | 0.15 (–0.01, 0.36) | 0.15 (–0.01, 0.36) | 0.13 (–0.03, 0.34) | 0.12 (–0.04, 0.34) |
|  | *p* value ^a^ | **0.015** | **0.028** | 0.068 | 0.067 |
| 0.030 | ERR_L_/Gy | –0.78 (–1.54, 0.22) | –0.70 (–1.48, 0.34) | –0.56 (–1.43, 0.59) | –0.69 (–1.62, 0.57) |
|  | ERR_H_/Gy | 0.14 (–0.02, 0.36) | 0.14 (–0.02, 0.36) | 0.13 (–0.03, 0.34) | 0.12 (–0.04, 0.34) |
|  | *p* value ^a^ | 0.067 | 0.102 | 0.217 | 0.186 |
| 0.035 | ERR_L_/Gy | –0.69 (–1.39, 0.24) | –0.62 (–1.34, 0.33) | –0.48 (–1.28, 0.58) | –0.59 (–1.45, 0.57) |
|  | ERR_H_/Gy | 0.15 (–0.02, 0.36) | 0.15 (–0.02, 0.36) | 0.13 (–0.03, 0.34) | 0.12 (–0.04, 0.34) |
|  | *p* value ^a^ | 0.073 | 0.105 | 0.234 | 0.202 |
| 0.040 | ERR_L_/Gy | –0.53 (–1.21, 0.35) | –0.47 (–1.17, 0.44) | –0.33 (–1.10, 0.67) | –0.47 (–1.29, 0.62) |
|  | ERR_H_/Gy | 0.15 (–0.01, 0.37) | 0.15 (–0.01, 0.37) | 0.13 (–0.03, 0.35) | 0.13 (–0.04, 0.35) |
|  | *p* value ^a^ | 0.119 | 0.164 | 0.331 | 0.255 |
| 0.045 | ERR_L_/Gy | –0.37 (–1.04, 0.50) | –0.33 (–1.01, 0.56) | –0.18 (–0.93, 0.79) | –0.33 (–1.13, 0.73) |
|  | ERR_H_/Gy | 0.15 (–0.02, 0.37) | 0.15 (–0.02, 0.37) | 0.13 (–0.03, 0.34) | 0.13 (–0.04, 0.35) |
|  | *p* value ^a^ | 0.221 | 0.267 | 0.496 | 0.363 |
| 0.050 | ERR_L_/Gy | –0.33 (–0.97, 0.51) | –0.28 (–0.93, 0.58) | –0.16 (–0.88, 0.77) | –0.31 (–1.07, 0.68) |
|  | ERR_H_/Gy | 0.15 (–0.02, 0.36) | 0.15 (–0.02, 0.36) | 0.13 (–0.03, 0.34) | 0.13 (–0.04, 0.35) |
|  | *p* value ^a^ | 0.243 | 0.303 | > 0.50 | 0.354 |
| *Notes:*  Numbers in bold indicate significant differences. The dataset for the analysis was stratified by sex, attained age, calendar period, smoking status, alcohol consumption, alpha dose.  CI, confidence interval. ERR/Gy, excess relative risk per unit gray of gamma-ray dose. IHD, ischemic heart disease (ICD-9 codes: 410–414). n/a denotes that it was not possible to estimate the lower or upper bound of the confidence interval.  ^a^ Likelihood ratio test comparing the models with and without cutpoint. | | | | | |

Table S5 Excess relative risk per Gy of IHD mortality in relation to 10-year lagged cumulative liver absorbed gamma-ray doses from external exposure (sensitivity analyses – various parameters of the adjustment for alpha and neutron dose, both sexes, entire cohort)

| Cutpoint,  Gy/year | Model  parameters | Sensitivity analysis type | | |
| --- | --- | --- | --- | --- |
|  |  | Exclusion of the adjustment for liver absorbed alpha dose | The alternative adjustment for liver absorbed alpha dose ^a^ | Association with the weighted sum of liver absorbed gamma-ray + neutron dose (Gy) ^b^ |
| 0 (without cutpoint) | ERR/Gy | 0.04 (–0.01, 0.11) | 0.05 (–0.01, 0.13) | 0.04 (–0.02, 0.11) |
| 0.005 | ERR_L_/Gy | **–4.59 (–6.39, –2.53)** | **–5.10 (–6.92, –2.99)** | **–4.60 (–6.49, –2.42)** |
|  | ERR_H_/Gy | 0.04 (–0.02, 0.10) | 0.04 (–0.02, 0.12) | 0.03 (–0.03, 0.10) |
|  | *p* value ^c^ | **< 0.001** | **< 0.001** | **< 0.001** |
| 0.010 | ERR_L_/Gy | **–2.61 (–3.42, –1.69)** | **–2.82 (–3.65, –1.87)** | **–2.73 (–3.55, –1.79)** |
|  | ERR_H_/Gy | 0.04 (–0.01, 0.10) | 0.05 (–0.01, 0.13) | 0.04 (–0.02, 0.11) |
|  | *p* value ^c^ | **< 0.001** | **< 0.001** | **< 0.001** |
| 0.015 | ERR_L_/Gy | **–1.81 (–2.37, –1.18)** | **–1.89 (–2.47, –1.24)** | **–1.96 (–2.52, –1.34)** |
|  | ERR_H_/Gy | 0.05 (–0.01, 0.11) | 0.06 (–0.00, 0.13) | 0.04 (–0.01, 0.11) |
|  | *p* value ^c^ | **< 0.001** | **< 0.001** | **< 0.001** |
| 0.020 | ERR_L_/Gy | **–1.21 (–1.64, –0.71)** | **–1.22 (–1.68, –0.69)** | **–1.35 (–1.78, –0.86)** |
|  | ERR_H_/Gy | 0.05 (–0.00, 0.11) | **0.07 (+0.00, 0.14)** | 0.05 (–0.01, 0.13) |
|  | *p* value ^c^ | **< 0.001** | **< 0.001** | **< 0.001** |
| 0.025 | ERR_L_/Gy | **–0.82 (–1.19, –0.41)** | **–0.81 (–1.21, –0.35)** | **–0.95 (–1.32, –0.54)** |
|  | ERR_H_/Gy | 0.06 (–0.00, 0.12) | **0.07 (+0.00, 0.15)** | 0.06 (–0.00, 0.13) |
|  | *p* value ^c^ | **< 0.001** | **< 0.001** | **< 0.001** |
| 0.030 | ERR_L_/Gy | **–0.57 (–0.89, –0.21)** | **–0.52 (–0.88, –0.11)** | **–0.71 (–1.03, –0.34)** |
|  | ERR_H_/Gy | 0.06 (–0.00, 0.12) | **0.07 (+0.00, 0.15)** | 0.06 (–0.00, 0.14) |
|  | *p* value ^c^ | **0.001** | **0.007** | **< 0.001** |
| 0.035 | ERR_L_/Gy | **–0.38 (–0.67, –0.05)** | –0.32 (–0.64, 0.05) | **–0.50 (–0.79, –0.17)** |
|  | ERR_H_/Gy | 0.06 (–0.00, 0.12) | 0.07 (–0.00, 0.15) | 0.06 (–0.00, 0.14) |
|  | *p* value ^c^ | **0.011** | **0.047** | **0.002** |
| 0.040 | ERR_L_/Gy | –0.27 (–0.52, 0.02) | –0.20 (–0.49, 0.14) | **–0.36 (–0.62, –0.07)** |
|  | ERR_H_/Gy | 0.06 (–0.00, 0.12) | 0.07 (–0.00, 0.15) | 0.06 (–0.00, 0.14) |
|  | *p* value ^c^ | **0.034** | 0.133 | **0.008** |
| 0.045 | ERR_L_/Gy | –0.17 (–0.41, 0.10) | –0.08 (–0.36, 0.23) | –0.27 (–0.51, +0.00) |
|  | ERR_H_/Gy | 0.05 (–0.00, 0.12) | 0.06 (–0.01, 0.14) | 0.06 (–0.01, 0.14) |
|  | *p* value ^c^ | 0.103 | 0.357 | **0.023** |
| 0.050 | ERR_L_/Gy | –0.12 (–0.34, 0.13) | –0.02 (–0.28, 0.28) | –0.19 (–0.41, 0.07) |
|  | ERR_H_/Gy | 0.05 (–0.01, 0.12) | 0.06 (–0.01, 0.14) | 0.06 (–0.01, 0.13) |
|  | *p* value ^c^ | 0.187 | > 0.50 | 0.07 |
| *Notes:*  Numbers in bold indicate significant differences.  CI, confidence interval. ERR/Gy, excess relative risk per unit gray of gamma-ray dose. IHD, ischemic heart disease (ICD-9 codes: 410–414). n/a denotes that it was not possible to estimate the lower bound or upper of the confidence interval.  ^a^ Unmonitored for plutonium alpha activity workers divided into two subgroups: only workers of reactors and the rest of unmonitored workers.  ^b^ For all workers.  ^c^ Likelihood ratio test comparing the models with and without cutpoint. | | | | |

Table S6 Excess relative risk per Gy of IHD mortality in relation to 10-year lagged cumulative liver absorbed gamma-ray doses from external exposure (sensitivity analyses – various parameters of the adjustment for alpha and neutron dose, males, entire cohort)

| Cutpoint,  Gy/year | Model  parameters | Sensitivity analysis type | | |
| --- | --- | --- | --- | --- |
|  |  | Exclusion of the adjustment for liver absorbed alpha dose | The alternative adjustment for liver absorbed alpha dose ^a^ | Association with the weighted sum of liver absorbed gamma-ray + neutron dose (Gy) ^b^ |
| 0 (without cutpoint) | ERR/Gy | 0.05 (–0.01, 0.12) | 0.04 (–0.03, 0.12) | 0.02 (–0.05, 0.10) |
| 0.005 | ERR_L_/Gy | **–5.08 (–6.99, –2.84)** | **–5.60 (–7.50, –3.34)** | **–4.94 (–6.96, –2.56)** |
|  | ERR_H_/Gy | 0.04 (–0.02, 0.11) | 0.03 (–0.04, 0.11) | 0.01 (–0.05, 0.08) |
|  | *p* value ^c^ | **< 0.001** | **< 0.001** | **< 0.001** |
| 0.010 | ERR_L_/Gy | **–2.81 (–3.70, –1.79)** | **–3.04 (–3.93, –2.01)** | **–2.90 (–3.79, –1.87)** |
|  | ERR_H_/Gy | 0.05 (–0.01, 0.12) | 0.04 (–0.03, 0.12) | 0.02 (–0.04, 0.09) |
|  | *p* value ^c^ | **< 0.001** | **< 0.001** | **< 0.001** |
| 0.015 | ERR_L_/Gy | **–1.94 (–2.55, –1.25)** | **–2.03 (–2.64, –1.32)** | **–2.05 (–2.64, –1.37)** |
|  | ERR_H_/Gy | 0.06 (–0.00, 0.13) | 0.04 (–0.02, 0.12) | 0.03 (–0.04, 0.10) |
|  | *p* value ^c^ | **< 0.001** | **< 0.001** | **< 0.001** |
| 0.020 | ERR_L_/Gy | **–1.24 (–1.72, –0.69)** | **–1.24 (–1.74, –0.66)** | **–1.36 (–1.82, –0.82)** |
|  | ERR_H_/Gy | **0.06 (+0.00, 0.13)** | 0.05 (–0.02, 0.13) | 0.04 (–0.03, 0.11) |
|  | *p* value ^c^ | **< 0.001** | **< 0.001** | **< 0.001** |
| 0.025 | ERR_L_/Gy | **–0.81 (–1.22, –0.35)** | **–0.78 (–1.22, –0.27)** | **–0.93 (–1.33, –0.47)** |
|  | ERR_H_/Gy | **0.07 (+0.00, 0.14)** | 0.06 (–0.02, 0.14) | 0.04 (–0.02, 0.12) |
|  | *p* value ^c^ | **< 0.001** | **0.002** | **< 0.001** |
| 0.030 | ERR_L_/Gy | **–0.57 (–0.92, –0.17)** | **–0.52 (–0.91, –0.07)** | **–0.71 (–1.05, –0.32)** |
|  | ERR_H_/Gy | **0.07 (+0.00, 0.14)** | 0.06 (–0.02, 0.14) | 0.05 (–0.02, 0.13) |
|  | *p* value ^c^ | **0.003** | **0.015** | **< 0.001** |
| 0.035 | ERR_L_/Gy | **–0.36 (–0.67, –0.00)** | –0.29 (–0.64, 0.12) | **–0.48 (–0.79, –0.13)** |
|  | ERR_H_/Gy | **0.07 (+0.00, 0.14)** | 0.05 (–0.02, 0.14) | 0.05 (–0.02, 0.13) |
|  | *p* value ^c^ | **0.023** | 0.108 | **0.006** |
| 0.040 | ERR_L_/Gy | –0.25 (–0.52, 0.07) | –0.17 (–0.49, 0.20) | **–0.35 (–0.62, –0.03)** |
|  | ERR_H_/Gy | 0.06 (–0.00, 0.14) | 0.05 (–0.02, 0.14) | 0.04 (–0.03, 0.12) |
|  | *p* value ^c^ | 0.057 | 0.24 | **0.021** |
| 0.045 | ERR_L_/Gy | –0.16 (–0.41, 0.13) | –0.07 (–0.36, 0.27) | –0.26 (–0.51, 0.03) |
|  | ERR_H_/Gy | 0.06 (–0.00, 0.14) | 0.04 (–0.03, 0.13) | 0.04 (–0.03, 0.12) |
|  | *p* value ^c^ | 0.141 | > 0.50 | 0.051 |
| 0.050 | ERR_L_/Gy | –0.10 (–0.34, 0.17) | –0.00 (–0.28, 0.32) | –0.18 (–0.41, 0.09) |
|  | ERR_H_/Gy | 0.06 (–0.01, 0.14) | 0.04 (–0.03, 0.13) | 0.04 (–0.03, 0.12) |
|  | *p* value ^c^ | 0.242 | > 0.50 | 0.13 |
| *Notes:*  Numbers in bold indicate significant differences.  CI, confidence interval. ERR/Gy, excess relative risk per unit gray of gamma-ray dose. IHD, ischemic heart disease (ICD-9 codes: 410–414). n/a denotes that it was not possible to estimate the lower bound or upper of the confidence interval.  ^a^ Unmonitored for plutonium alpha activity workers divided into two subgroups: only workers of reactors and the rest of unmonitored workers.  ^b^ For all workers.  ^c^ Likelihood ratio test comparing the models with and without cutpoint. | | | | |

Table S7 Excess relative risk per Gy of IHD mortality in relation to 10-year lagged cumulative liver absorbed gamma-ray doses from external exposure (sensitivity analyses – various parameters of the adjustment for alpha and neutron dose, females, entire cohort)

| Cutpoint,  Gy/year | Model  parameters | Sensitivity analysis type | | |
| --- | --- | --- | --- | --- |
|  |  | Exclusion of the adjustment for liver absorbed alpha dose | The alternative adjustment for liver absorbed alpha dose ^a^ | Association with the weighted sum of liver absorbed gamma-ray + neutron dose (Gy) ^b^ |
| 0 (without cutpoint) | ERR/Gy | 0.01 (–0.10, 0.16) | 0.13 (–0.04, 0.35) | 0.14 (–0.03, 0.36) |
| 0.005 | ERR_L_/Gy | –2.46 (–6.74, 3.11) | –2.72 (–7.23, 3.30) | –3.03 (–7.44, 2.84) |
|  | ERR_H_/Gy | 0.01 (–0.10, 0.15) | 0.13 (–0.04, 0.35) | 0.13 (–0.03, 0.35) |
|  | *p* value ^c^ | 0.348 | 0.315 | 0.258 |
| 0.010 | ERR_L_/Gy | –1.93 (–3.67, 0.27) | –1.92 (–3.81, 0.54) | –2.03 (–3.86, 0.35) |
|  | ERR_H_/Gy | 0.01 (–0.10, 0.14) | 0.13 (–0.03, 0.35) | 0.14 (–0.02, 0.35) |
|  | *p* value ^c^ | 0.079 | 0.094 | 0.07 |
| 0.015 | ERR_L_/Gy | –1.31 (–2.53, 0.25) | –1.29 (–2.66, 0.48) | –1.58 (–2.86, 0.06) |
|  | ERR_H_/Gy | 0.01 (–0.10, 0.15) | 0.14 (–0.03, 0.36) | 0.15 (–0.01, 0.36) |
|  | *p* value ^c^ | 0.089 | 0.105 | **0.04** |
| 0.020 | ERR_L_/Gy | –1.07 (–2.04, 0.17) | –1.09 (–2.16, 0.32) | –1.25 (–2.25, 0.06) |
|  | ERR_H_/Gy | 0.02 (–0.10, 0.16) | 0.14 (–0.02, 0.36) | 0.15 (–0.01, 0.37) |
|  | *p* value ^c^ | 0.081 | 0.082 | **0.037** |
| 0.025 | ERR_L_/Gy | –0.92 (–1.74, 0.14) | –0.90 (–1.81, 0.31) | –0.99 (–1.85, 0.14) |
|  | ERR_H_/Gy | 0.02 (–0.10, 0.16) | 0.14 (–0.02, 0.36) | 0.15 (–0.01, 0.37) |
|  | *p* value ^c^ | 0.08 | 0.084 | **0.047** |
| 0.030 | ERR_L_/Gy | –0.60 (–1.37, 0.39) | –0.49 (–1.36, 0.66) | –0.62 (–1.44, 0.46) |
|  | ERR_H_/Gy | 0.02 (–0.10, 0.16) | 0.14 (–0.03, 0.36) | 0.15 (–0.02, 0.37) |
|  | *p* value ^c^ | 0.202 | 0.256 | 0.145 |
| 0.035 | ERR_L_/Gy | –0.52 (–1.23, 0.39) | –0.45 (–1.25, 0.60) | –0.53 (–1.29, 0.45) |
|  | ERR_H_/Gy | 0.02 (–0.10, 0.16) | 0.14 (–0.03, 0.37) | 0.15 (–0.01, 0.37) |
|  | *p* value ^c^ | 0.222 | 0.242 | 0.156 |
| 0.040 | ERR_L_/Gy | –0.42 (–1.09, 0.44) | –0.31 (–1.07, 0.69) | –0.40 (–1.13, 0.54) |
|  | ERR_H_/Gy | 0.02 (–0.09, 0.17) | 0.14 (–0.03, 0.37) | 0.16 (–0.01, 0.38) |
|  | *p* value ^c^ | 0.29 | 0.34 | 0.221 |
| 0.045 | ERR_L_/Gy | –0.30 (–0.95, 0.52) | –0.15 (–0.90, 0.83) | –0.28 (–0.99, 0.64) |
|  | ERR_H_/Gy | 0.02 (–0.10, 0.17) | 0.14 (–0.03, 0.37) | 0.15 (–0.01, 0.38) |
|  | *p* value ^c^ | 0.414 | > 0.50 | 0.326 |
| 0.050 | ERR_L_/Gy | –0.27 (–0.89, 0.52) | –0.11 (–0.83, 0.83) | –0.21 (–0.89, 0.68) |
|  | ERR_H_/Gy | 0.02 (–0.10, 0.17) | 0.14 (–0.03, 0.36) | 0.15 (–0.02, 0.37) |
|  | *p* value ^c^ | 0.448 | > 0.50 | 0.391 |
| *Notes:*  Numbers in bold indicate significant differences.  CI, confidence interval. ERR/Gy, excess relative risk per unit gray of gamma-ray dose. IHD, ischemic heart disease (ICD-9 codes: 410–414). n/a denotes that it was not possible to estimate the lower bound or upper of the confidence interval.  ^a^ Unmonitored for plutonium alpha activity workers divided into two subgroups: only workers of reactors and the rest of unmonitored workers.  ^b^ For all workers.  ^c^ Likelihood ratio test comparing the models with and without cutpoint. | | | | |

Table S8 Excess relative risk per Gy of IHD mortality in relation to 10-year lagged cumulative liver absorbed gamma-ray doses from external exposure (sensitivity analyses – dataset restricted and additional inclusion of the adjustment, both sexes, entire cohort)

| Cutpoint,  Gy/year | Model  parameters | Sensitivity analysis type | | |
| --- | --- | --- | --- | --- |
|  |  | Dataset restricted to workers employed for > 1 year | Inclusion of the adjustment for period of hire | Inclusion of the adjustment for age at hire |
| 0 (without cutpoint) | ERR/Gy | 0.05 (–0.02, 0.12) | 0.04 (–0.02, 0.12) | 0.07 (–0.00, 0.16) |
| 0.005 | ERR_L_/Gy | **–4.87 (–6.73, –2.70)** | **–4.30 (–6.28, –2.01)** | **–4.36 (–6.41, –1.97)** |
|  | ERR_H_/Gy | 0.04 (–0.02, 0.11) | 0.03 (–0.03, 0.11) | 0.06 (–0.01, 0.14) |
|  | *p* value ^a^ | **< 0.001** | **< 0.001** | **< 0.001** |
| 0.010 | ERR_L_/Gy | **–2.86 (–3.69, –1.93)** | **–2.83 (–3.66, –1.88)** | **–2.82 (–3.69, –1.83)** |
|  | ERR_H_/Gy | 0.05 (–0.01, 0.12) | 0.04 (–0.02, 0.11) | 0.07 (–0.00, 0.15) |
|  | *p* value ^a^ | **< 0.001** | **< 0.001** | **< 0.001** |
| 0.015 | ERR_L_/Gy | **–2.04 (–2.59, –1.41)** | **–2.07 (–2.62, –1.44)** | **–2.11 (–2.67, –1.45)** |
|  | ERR_H_/Gy | 0.06 (–0.01, 0.13) | 0.05 (–0.02, 0.12) | **0.07 (+0.00, 0.16)** |
|  | *p* value ^a^ | **< 0.001** | **< 0.001** | **< 0.001** |
| 0.020 | ERR_L_/Gy | **–1.41 (–1.84, –0.91)** | **–1.48 (–1.91, –0.99)** | **–1.54 (–1.97, –1.04)** |
|  | ERR_H_/Gy | **0.07 (+0.00, 0.14)** | 0.06 (–0.01, 0.13) | **0.09 (0.02, 0.17)** |
|  | *p* value ^a^ | **< 0.001** | **< 0.001** | **< 0.001** |
| 0.025 | ERR_L_/Gy | **–1.01 (–1.38, –0.60)** | **–1.12 (–1.47, –0.71)** | **–1.15 (–1.51, –0.72)** |
|  | ERR_H_/Gy | **0.07 (0.01, 0.15)** | 0.06 (–0.00, 0.14) | **0.10 (0.02, 0.18)** |
|  | *p* value ^a^ | **< 0.001** | **< 0.001** | **< 0.001** |
| 0.030 | ERR_L_/Gy | **–0.72 (–1.05, –0.35)** | **–0.83 (–1.15, –0.47)** | **–0.88 (–1.20, –0.51)** |
|  | ERR_H_/Gy | **0.08 (0.01, 0.15)** | 0.07 (–0.00, 0.14) | **0.10 (0.03, 0.19)** |
|  | *p* value ^a^ | **< 0.001** | **< 0.001** | **< 0.001** |
| 0.035 | ERR_L_/Gy | **–0.50 (–0.79, –0.17)** | **–0.62 (–0.90, –0.29)** | **–0.67 (–0.96, –0.34)** |
|  | ERR_H_/Gy | **0.08 (0.01, 0.15)** | **0.07 (+0.00, 0.15)** | **0.11 (0.03, 0.19)** |
|  | *p* value ^a^ | **0.001** | **< 0.001** | **< 0.001** |
| 0.040 | ERR_L_/Gy | **–0.37 (–0.63, –0.07)** | **–0.48 (–0.73, –0.19)** | **–0.53 (–0.79, –0.24)** |
|  | ERR_H_/Gy | **0.07 (0.01, 0.15)** | **0.07 (+0.00, 0.15)** | **0.11 (0.03, 0.20)** |
|  | *p* value ^a^ | **0.006** | **< 0.001** | **< 0.001** |
| 0.045 | ERR_L_/Gy | –0.25 (–0.49, 0.03) | **–0.35 (–0.59, –0.08)** | **–0.41 (–0.64, –0.14)** |
|  | ERR_H_/Gy | **0.07 (+0.00, 0.15)** | 0.07 (–0.00, 0.15) | **0.11 (0.03, 0.20)** |
|  | *p* value ^a^ | **0.032** | **0.004** | **< 0.001** |
| 0.050 | ERR_L_/Gy | –0.18 (–0.41, 0.09) | **–0.28 (–0.50, –0.02)** | **–0.33 (–0.55, –0.07)** |
|  | ERR_H_/Gy | 0.07 (–0.00, 0.15) | 0.07 (–0.00, 0.15) | **0.11 (0.03, 0.20)** |
|  | *p* value ^a^ | 0.078 | **0.013** | **0.002** |
| *Notes:*  Numbers in bold indicate significant differences.  CI, confidence interval. ERR/Gy, excess relative risk per unit gray of gamma-ray dose. IHD, ischemic heart disease (ICD-9 codes: 410–414). n/a denotes that it was not possible to estimate the lower or upper bound of the confidence interval.  ^a^ Likelihood ratio test comparing the models with and without cutpoint. | | | | |

Table S9 Excess relative risk per Gy of IHD mortality in relation to 10-year lagged cumulative liver absorbed gamma-ray doses from external exposure (sensitivity analyses – dataset restricted and additional inclusion of the adjustment, males, entire cohort)

| Cutpoint,  Gy/year | Model  parameters | Sensitivity analysis type | | |
| --- | --- | --- | --- | --- |
|  |  | Dataset restricted to workers employed for >1 year | Inclusion of the adjustment for period of hire | Inclusion of the adjustment for age at hire |
| 0 (without cutpoint) | ERR/Gy | 0.03 (–0.04, 0.11) | 0.02 (–0.05, 0.10) | 0.03 (–0.04, 0.12) |
| 0.005 | ERR_L_/Gy | **–5.29 (–7.25, –2.95)** | **–4.83 (–6.92, –2.37)** | **–4.81 (–6.98, –2.22)** |
|  | ERR_H_/Gy | 0.02 (–0.04, 0.10) | 0.01 (–0.05, 0.09) | 0.03 (–0.04, 0.11) |
|  | *p* value ^a^ | **< 0.001** | **< 0.001** | **< 0.001** |
| 0.010 | ERR_L_/Gy | **–3.04 (–3.93, –2.01)** | **–3.03 (–3.92, –1.99)** | **–3.00 (–3.93, –1.91)** |
|  | ERR_H_/Gy | 0.03 (–0.03, 0.11) | 0.02 (–0.04, 0.10) | 0.03 (–0.04, 0.11) |
|  | *p* value ^a^ | **< 0.001** | **< 0.001** | **< 0.001** |
| 0.015 | ERR_L_/Gy | **–2.15 (–2.74, –1.46)** | **–2.20 (–2.78, –1.52)** | **–2.25 (–2.84, –1.55)** |
|  | ERR_H_/Gy | 0.04 (–0.03, 0.11) | 0.03 (–0.04, 0.10) | 0.04 (–0.03, 0.12) |
|  | *p* value ^a^ | **< 0.001** | **< 0.001** | **< 0.001** |
| 0.020 | ERR_L_/Gy | **–1.42 (–1.89, –0.88)** | **–1.52 (–1.97, –0.99)** | **–1.58 (–2.04, –1.05)** |
|  | ERR_H_/Gy | 0.05 (–0.02, 0.13) | 0.04 (–0.03, 0.12) | 0.05 (–0.02, 0.14) |
|  | *p* value ^a^ | **< 0.001** | **< 0.001** | **< 0.001** |
| 0.025 | ERR_L_/Gy | **–0.99 (–1.39, –0.53)** | **–1.12 (–1.50, –0.68)** | **–1.17 (–1.55, –0.72)** |
|  | ERR_H_/Gy | 0.05 (–0.01, 0.14) | 0.04 (–0.03, 0.12) | 0.06 (–0.01, 0.15) |
|  | *p* value ^a^ | **< 0.001** | **< 0.001** | **< 0.001** |
| 0.030 | ERR_L_/Gy | **–0.72 (–1.07, –0.32)** | **–0.85 (–1.18, –0.47)** | **–0.92 (–1.25, –0.54)** |
|  | ERR_H_/Gy | 0.06 (–0.01, 0.14) | 0.05 (–0.02, 0.13) | 0.07 (–0.01, 0.16) |
|  | *p* value ^a^ | **< 0.001** | **< 0.001** | **< 0.001** |
| 0.035 | ERR_L_/Gy | **–0.48 (–0.79, –0.11)** | **–0.61 (–0.91, –0.27)** | **–0.69 (–0.98, –0.34)** |
|  | ERR_H_/Gy | 0.06 (–0.02, 0.14) | 0.05 (–0.02, 0.13) | 0.07 (–0.01, 0.16) |
|  | *p* value ^a^ | **0.007** | **< 0.001** | **< 0.001** |
| 0.040 | ERR_L_/Gy | **–0.35 (–0.63, –0.03)** | **–0.48 (–0.75, –0.17)** | **–0.55 (–0.81, –0.25)** |
|  | ERR_H_/Gy | 0.05 (–0.02, 0.14) | 0.05 (–0.02, 0.13) | 0.07 (–0.01, 0.17) |
|  | *p* value ^a^ | **0.02** | **0.002** | **< 0.001** |
| 0.045 | ERR_L_/Gy | –0.23 (–0.49, 0.06) | **–0.36 (–0.60, –0.07)** | **–0.44 (–0.67, –0.16)** |
|  | ERR_H_/Gy | 0.05 (–0.02, 0.14) | 0.05 (–0.03, 0.13) | 0.07 (–0.01, 0.17) |
|  | *p* value ^a^ | 0.07 | **0.009** | **0.001** |
| 0.050 | ERR_L_/Gy | –0.16 (–0.40, 0.12) | **–0.28 (–0.51, –0.01)** | **–0.35 (–0.58, –0.09)** |
|  | ERR_H_/Gy | 0.05 (–0.03, 0.13) | 0.04 (–0.03, 0.13) | 0.07 (–0.01, 0.17) |
|  | *p* value ^a^ | 0.156 | **0.025** | **0.004** |
| *Notes:*  Numbers in bold indicate significant differences.  CI, confidence interval. ERR/Gy, excess relative risk per unit gray of gamma-ray dose. IHD, ischemic heart disease (ICD-9 codes: 410–414). n/a denotes that it was not possible to estimate the lower or upper bound of the confidence interval.  ^a^ Likelihood ratio test comparing the models with and without cutpoint. | | | | |

Table S10 Excess relative risk per Gy of IHD mortality in relation to 10-year lagged cumulative liver absorbed gamma-ray doses from external exposure (sensitivity analyses – dataset restricted and additional inclusion of the adjustment, females, entire cohort)

| Cutpoint,  Gy/year | Model  parameters | Sensitivity analysis type | | |
| --- | --- | --- | --- | --- |
|  |  | Dataset restricted to workers employed for >1 year | Inclusion of the adjustment for period of hire | Inclusion of the adjustment for age at hire |
| 0 (without cutpoint) | ERR/Gy | 0.15 (–0.02, 0.38) | 0.17 (–0.01, 0.42) | **0.32 (0.09, 0.65)** |
| 0.005 | ERR_L_/Gy | –2.89 (–7.40, 3.19) | –1.94 (–6.72, 4.46) | –2.43 (–7.26, 4.17) |
|  | ERR_H_/Gy | 0.15 (–0.02, 0.37) | 0.17 (–0.01, 0.41) | **0.32 (0.08, 0.64)** |
|  | *p* value ^a^ | 0.29 | 0.475 | 0.371 |
| 0.010 | ERR_L_/Gy | –2.14 (–3.98, 0.28) | –2.01 (–3.89, 0.46) | –2.05 (–4.03, 0.59) |
|  | ERR_H_/Gy | 0.15 (–0.02, 0.36) | 0.17 (–0.01, 0.40) | **0.32 (0.09, 0.63)** |
|  | *p* value ^a^ | 0.061 | 0.078 | 0.073 |
| 0.015 | ERR_L_/Gy | –1.56 (–2.85, 0.13) | –1.48 (–2.81, 0.26) | –1.44 (–2.87, 0.47) |
|  | ERR_H_/Gy | 0.16 (–0.01, 0.38) | 0.18 (–0.00, 0.42) | **0.33 (0.10, 0.65)** |
|  | *p* value ^a^ | **0.046** | 0.06 | 0.065 |
| 0.020 | ERR_L_/Gy | –1.28 (–2.29, 0.06) | –1.22 (–2.27, 0.18) | –1.21 (–2.34, 0.33) |
|  | ERR_H_/Gy | 0.16 (–0.01, 0.39) | **0.18 (+0.00, 0.42)** | **0.34 (0.10, 0.66)** |
|  | *p* value ^a^ | **0.036** | **0.05** | **0.047** |
| 0.025 | ERR_L_/Gy | –1.08 (–1.93, 0.06) | –1.01 (–1.90, 0.19) | –0.87 (–1.87, 0.51) |
|  | ERR_H_/Gy | 0.16 (–0.01, 0.39) | 0.18 (–0.00, 0.42) | **0.34 (0.10, 0.66)** |
|  | *p* value ^a^ | **0.034** | 0.051 | 0.078 |
| 0.030 | ERR_L_/Gy | –0.64 (–1.46, 0.46) | –0.62 (–1.47, 0.53) | –0.39 (–1.37, 0.95) |
|  | ERR_H_/Gy | 0.16 (–0.01, 0.39) | 0.18 (–0.00, 0.42) | **0.33 (0.10, 0.66)** |
|  | *p* value ^a^ | 0.138 | 0.155 | 0.254 |
| 0.035 | ERR_L_/Gy | –0.61 (–1.35, 0.39) | –0.54 (–1.34, 0.52) | –0.34 (–1.25, 0.90) |
|  | ERR_H_/Gy | 0.16 (–0.01, 0.39) | 0.18 (–0.00, 0.42) | **0.34 (0.10, 0.66)** |
|  | *p* value ^a^ | 0.115 | 0.163 | 0.251 |
| 0.040 | ERR_L_/Gy | –0.44 (–1.16, 0.51) | –0.38 (–1.15, 0.64) | –0.16 (–1.04, 1.03) |
|  | ERR_H_/Gy | 0.17 (–0.01, 0.40) | 0.18 (–0.00, 0.43) | **0.34 (0.10, 0.67)** |
|  | *p* value ^a^ | 0.19 | 0.249 | 0.367 |
| 0.045 | ERR_L_/Gy | –0.28 (–0.99, 0.66) | –0.23 (–0.99, 0.77) | 0.05 (–0.84, 1.23) |
|  | ERR_H_/Gy | 0.17 (–0.01, 0.40) | 0.18 (–0.00, 0.43) | **0.33 (0.10, 0.67)** |
|  | *p* value ^a^ | 0.318 | 0.379 | > 0.50 |
| 0.050 | ERR_L_/Gy | –0.25 (–0.93, 0.65) | –0.18 (–0.91, 0.79) | 0.08 (–0.77, 1.22) |
|  | ERR_H_/Gy | 0.16 (–0.01, 0.39) | 0.18 (–0.00, 0.42) | **0.33 (0.09, 0.66)** |
|  | *p* value ^a^ | 0.337 | 0.423 | > 0.50 |
| *Notes:*  Numbers in bold indicate significant differences.  CI, confidence interval. ERR/Gy, excess relative risk per unit gray of gamma-ray dose. IHD, ischemic heart disease (ICD-9 codes: 410–414). n/a denotes that it was not possible to estimate the lower or upper bound of the confidence interval.  ^a^ Likelihood ratio test comparing the models with and without cutpoint. | | | | |

Table S11 Excess relative risk per Gy of IHD mortality in relation to 10-year lagged cumulative liver absorbed gamma-ray doses from external exposure (non-linear analysis, both sexes, entire cohort)

| Cutpoint,  Gy/year | Model  parameters | Models ^a^ | | | |
| --- | --- | --- | --- | --- | --- |
|  |  | (1) | (2) ^b^ | (3) ^b^ | (4) ^b^ |
| 0.005 | β_L1_ | **–4.71 (–6.58, –2.56)** | –5.22 (–10.67, 0.24) | **–4.71 (–6.73, –2.69)** | –5.21 (–10.66, 0.24) |
|  | β_H1_ | 0.03 (–0.03, 0.10) | 0.03 (–0.03, 0.09) | 0.02 (–0.10, 0.15) | 0.02 (–0.10, 0.15) |
|  | β_L2_ | – | 8.68 (–78.51, 95.87) | – | 8.55 (–78.53, 95.63) |
|  | β_H2_ | – | – | +0.00 (–0.04, 0.05) | +0.00 (–0.04, 0.05) |
|  | *p* value ^c^ | – | > 0.50 | > 0.50 | > 0.50 |
| 0.010 | β_L1_ | **–2.77 (–3.59, –1.84)** | –1.44 (–4.03, 1.15) | **–2.78 (–3.64, –1.92)** | –1.44 (–4.04, 1.15) |
|  | β_H1_ | 0.04 (–0.02, 0.11) | 0.04 (–0.02, 0.11) | 0.05 (–0.07, 0.18) | 0.06 (–0.07, 0.18) |
|  | β_L2_ | – | –11.13 (–30.76, 8.49) | – | –11.17 (–30.86, 8.51) |
|  | β_H2_ | – | – | –0.01 (–0.05, 0.04) | –0.01 (–0.05, 0.04) |
|  | *p* value ^c^ | – | 0.277 | > 0.50 | > 0.50 |
| 0.015 | β_L1_ | **–1.98 (–2.53, –1.36)** | –1.76 (–3.52, 0.01) | **–2.00 (–2.59, –1.41)** | –1.77 (–3.55, +0.00) |
|  | β_H1_ | 0.05 (–0.01, 0.11) | 0.05 (–0.02, 0.11) | 0.08 (–0.04, 0.20) | 0.08 (–0.05, 0.20) |
|  | β_L2_ | – | –1.31 (–10.89, 8.27) | – | –1.34 (–10.97, 8.30) |
|  | β_H2_ | – | – | –0.01 (–0.05, 0.03) | –0.01 (–0.06, 0.03) |
|  | *p* value ^c^ | – | > 0.50 | > 0.50 | > 0.50 |
| 0.020 | β_L1_ | **–1.37 (–1.80, –0.88)** | **–2.03 (–3.35, –0.71)** | **–1.40 (–1.88, –0.92)** | **–2.06 (–3.38, –0.73)** |
|  | β_H1_ | 0.06 (–0.01, 0.13) | 0.06 (–0.01, 0.12) | 0.11 (–0.02, 0.23) | 0.10 (–0.02, 0.23) |
|  | β_L2_ | – | 2.89 (–2.72, 8.51) | – | 2.88 (–2.77, 8.53) |
|  | β_H2_ | – | – | –0.02 (–0.06, 0.02) | –0.02 (–0.06, 0.02) |
|  | *p* value ^c^ | – | 0.3 | 0.381 | 0.403 |
| 0.025 | β_L1_ | **–0.98 (–1.35, –0.57)** | **–1.19 (–2.27, –0.11)** | **–1.01 (–1.41, –0.62)** | **–1.22 (–2.31, –0.13)** |
|  | β_H1_ | 0.06 (–0.00, 0.13) | 0.06 (–0.01, 0.13) | 0.12 (–0.01, 0.25) | 0.12 (–0.01, 0.25) |
|  | β_L2_ | – | 0.73 (–2.86, 4.33) | – | 0.72 (–2.90, 4.34) |
|  | β_H2_ | – | – | –0.03 (–0.07, 0.02) | –0.02 (–0.07, 0.02) |
|  | *p* value ^c^ | – | > 0.50 | 0.3 | > 0.50 |
| 0.030 | β_L1_ | **–0.69 (–1.02, –0.33)** | **–1.07 (–1.99, –0.15)** | **–0.73 (–1.08, –0.37)** | **–1.10 (–2.02, –0.17)** |
|  | β_H1_ | 0.06 (–0.00, 0.14) | 0.06 (–0.01, 0.13) | 0.13 (–0.00, 0.26) | 0.13 (–0.00, 0.25) |
|  | β_L2_ | – | 1.12 (–1.49, 3.72) | – | 1.10 (–1.51, 3.72) |
|  | β_H2_ | – | – | –0.03 (–0.07, 0.01) | –0.03 (–0.07, 0.01) |
|  | *p* value ^c^ | – | 0.394 | 0.274 | 0.388 |
| 0.035 | β_L1_ | **–0.49 (–0.78, –0.16)** | **–0.89 (–1.68, –0.11)** | **–0.52 (–0.83, –0.20)** | **–0.91 (–1.70, –0.12)** |
|  | β_H1_ | 0.06 (–0.00, 0.14) | 0.06 (–0.01, 0.13) | **0.13 (+0.00, 0.26)** | 0.13 (–0.00, 0.26) |
|  | β_L2_ | – | 1.03 (–0.85, 2.92) | – | 1.01 (–0.89, 2.91) |
|  | β_H2_ | – | – | –0.03 (–0.07, 0.01) | –0.03 (–0.07, 0.01) |
|  | *p* value ^c^ | – | 0.28 | 0.248 | 0.295 |
| 0.040 | β_L1_ | **–0.36 (–0.62, –0.07)** | **–0.88 (–1.54, –0.21)** | **–0.39 (–0.68, –0.10)** | **–0.90 (–1.57, –0.24)** |
|  | β_H1_ | 0.06 (–0.00, 0.14) | 0.06 (–0.01, 0.13) | **0.13 (+0.00, 0.27)** | 0.13 (–0.00, 0.26) |
|  | β_L2_ | – | 1.14 (–0.25, 2.53) | – | 1.14 (–0.26, 2.53) |
|  | β_H2_ | – | – | –0.03 (–0.07, 0.01) | –0.03 (–0.07, 0.01) |
|  | *p* value ^c^ | – | 0.104 | 0.243 | 0.139 |
| 0.045 | β_L1_ | –0.25 (–0.48, 0.03) | **–0.72 (–1.30, –0.13)** | **–0.27 (–0.54, –0.00)** | **–0.74 (–1.34, –0.15)** |
|  | β_H1_ | 0.06 (–0.01, 0.14) | 0.06 (–0.01, 0.13) | 0.13 (–0.00, 0.27) | 0.13 (–0.00, 0.26) |
|  | β_L2_ | – | 0.90 (–0.17, 1.97) | – | 0.91 (–0.17, 1.98) |
|  | β_H2_ | – | – | –0.03 (–0.07, 0.01) | –0.03 (–0.07, 0.01) |
|  | *p* value ^c^ | – | 0.091 | 0.24 | 0.119 |
| 0.050 | β_L1_ | –0.18 (–0.40, 0.08) | **–0.64 (–1.18, –0.09)** | –0.20 (–0.45, 0.05) | **–0.66 (–1.21, –0.11)** |
|  | β_H1_ | 0.06 (–0.01, 0.13) | 0.05 (–0.01, 0.12) | 0.13 (–0.01, 0.26) | 0.12 (–0.00, 0.25) |
|  | β_L2_ | – | 0.81 (–0.10, 1.72) | – | 0.81 (–0.10, 1.73) |
|  | β_H2_ | – | – | –0.03 (–0.07, 0.01) | –0.03 (–0.07, 0.01) |
|  | *p* value ^c^ | – | 0.081 | 0.249 | 0.11 |
| *Notes:*  Numbers in bold indicate significant differences. The dataset for the analysis was stratified by sex, attained age, calendar period, smoking status, alcohol consumption, alpha dose.  CI, confidence interval. ERR/Gy, excess relative risk per unit gray of gamma-ray dose. IHD, ischemic heart disease (ICD-9 codes: 410–414). n/a denotes that it was not possible to estimate the lower or upper bound of the confidence interval.  ^a^ Equations of the models: (1) λ = λ_0_(1 + β_L1_D_L_ + β_H1_D_H_), (2) λ = λ_0_(1 + β_L1_D_L_+ β_L2_D_L_^2^ + β_Η1_D_H_), (3) λ = λ_0_(1 + β_L1_D_L_+ β_Η1_D_H_ + β_Η2_D_H_^2^),  (4) λ = λ_0_(1 + β_L1_D_L_ + β_L2_D_L_^2^ + β_Η1_D_H_ + β_Η2_D_H_^2^)  ^b^ Wald-type confidence interval.  ^c^ *p*-value denotes significant differences from a linear model. | | | | | |

Table S12 Excess relative risk per Gy of IHD mortality in relation to 10-year lagged cumulative liver absorbed gamma-ray doses from external exposure (non-linear analysis, males, entire cohort)

| Cutpoint,  Gy/year | Model  parameters | Models ^a^ | | | |
| --- | --- | --- | --- | --- | --- |
|  |  | (1) | (2) ^b^ | (3) ^b^ | (4) ^b^ |
| 0.005 | β_L1_ | **–5.06 (–7.05, –2.72)** | –6.01 (–12.05, 0.04) | **–5.06 (–7.25, –2.88)** | –6.00 (–12.05, 0.04) |
|  | β_H1_ | 0.01 (–0.05, 0.08) | 0.01 (–0.06, 0.08) | 0.01 (–0.13, 0.15) | 0.01 (–0.12, 0.14) |
|  | β_L2_ | – | 15.81 (–80.12, 111.70) | – | 15.79 (–80.12, 111.70) |
|  | β_H2_ | – | – | +0.00 (–0.04, 0.04) | +0.00 (–0.04, 0.04) |
|  | *p* value ^c^ | – | > 0.50 | > 0.50 | > 0.50 |
| 0.010 | β_L1_ | **–2.91 (–3.81, –1.88)** | –1.90 (–4.80, 0.99) | **–2.93 (–3.88, –1.97)** | –1.91 (–4.82, 1.00) |
|  | β_H1_ | 0.02 (–0.04, 0.09) | 0.02 (–0.04, 0.09) | 0.04 (–0.09, 0.17) | 0.04 (–0.09, 0.18) |
|  | β_L2_ | – | –8.24 (–30.20, 13.72) | – | –8.34 (–30.40, 13.73) |
|  | β_H2_ | – | – | –0.01 (–0.05, 0.03) | –0.01 (–0.05, 0.03) |
|  | *p* value ^c^ | – | 0.456 | > 0.50 | > 0.50 |
| 0.015 | β_L1_ | **–2.07 (–2.67, –1.39)** | **–2.20 (–4.14, –0.27)** | **–2.10 (–2.75, –1.45)** | **–2.23 (–4.18, –0.28)** |
|  | β_H1_ | 0.03 (–0.03, 0.10) | 0.03 (–0.04, 0.09) | 0.07 (–0.06, 0.19) | 0.06 (–0.06, 0.19) |
|  | β_L2_ | – | 0.77 (–9.65, 11.18) | – | 0.76 (–9.72, 11.25) |
|  | β_H2_ | – | – | –0.02 (–0.06, 0.03) | –0.01 (–0.06, 0.03) |
|  | *p* value ^c^ | – | > 0.50 | > 0.50 | > 0.50 |
| 0.020 | β_L1_ | **–1.38 (–1.85, –0.85)** | **–2.37 (–3.81, –0.93)** | **–1.42 (–1.94, –0.90)** | **–2.41 (–3.86, –0.96)** |
|  | β_H1_ | 0.04 (–0.03, 0.11) | 0.04 (–0.03, 0.10) | 0.09 (–0.04, 0.23) | 0.09 (–0.03, 0.22) |
|  | β_L2_ | – | 4.24 (–1.82, 10.29) | – | 4.26 (–1.84, 10.36) |
|  | β_H2_ | – | – | –0.02 (–0.06, 0.02) | –0.02 (–0.06, 0.02) |
|  | *p* value ^c^ | – | 0.163 | 0.353 | 0.247 |
| 0.025 | β_L1_ | **–0.96 (–1.35, –0.50)** | **–1.34 (–2.53, –0.15)** | **–1.00 (–1.44, –0.56)** | **–1.38 (–2.58, –0.18)** |
|  | β_H1_ | 0.04 (–0.02, 0.12) | 0.04 (–0.03, 0.11) | 0.11 (–0.02, 0.25) | 0.11 (–0.02, 0.24) |
|  | β_L2_ | – | 1.30 (–2.59, 5.19) | – | 1.31 (–2.61, 5.22) |
|  | β_H2_ | – | – | –0.03 (–0.07, 0.01) | –0.03 (–0.07, 0.01) |
|  | *p* value ^c^ | – | > 0.50 | 0.269 | 0.434 |
| 0.030 | β_L1_ | **–0.70 (–1.04, –0.30)** | **–1.20 (–2.20, –0.19)** | **–0.74 (–1.12, –0.36)** | **–1.25 (–2.26, –0.23)** |
|  | β_H1_ | 0.05 (–0.02, 0.13) | 0.05 (–0.03, 0.12) | 0.12 (–0.01, 0.26) | 0.12 (–0.01, 0.25) |
|  | β_L2_ | – | 1.44 (–1.32, 4.21) | – | 1.45 (–1.33, 4.23) |
|  | β_H2_ | – | – | –0.03 (–0.07, 0.01) | –0.03 (–0.07, 0.01) |
|  | *p* value ^c^ | – | 0.305 | 0.22 | 0.278 |
| 0.035 | β_L1_ | **–0.47 (–0.78, –0.11)** | **–0.97 (–1.83, –0.11)** | **–0.51 (–0.85, –0.17)** | **–1.00 (–1.87, –0.14)** |
|  | β_H1_ | 0.04 (–0.03, 0.13) | 0.04 (–0.03, 0.11) | 0.13 (–0.01, 0.26) | 0.12 (–0.01, 0.25) |
|  | β_L2_ | – | 1.24 (–0.76, 3.24) | – | 1.23 (–0.78, 3.24) |
|  | β_H2_ | – | – | –0.03 (–0.07, 0.01) | –0.03 (–0.07, 0.01) |
|  | *p* value ^c^ | – | 0.224 | 0.213 | 0.225 |
| 0.040 | β_L1_ | **–0.35 (–0.62, –0.03)** | **–0.94 (–1.67, –0.22)** | **–0.38 (–0.69, –0.07)** | **–0.98 (–1.71, –0.26)** |
|  | β_H1_ | 0.04 (–0.03, 0.12) | 0.04 (–0.03, 0.11) | 0.13 (–0.01, 0.26) | 0.12 (–0.01, 0.25) |
|  | β_L2_ | – | 1.27 (–0.19, 2.72) | – | 1.27 (–0.19, 2.74) |
|  | β_H2_ | – | – | –0.03 (–0.07, 0.01) | –0.03 (–0.07, 0.01) |
|  | *p* value ^c^ | – | 0.088 | 0.211 | 0.107 |
| 0.045 | β_L1_ | –0.24 (–0.49, 0.05) | **–0.77 (–1.41, –0.14)** | –0.27 (–0.55, 0.01) | **–0.81 (–1.45, –0.17)** |
|  | β_H1_ | 0.04 (–0.03, 0.12) | 0.04 (–0.03, 0.11) | 0.13 (–0.01, 0.26) | 0.12 (–0.01, 0.26) |
|  | β_L2_ | – | 0.98 (–0.13, 2.10) | – | 0.99 (–0.12, 2.11) |
|  | β_H2_ | – | – | –0.03 (–0.07, 0.01) | –0.03 (–0.07, +0.00) |
|  | *p* value ^c^ | – | 0.079 | 0.2 | 0.092 |
| 0.050 | β_L1_ | –0.17 (–0.41, 0.11) | **–0.68 (–1.27, –0.09)** | –0.20 (–0.46, 0.07) | **–0.72 (–1.31, –0.13)** |
|  | β_H1_ | 0.04 (–0.03, 0.12) | 0.03 (–0.04, 0.11) | 0.12 (–0.02, 0.26) | 0.12 (–0.01, 0.25) |
|  | β_L2_ | – | 0.87 (–0.08, 1.81) | – | 0.88 (–0.07, 1.83) |
|  | β_H2_ | – | – | –0.03 (–0.07, 0.01) | –0.03 (–0.07, +0.00) |
|  | *p* value ^c^ | – | 0.074 | 0.209 | 0.088 |
| *Notes:*  Numbers in bold indicate significant differences. The dataset for the analysis was stratified by sex, attained age, calendar period, smoking status, alcohol consumption, alpha dose.  CI, confidence interval. ERR/Gy, excess relative risk per unit gray of gamma-ray dose. IHD, ischemic heart disease (ICD-9 codes: 410–414). n/a denotes that it was not possible to estimate the lower or upper bound of the confidence interval.  ^a^ Equations of the models: (1) λ = λ_0_(1 + β_L1_D_L_ + β_H1_D_H_), (2) λ = λ_0_(1 + β_L1_d_L_+ β_L2_D_L_^2^ + β_Η1_D_H_), (3) λ = λ_0_(1 + β_L1_D_L_+ β_Η1_D_H_ + β_Η2_D_H_^2^),  (4) λ = λ_0_(1 + β_L1_D_L_ + β_L2_D_L_^2^ + β_Η1_D_H_ + β_Η2_D_H_^2^)  ^b^ Wald-type confidence interval.  ^c^ *p*-value denotes significant differences form a linear model. | | | | | |

Table S13 Excess relative risk per Gy of IHD mortality in relation to 10-year lagged cumulative liver absorbed gamma-ray doses from external exposure (non-linear analysis, females, entire cohort)

| Cutpoint,  Gy/year | Model  parameters | Models ^a^ | | | |
| --- | --- | --- | --- | --- | --- |
|  |  | (1) | (2) ^b^ | (3) ^b^ | (4) ^b^ |
| 0.005 | β_L1_ | –3.13 (–7.51, 2.70) | –2.62 (–15.16, 9.93) | –3.11 (–8.11, 1.90) | –2.49 (–14.82, 9.84) |
|  | β_H1_ | 0.14 (–0.03, 0.35) | 0.14 (–0.05, 0.33) | 0.03 (–0.36, 0.41) | 0.03 (–0.36, 0.41) |
|  | β_L2_ | – | –9.35 (–217.00, 198.30) | – | –11.54 (–215.60, 192.50) |
|  | β_H2_ | – | – | 0.06 (–0.12, 0.23) | 0.06 (–0.12, 0.23) |
|  | *p* value ^c^ | – | > 0.50 | > 0.50 | > 0.50 |
| 0.010 | β_L1_ | –2.20 (–4.00, 0.14) | 0.09 (–5.64, 5.81) | **–2.16 (–4.15, –0.16)** | 0.16 (–5.51, 5.82) |
|  | β_H1_ | 0.14 (–0.02, 0.35) | 0.14 (–0.05, 0.33) | 0.05 (–0.33, 0.43) | 0.05 (–0.34, 0.43) |
|  | β_L2_ | – | –20.28 (–63.82, 23.25) | – | –20.48 (–63.51, 22.56) |
|  | β_H2_ | – | – | 0.05 (–0.12, 0.22) | 0.05 (–0.12, 0.22) |
|  | *p* value ^c^ | – | 0.438 | > 0.50 | > 0.50 |
| 0.015 | β_L1_ | –1.58 (–2.85, 0.06) | –0.11 (–4.32, 4.09) | **–1.55 (–2.97, –0.13)** | –0.12 (–4.29, 4.05) |
|  | β_H1_ | 0.15 (–0.01, 0.37) | 0.16 (–0.04, 0.35) | 0.09 (–0.29, 0.47) | 0.09 (–0.30, 0.48) |
|  | β_L2_ | – | –9.19 (–32.99, 14.61) | – | –8.99 (–32.58, 14.60) |
|  | β_H2_ | – | – | 0.03 (–0.14, 0.20) | 0.03 (–0.14, 0.21) |
|  | *p* value ^c^ | – | 0.475 | > 0.50 | > 0.50 |
| 0.020 | β_L1_ | –1.26 (–2.27, 0.05) | –0.44 (–3.75, 2.87) | **–1.24 (–2.38, –0.11)** | –0.44 (–3.74, 2.85) |
|  | β_H1_ | 0.16 (–0.01, 0.38) | 0.16 (–0.03, 0.36) | 0.10 (–0.28, 0.48) | 0.11 (–0.28, 0.50) |
|  | β_L2_ | – | –4.01 (–18.98, 10.96) | – | –3.88 (–18.78, 11.02) |
|  | β_H2_ | – | – | 0.03 (–0.14, 0.20) | 0.03 (–0.15, 0.20) |
|  | *p* value ^c^ | – | > 0.50 | > 0.50 | > 0.50 |
| 0.025 | β_L1_ | –1.03 (–1.88, 0.10) | –0.30 (–2.98, 2.39) | **–1.01 (–1.98, –0.04)** | –0.30 (–2.97, 2.37) |
|  | β_H1_ | 0.16 (–0.01, 0.38) | 0.16 (–0.03, 0.36) | 0.10 (–0.28, 0.48) | 0.10 (–0.29, 0.49) |
|  | β_L2_ | – | –2.91 (–12.91, 7.09) | – | –2.84 (–12.80, 7.12) |
|  | β_H2_ | – | – | 0.03 (–0.14, 0.20) | 0.03 (–0.15, 0.20) |
|  | *p* value ^c^ | – | > 0.50 | > 0.50 | > 0.50 |
| 0.030 | β_L1_ | –0.59 (–1.42, 0.49) | –0.39 (–2.94, 2.16) | –0.58 (–1.52, 0.37) | –0.39 (–2.92, 2.14) |
|  | β_H1_ | 0.15 (–0.01, 0.37) | 0.15 (–0.04, 0.35) | 0.07 (–0.32, 0.46) | 0.07 (–0.32, 0.46) |
|  | β_L2_ | – | –0.75 (–9.65, 8.15) | – | –0.67 (–9.50, 8.15) |
|  | β_H2_ | – | – | 0.04 (–0.13, 0.22) | 0.04 (–0.14, 0.22) |
|  | *p* value ^c^ | – | > 0.50 | > 0.50 | > 0.50 |
| 0.035 | β_L1_ | –0.54 (–1.30, 0.45) | –0.21 (–2.54, 2.13) | –0.53 (–1.39, 0.33) | –0.20 (–2.52, 2.11) |
|  | β_H1_ | 0.16 (–0.01, 0.38) | 0.16 (–0.04, 0.36) | 0.08 (–0.30, 0.47) | 0.08 (–0.31, 0.48) |
|  | β_L2_ | – | –1.12 (–8.45, 6.22) | – | –1.08 (–8.35, 6.20) |
|  | β_H2_ | – | – | 0.04 (–0.14, 0.21) | 0.04 (–0.14, 0.22) |
|  | *p* value ^c^ | – | > 0.50 | > 0.50 | > 0.50 |
| 0.040 | β_L1_ | –0.39 (–1.12, 0.55) | –0.38 (–2.54, 1.79) | –0.38 (–1.21, 0.45) | –0.37 (–2.51, 1.77) |
|  | β_H1_ | 0.16 (–0.01, 0.38) | 0.16 (–0.04, 0.36) | 0.08 (–0.31, 0.48) | 0.09 (–0.31, 0.48) |
|  | β_L2_ | – | –0.06 (–6.43, 6.31) | – | –0.04 (–6.35, 6.27) |
|  | β_H2_ | – | – | 0.04 (–0.14, 0.22) | 0.04 (–0.14, 0.22) |
|  | *p* value ^c^ | – | > 0.50 | > 0.50 | > 0.50 |
| 0.045 | β_L1_ | –0.24 (–0.95, 0.69) | –0.47 (–2.55, 1.61) | –0.22 (–1.04, 0.60) | –0.46 (–2.52, 1.61) |
|  | β_H1_ | 0.16 (–0.01, 0.38) | 0.16 (–0.04, 0.35) | 0.07 (–0.32, 0.47) | 0.07 (–0.32, 0.46) |
|  | β_L2_ | – | 0.72 (–5.17, 6.61) | – | 0.72 (–5.11, 6.55) |
|  | β_H2_ | – | – | 0.04 (–0.14, 0.23) | 0.04 (–0.14, 0.22) |
|  | *p* value ^c^ | – | > 0.50 | > 0.50 | > 0.50 |
| 0.050 | β_L1_ | –0.19 (–0.88, 0.70) | –0.43 (–2.38, 1.53) | –0.18 (–0.97, 0.61) | –0.41 (–2.35, 1.53) |
|  | β_H1_ | 0.15 (–0.02, 0.38) | 0.15 (–0.04, 0.35) | 0.07 (–0.33, 0.46) | 0.07 (–0.33, 0.46) |
|  | β_L2_ | – | 0.68 (–4.53, 5.88) | – | 0.68 (–4.48, 5.84) |
|  | β_H2_ | – | – | 0.04 (–0.14, 0.23) | 0.04 (–0.14, 0.23) |
|  | *p* value ^c^ | – | > 0.50 | > 0.50 | > 0.50 |
| *Notes:*  Numbers in bold indicate significant differences. The dataset for the analysis was stratified by sex, attained age, calendar period, smoking status, alcohol consumption, alpha dose.  CI, confidence interval. ERR/Gy, excess relative risk per unit gray of gamma-ray dose. IHD, ischemic heart disease (ICD-9 codes: 410–414). n/a denotes that it was not possible to estimate the lower or upper bound of the confidence interval.  ^a^ Equations of the models: (1) λ = λ_0_(1 + β_L1_D_L_ + β_H1_D_H_), (2) λ = λ_0_(1 + β_L1_D_L_+ β_L2_D_L_^2^ + β_Η1_D_H_), (3) λ = λ_0_(1 + β_L1_D_L_+ β_Η1_D_H_ + β_Η2_D_H_^2^),  (4) λ = λ_0_(1 + β_L1_D_L_ + β_L2_D_L_^2^ + β_Η1_D_H_ + β_Η2_D_H_^2^)  ^b^ Wald-type confidence interval.  ^c^ *p*-value denotes significant differences from a linear model. | | | | | |

Table S14 Variables used in the model for analyses

| Type | Variable | Description | |
| --- | --- | --- | --- |
| Fixed | Sex | 1: M | 2: F |
| Fixed | Period of hire | 1: 1948–1958  2: 1958–1972 | 3: 1973–1982 |
| Fixed | Age at hire | 1: <20  2: 20–25 | 3: 25–30  4: ≥30 |
| Fixed | Smoking status | 1: never smoker  2: ex-smoker | 3: smoker  4: unknown |
| Fixed | Alcohol drinking habit status | 1: never-drinker  2: seldom-drinker  3: moderate-drinker | 4: heavy-drinker  5: unknown |
| Fixed | Duration of employment (year) | 1: <1  2: ≥1 |  |
| Time-dependent | Attained age | 1: <20  2: 20–25  3: 25–30  4: 30–35  5: 35–40  6: 40–45  7: 45–50  8: 50–55 | 9: 55–60  10: 60–65  11: 65–70  12: 70–75  13: 75–80  14: 80–85  15: ≥85 |
| Time-dependent | Calendar period | 1: 1946–1950  2: 1951–1955  3: 1956–1960  4: 1961–1965  5: 1966–1970  6: 1971–1975  7: 1976–1980 | 8: 1981–1985  9: 1986–1990  10: 1991–1995  11: 1996–2000  12: 2001–2005  13: 2006–2010  14: 2011–2015  15: 2016–2019 |
| Time-dependent | Liver absorbed gamma-ray dose from external exposure (Gy), lagged for 0, 5, 10, 20, 30 years | 1: 0–0.1  2: 0.1–0.2  3: 0.2–0.5  4: 0.5–0.75  5: 0.75–1.00 | 6: 1.00–1.50  7: 1.50–2.00  8: 2.00–3.00  6: ≥3.00 |
| Time-dependent | Cumulative liver absorbed gamma-ray dose from external exposure of the dose rate that is lower than the specified dose rate cutpoint (Gy), lagged for 0, 5, 10, 20, 30 years | 1: 0–0.1  2: 0.1–0.2  3: 0.2–0.5  4: 0.5–0.75  5: 0.75–1.00 | 6: 1.00–1.50  7: 1.50–2.00  8: 2.00–3.00  6: ≥3.00 |
| Time-dependent | Cumulative liver absorbed gamma-ray dose from external exposure of the dose rate that is higher than the specified dose rate cutpoint (Gy), lagged for 0, 5, 10, 20, 30 years | 1: 0–0.1  2: 0.1–0.2  3: 0.2–0.5  4: 0.5–0.75  5: 0.75–1.00 | 6: 1.00–1.50  7: 1.50–2.00  8: 2.00–3.00  6: ≥3.00 |
| Time-dependent | Liver absorbed alpha dose from internal exposure (Gy), lagged for 0, 5, 10, 20, 30 years | 1: unmeasured  2: 0.00–0.025  3: 0.025–0.05  4: 0.05–0.1  5: 0.1–0.15 | 6 0.15–0.25  7: 0.25–0.5  8: 0.5 –1.00  9: ≥1.00 |
| Time-dependent | Weighted sum of liver absorbed gamma-ray + neutron dose (Gy), lagged for 10 years | 1: 0–0.1  2: 0.1–0.2  3: 0.2–0.5  4: 0.5–0.75  5: 0.75–1.00 | 6: 1.00–1.50  7: 1.50–2.00  8: 2.00–3.00  6: ≥3.00 |
| Time-dependent | Cumulative weighted sum of liver absorbed gamma-ray + neutron dose (Gy) of the dose rate that is lower than the specified gamma-ray dose rate cutpoint, lagged for 0, 5, 10, 20, 30 years | 1: 0–0.1  2: 0.1–0.2  3: 0.2–0.5  4: 0.5–0.75  5: 0.75–1.00 | 6: 1.00–1.50  7: 1.50–2.00  8: 2.00–3.00  6: ≥3.00 |
| Time-dependent | Cumulative weighted sum of liver absorbed gamma-ray + neutron dose (Gy) of the dose rate that is higher than the specified gamma-ray dose rate cutpoint, lagged for 0, 5, 10, 20, 30 years | 1: 0–0.1  2: 0.1–0.2  3: 0.2–0.5  4: 0.5–0.75  5: 0.75–1.00 | 6: 1.00–1.50  7: 1.50–2.00  8: 2.00–3.00  6: ≥3.00 |
| Estimated | Attained age | Person-years weighted by mean age (in years) | |
|  | Cumulative liver absorbed gamma-ray dose from external exposure (Gy),  lagged for 0, 5, 10, 20, 30 years | Person-years weighted by cumulative liver absorbed gamma-ray dose lagged for 0, 5, 10, 20, 30 years | |
|  | Cumulative weighted sum of liver absorbed gamma-ray + neutron dose (Gy), lagged for 10 years | Person-years weighted by cumulative weighted sum of liver absorbed gamma-ray + neutron dose lagged for 10 years | |
|  | Cumulative liver absorbed alpha dose from internal exposure (Gy),  lagged for 0, 5, 10, 20, 30 years | Person-years weighted by cumulative liver absorbed alpha dose lagged for 0, 5, 10, 20, 30 years | |
|  | Person-years | at risk | |
|  | Deaths | 1: IHD: ICD-9 codes 410–414 | |

Data on smoking habits were taken into account over the entire follow-up period and estimated with qualitative and quantitative indices. The qualitative index included values ‘unknown’, ‘never smoker’, ‘ex-smoker’ and ‘smoker’. ‘Never smoker’ was assumed to be a worker who reported during a series of annual mandatory medical examinations that he/she had never smoked. For stratification purposes, categories ‘ex-smoker’ and ‘smoker’ were combined to build one category ‘ever smoker’. The quantitative measure was referred to as the smoking index measured in pack*years (calculated as a mean number of cigarette packs (20 cigarettes/pack) smoked in a day times years of smoking) and for ‘never-smokers’ was equated with zero.

Data on the alcohol drinking habit were also taken into account over the entire follow-up period and estimated only with a qualitative parameter with values ‘unknown’, ‘seldom-drinker’, ‘moderate-drinker’ and ‘heavy-drinker’. A ‘seldom-drinker’ was assumed to be a worker who reported during a series of annual mandatory medical examinations that he/she had never drunk alcohol or drank seldomly just small amounts. A ‘moderate drinker’ was assumed to be a worker who stated during mandatory health check-ups that he/she drank moderately. A ‘heavy drinker’ was assumed to be a worker whose medical files contained information on incidents of abusive drinking behavior, inebriety or chronic alcoholism.

Table S15 Distribution of person years and deaths from IHD in the study cohort (entire cohort) by cumulative liver absorbed gamma-ray dose from external exposure (gamma dose lagged for 10 years)

| Cumulative dose accumulated at a dose rate lower than a dose cutpoint (D_γL_), Gy | Cumulative dose accumulated at a dose rate  higher than a cutpoint (D_γH_), Gy | | | |
| --- | --- | --- | --- | --- |
|  | 0.0–0.1 | 0.1–0.2 | 0.2–0.5 | ≥0.5 |
| *Cutpoint 0.005 Gy/year* | | | | |
| 0.0–0.1 | 489742 / 1212 | 85054 / 416 | 124100 / 672 | 172826 / 1181 |
| 0.1–0.2 | 19 / 0 | 4 / 0 | 0 / 0 | 0 / 0 |
| 0.2–0.5 | 0 / 0 | 0 / 0 | 0 / 0 | 0 / 0 |
| ≥0.5 | 0 / 0 | 0 / 0 | 0 / 0 | 0 / 0 |
| *Cutpoint 0.010 Gy/year* | | | | |
| 0.0–0.1 | 501505 / 1274 | 73371 / 339 | 109330 / 558 | 165282 / 1111 |
| 0.1–0.2 | 8934 / 65 | 4060 / 34 | 5255 / 57 | 4000 / 42 |
| 0.2–0.5 | 4 / 0 | 0 / 0 | 0 / 0 | 4 / 1 |
| ≥0.5 | 0 / 0 | 0 / 0 | 0 / 0 | 0 / 0 |
| *Cutpoint 0.015 Gy/year* | | | | |
| 0.0–0.1 | 501991 / 1258 | 61803 / 264 | 92889 / 436 | 151308 / 958 |
| 0.1–0.2 | 23947 / 164 | 10833 / 92 | 13025 / 124 | 13513 / 161 |
| 0.2–0.5 | 604 / 4 | 699 / 8 | 688 / 8 | 445 / 4 |
| ≥0.5 | 0 / 0 | 0 / 0 | 0 / 0 | 0 / 0 |
| *Cutpoint 0.020 Gy/year* | | | | |
| 0.0–0.1 | 499918 / 1249 | 51467 / 210 | 82242 / 378 | 138479 / 812 |
| 0.1–0.2 | 37359 / 228 | 12698 / 83 | 14205 / 113 | 20307 / 236 |
| 0.2–0.5 | 4632 / 40 | 3346 / 38 | 4071 / 51 | 3021 / 43 |
| ≥0.5 | 0 / 0 | 0 / 0 | 0 / 0 | 0 / 0 |
| *Cutpoint 0.025 Gy/year* | | | | |
| 0.0–0.1 | 498436 / 1230 | 41732 / 166 | 72251 / 321 | 127590 / 724 |
| 0.1–0.2 | 48820 / 287 | 12223 / 72 | 14669 / 101 | 22637 / 236 |
| 0.2–0.5 | 12173 / 97 | 5399 / 54 | 7706 / 83 | 8109 / 110 |
| ≥0.5 | 0 / 0 | 0 / 0 | 0 / 0 | 0 / 0 |
| *Cutpoint 0.030 Gy/year* | | | | |
| 0.0–0.1 | 496037 / 1215 | 34857 / 143 | 64326 / 292 | 118182 / 637 |
| 0.1–0.2 | 56876 / 318 | 11432 / 53 | 14490 / 88 | 23694 / 232 |
| 0.2–0.5 | 20791 / 164 | 7611 / 65 | 10750 / 101 | 12539 / 170 |
| ≥0.5 | 5 / 0 | 58 / 2 | 64 / 1 | 33 / 0 |
| *Cutpoint 0.035 Gy/year* | | | | |
| 0.0–0.1 | 494688 / 1204 | 29759 / 123 | 58726 / 265 | 110453 / 577 |
| 0.1–0.2 | 61850 / 330 | 9534 / 47 | 14961 / 83 | 23456 / 212 |
| 0.2–0.5 | 30420 / 228 | 7961 / 61 | 12403 / 119 | 16313 / 214 |
| ≥0.5 | 165 / 1 | 268 / 3 | 526 / 8 | 262 / 6 |
| *Cutpoint 0.040 Gy/year* | | | | |
| 0.0–0.1 | 490772 / 1177 | 27427 / 116 | 53615 / 237 | 103367 / 534 |
| 0.1–0.2 | 65750 / 354 | 9421 / 42 | 15300 / 90 | 23919 / 197 |
| 0.2–0.5 | 37508 / 260 | 8525 / 77 | 13964 / 114 | 18626 / 230 |
| ≥0.5 | 684 / 8 | 698 / 9 | 1231 / 19 | 938 / 17 |
| *Cutpoint 0.045 Gy/year* | | | | |
| 0.0–0.1 | 488218 / 1166 | 24201 / 110 | 49885 / 221 | 96948 / 480 |
| 0.1–0.2 | 69231 / 360 | 9579 / 40 | 15147 / 87 | 24498 / 202 |
| 0.2–0.5 | 43150 / 297 | 8915 / 69 | 15065 / 115 | 20458 / 234 |
| ≥0.5 | 1593 / 16 | 1141 / 15 | 2204 / 41 | 1512 / 28 |
| *Cutpoint 0.050 Gy/year* | | | | |
| 0.0–0.1 | 487260 / 1164 | 21593 / 91 | 46285 / 202 | 92058 / 456 |
| 0.1–0.2 | 72711 / 377 | 9050 / 38 | 14761 / 85 | 24852 / 191 |
| 0.2–0.5 | 48147 / 328 | 8833 / 69 | 15103 / 109 | 21920 / 239 |
| ≥0.5 | 2574 / 24 | 1619 / 19 | 2963 / 49 | 2016 / 40 |
| *Notes:*  The numbers presented are person years / deaths from IHD.  IHD, ischemic heart disease (ICD-9 codes: 410–414). | | | | |

Table S16 Distribution of person years and deaths from IHD in the resident subcohort by cumulative liver absorbed gamma-ray dose from external exposure (gamma dose lagged for 10 years)

| Cumulative dose accumulated at a dose rate lower than a dose cutpoint (D_γL_), Gy | Cumulative dose accumulated at a dose rate  higher than a cutpoint (D_γH_), Gy | | | |
| --- | --- | --- | --- | --- |
|  | 0.0–0.1 | 0.1–0.2 | 0.2–0.5 | ≥0.5 |
| *Cutpoint 0.005 Gy/year* | | | | |
| 0.0–0.1 | 387676 / 774 | 52755 / 267 | 75710 / 437 | 106035 / 789 |
| 0.1–0.2 | 19 / 0 | 4 / 0 | 0 / 0 | 0 / 0 |
| 0.2–0.5 | 0 / 0 | 0 / 0 | 0 / 0 | 0 / 0 |
| ≥0.5 | 0 / 0 | 0 / 0 | 0 / 0 | 0 / 0 |
| *Cutpoint 0.010 Gy/year* | | | | |
| 0.0–0.1 | 396442 / 813 | 42448 / 204 | 62364 / 330 | 98929 / 723 |
| 0.1–0.2 | 8928 / 65 | 3998 / 34 | 5190 / 57 | 3892 / 41 |
| 0.2–0.5 | 4 / 0 | 0 / 0 | 0 / 0 | 4 / 0 |
| ≥0.5 | 0 / 0 | 0 / 0 | 0 / 0 | 0 / 0 |
| *Cutpoint 0.015 Gy/year* | | | | |
| 0.0–0.1 | 393920 / 784 | 32948 / 141 | 47659 / 219 | 85873 / 573 |
| 0.1–0.2 | 23484 / 161 | 10374 / 86 | 12531 / 123 | 12981 / 157 |
| 0.2–0.5 | 604 / 4 | 692 / 7 | 688 / 8 | 445 / 4 |
| ≥0.5 | 0 / 0 | 0 / 0 | 0 / 0 | 0 / 0 |
| *Cutpoint 0.020 Gy/year* | | | | |
| 0.0–0.1 | 389913 / 769 | 25104 / 98 | 38866 / 168 | 74521 / 444 |
| 0.1–0.2 | 35387 / 216 | 11745 / 75 | 12852 / 105 | 18983 / 221 |
| 0.2–0.5 | 4527 / 39 | 3302 / 38 | 4029 / 51 | 2970 / 43 |
| ≥0.5 | 0 / 0 | 0 / 0 | 0 / 0 | 0 / 0 |
| *Cutpoint 0.025 Gy/year* | | | | |
| 0.0–0.1 | 387330 / 749 | 18121 / 65 | 31923 / 123 | 65485 / 373 |
| 0.1–0.2 | 43961 / 261 | 10355 / 60 | 12214 / 91 | 20265 / 211 |
| 0.2–0.5 | 11898 / 92 | 5233 / 53 | 7503 / 81 | 7911 / 108 |
| ≥0.5 | 0 / 0 | 0 / 0 | 0 / 0 | 0 / 0 |
| *Cutpoint 0.030 Gy/year* | | | | |
| 0.0–0.1 | 384638 / 732 | 14406 / 53 | 26696 / 106 | 58357 / 309 |
| 0.1–0.2 | 48949 / 279 | 8734 / 38 | 10922 / 76 | 19913 / 187 |
| 0.2–0.5 | 19847 / 156 | 7350 / 63 | 10202 / 99 | 12024 / 166 |
| ≥0.5 | 5 / 0 | 59 / 2 | 64 / 1 | 33 / 0 |
| *Cutpoint 0.035 Gy/year* | | | | |
| 0.0–0.1 | 383229 / 723 | 11709 / 39 | 23193 / 89 | 52797 / 265 |
| 0.1–0.2 | 51116 / 286 | 6825 / 26 | 10641 / 67 | 18933 / 164 |
| 0.2–0.5 | 28375 / 216 | 7395 / 58 | 11595 / 114 | 15186 / 202 |
| ≥0.5 | 165 / 1 | 268 / 3 | 510 / 8 | 262 / 6 |
| *Cutpoint 0.040 Gy/year* | | | | |
| 0.0–0.1 | 380984 / 710 | 10397 / 34 | 20470 / 76 | 48212 / 234 |
| 0.1–0.2 | 52367 / 292 | 6269 / 25 | 10286 / 66 | 18088 / 150 |
| 0.2–0.5 | 34398 / 240 | 7495 / 72 | 12756 / 106 | 16981 / 209 |
| ≥0.5 | 658 / 8 | 698 / 9 | 1212 / 19 | 928 / 17 |
| *Cutpoint 0.045 Gy/year* | | | | |
| 0.0–0.1 | 379359 / 700 | 9114 / 31 | 18340 / 68 | 44080 / 196 |
| 0.1–0.2 | 53488 / 290 | 6264 / 27 | 9566 / 59 | 17899 / 153 |
| 0.2–0.5 | 38671 / 272 | 7547 / 64 | 13448 / 99 | 18011 / 208 |
| ≥0.5 | 1548 / 16 | 1230 / 15 | 2156 / 41 | 1478 / 28 |
| *Cutpoint 0.050 Gy/year* | | | | |
| 0.0–0.1 | 378396 / 696 | 7803 / 22 | 16356 / 61 | 41259 / 181 |
| 0.1–0.2 | 55408 / 302 | 5501 / 19 | 8843 / 58 | 17213 / 140 |
| 0.2–0.5 | 42476 / 294 | 7576 / 63 | 13186 / 91 | 19242 / 208 |
| ≥0.5 | 2500 / 24 | 1611 / 19 | 2880 / 49 | 1949 / 40 |
| *Notes:*  The numbers presented are person years / deaths from IHD.  IHD, ischemic heart disease (ICD-9 codes: 410–414). | | | | |

| Table S17 Distributions of Mayak workers by cumulative neutron and alpha doses from external and internal exposures | | |
| --- | --- | --- |
| Dose, Gy ^a^ | Entire cohort | Resident subcohort |
| *Cumulative liver absorbed neutron dose from external exposure (p<0.001 ^b^)* | | |
| Non-measured | 18,294 (13,213/5081) | 10,573 (7302/3271) |
| 0.00–0.0001 | 998 (809/189) | 509 (407/102) |
| 0.0001–0.001 | 2025 (1715/310) | 1261 (1047/214) |
| 0.001–0.0025 | 759 (696/63) | 602 (545/57) |
| 0.0025–0.005 | 174 (160/14) | 125 (116/9) |
| 0.005–0.01 | 74 (59/15) | 50 (43/7) |
| ≥0.01 | 53 (36/17) | 36 (24/12) |
| *Cumulative liver absorbed alpha dose from internal exposure (p=0.67 ^c^)* | | |
| Non-measured | 14,371 (11,114/3257) | 5544 (4196/1348) |
| 0–0.025 | 3645 (2567/1078) | 3563 (2497/1066) |
| 0.025–0.05 | 1131 (801/330) | 1077 (762/315) |
| 0.05–0.10 | 987 (712/275) | 930 (672/258) |
| 0.10–0.15 | 483 (346/137) | 454 (325/129) |
| 0.15–0.25 | 547 (361/186) | 504 (329/175) |
| 0.25–0.5 | 571 (373/198) | 528 (342/186) |
| 0.5–1.0 | 300 (205/95) | 270 (185/85) |
| ≥1.0 | 342 (209/133) | 286 (176/110) |
| *Notes:*  ^a^The numbers presented are for both sexes, with those for males/females indicated in parenthesis.  ^b^ Difference of distributions of workers by the cumulative liver absorbed neutron dose from external exposure between the entire cohort and the resident subcohort (exclude non-measured).  ^c^ Difference of distributions of workers by the cumulative liver alpha dose from internal exposure between the entire cohort and the resident subcohort (exclude non-measured). | | |

Table S18 Excess relative risk per Gy of IHD mortality in relation to cumulative liver absorbed gamma-ray doses from external radiation exposure, adjusted for various non-radiation factors and alpha absorbed dose to the liver (sensitivity analyses – various lag periods, males, residents)

| Cutpoint,  Gy/year | Model  parameters | Lag periods | | | |
| --- | --- | --- | --- | --- | --- |
|  |  | 0 years | 5 years | 20 years | 30 years |
| 0 (without cutpoint [16]) | ERR/Gy | 0.08 (–0.02, 0.19) | 0.06 (–0.03, 0.18) | 0.05 (–0.05, 0.17) | 0.04 (–0.06, 0.17) |
| 0.005 | ERR_L_/Gy | **–6.15 (–7.53, –4.43)** | **–5.61 (–7.32, –3.57)** | **–5.13 (–8.12, –1.48)** | –5.81 (–11.18, 0.89) |
|  | ERR_H_/Gy | 0.05 (–0.03, 0.15) | 0.05 (–0.04, 0.15) | 0.03 (–0.06, 0.15) | 0.03 (–0.07, 0.16) |
|  | *p* value ^a^ | **< 0.001** | 0.29 | **0.008** | 0.082 |
| 0.010 | ERR_L_/Gy | **–3.41 (–4.14, –2.55)** | **–3.19 (–3.99, –2.24)** | **–3.19 (–4.39, –1.75)** | **–3.85 (–5.70, –1.55)** |
|  | ERR_H_/Gy | 0.08 (–0.00, 0.19) | 0.07 (–0.01, 0.17) | 0.05 (–0.04, 0.15) | 0.03 (–0.06, 0.15) |
|  | *p* value ^a^ | **< 0.001** | 0.105 | **< 0.001** | **0.002** |
| 0.015 | ERR_L_/Gy | **–2.33 (–2.85, –1.72)** | **–2.21 (–2.77, –1.55)** | **–2.10 (–2.90, –1.15)** | **–2.80 (na, –1.45)** |
|  | ERR_H_/Gy | **0.10 (0.01, 0.21)** | 0.08 (–0.00, 0.19) | 0.05 (–0.04, 0.16) | 0.04 (–0.06, 0.16) |
|  | *p* value ^a^ | **< 0.001** | 0.059 | **< 0.001** | **< 0.001** |
| 0.020 | ERR_L_/Gy | **–1.57 (–2.00, –1.07)** | **–1.48 (–1.93, –0.94)** | **–1.37 (–1.97, –0.64)** | **–1.71 (–2.53, –0.72)** |
|  | ERR_H_/Gy | **0.12 (0.03, 0.24)** | **0.11 (0.01, 0.22)** | 0.07 (–0.03, 0.19) | 0.05 (–0.05, 0.17) |
|  | *p* value ^a^ | **< 0.001** | **0.023** | **< 0.001** | **0.001** |
| 0.025 | ERR_L_/Gy | **–1.13 (–1.50, –0.70)** | **–1.06 (–1.45, –0.60)** | **–0.88 (–1.40, –0.26)** | **–1.22 (–1.87, –0.42)** |
|  | ERR_H_/Gy | **0.14 (0.04, 0.26)** | **0.12 (0.02, 0.24)** | 0.08 (–0.02, 0.20) | 0.07 (–0.04, 0.20) |
|  | *p* value ^a^ | **< 0.001** | **0.014** | **0.005** | **0.003** |
| 0.030 | ERR_L_/Gy | **–0.82 (–1.16, –0.43)** | **–0.76 (–1.11, –0.34)** | **–0.60 (–1.05, –0.06)** | **–0.85 (–1.41, –0.16)** |
|  | ERR_H_/Gy | **0.15 (0.05, 0.28)** | **0.13 (0.02, 0.25)** | 0.08 (–0.02, 0.21) | 0.07 (–0.04, 0.20) |
|  | *p* value ^a^ | **< 0.001** | **0.013** | **0.018** | **0.013** |
| 0.035 | ERR_L_/Gy | **–0.55 (–0.86, –0.19)** | **–0.49 (–0.81, –0.11)** | –0.30 (–0.72, 0.20) | –0.50 (–1.02, 0.13) |
|  | ERR_H_/Gy | **0.15 (0.04, 0.28)** | **0.12 (0.02, 0.25)** | 0.07 (–0.03, 0.20) | 0.07 (–0.04, 0.20) |
|  | *p* value ^a^ | **< 0.001** | **0.022** | 0.15 | 0.081 |
| 0.040 | ERR_L_/Gy | **–0.40 (–0.68, –0.07)** | **–0.34 (–0.64, –0.00)** | –0.19 (–0.56, 0.26) | –0.35 (–0.82, 0.21) |
|  | ERR_H_/Gy | **0.14 (0.03, 0.28)** | **0.12 (0.01, 0.25)** | 0.07 (–0.04, 0.20) | 0.06 (–0.05, 0.20) |
|  | *p* value ^a^ | **0.004** | **0.03** | 0.261 | 0.144 |
| 0.045 | ERR_L_/Gy | –0.27 (–0.53, 0.03) | –0.23 (–0.50, 0.09) | –0.08 (–0.42, 0.32) | –0.19 (–0.61, 0.32) |
|  | ERR_H_/Gy | **0.14 (0.03, 0.28)** | **0.11 (+0.00, 0.25)** | 0.06 (–0.04, 0.20) | 0.06 (–0.05, 0.20) |
|  | *p* value ^a^ | **0.018** | **0.042** | 0.475 | 0.333 |
| 0.050 | ERR_L_/Gy | –0.18 (–0.43, 0.11) | –0.15 (–0.41, 0.15) | +0.00 (–0.32, 0.38) | –0.09 (–0.49, 0.40) |
|  | ERR_H_/Gy | **0.13 (0.02, 0.27)** | 0.10 (–0.00, 0.24) | 0.05 (–0.05, 0.19) | 0.05 (–0.06, 0.20) |
|  | *p* value ^a^ | 0.058 | 0.13 | > 0.50 | > 0.50 |
| *Notes:*  Numbers in bold indicate significant differences. The dataset for the analysis was stratified by sex, attained age, calendar period, smoking status, alcohol consumption, alpha dose.  CI, confidence interval. ERR/Gy, excess relative risk per unit gray of gamma-ray dose. IHD, ischemic heart disease (ICD-9 codes: 410–414). n/a denotes that it was not possible to estimate the lower or upper bound of the confidence interval.  ^a^ Likelihood ratio test comparing the models with and without cutpoint. | | | | | |

Table 19 Excess relative risk per Gy of IHD mortality in relation to cumulative liver absorbed gamma-ray doses from external radiation exposure, adjusted for various non-radiation factors and alpha absorbed dose to the liver (sensitivity analyses – various lag periods, females, residents)

| Cutpoint,  Gy/year | Model  parameters | Lag periods | | | |
| --- | --- | --- | --- | --- | --- |
|  |  | 0 years | 5 years | 20 years | 30 years |
| 0 (without cutpoint [16]) | ERR/Gy | 0.13 (–0.08, 0.43) | 0.14 (–0.07, 0.44) | 0.13 (–0.08, 0.43) | 0.13 (–0.08, 0.45) |
| 0.005 | ERR_L_/Gy | –2.83 (–7.07, 2.96) | –2.50 (–6.93, 3.51) | –1.96 (–7.51, 5.66) | –4.20 (–11.01, 5.78) |
|  | ERR_H_/Gy | 0.12 (–0.08, 0.41) | 0.13 (–0.07, 0.42) | 0.12 (–0.09, 0.41) | 0.12 (–0.09, 0.42) |
|  | *p* value ^a^ | 0.281 | 0.248 | > 0.50 | 0.351 |
| 0.010 | ERR_L_/Gy | –2.01 (–3.82, 0.38) | –1.93 (–3.79, 0.52) | –1.73 (–3.88, 1.16) | –1.44 (–4.14, 2.27) |
|  | ERR_H_/Gy | 0.13 (–0.07, 0.41) | 0.13 (–0.07, 0.42) | 0.12 (–0.08, 0.40) | 0.12 (–0.09, 0.42) |
|  | *p* value ^a^ | 0.074 | 0.213 | 0.184 | 0.364 |
| 0.015 | ERR_L_/Gy | –1.39 (–2.70, 0.34) | –1.32 (–2.67, 0.46) | –1.02 (–2.57, 1.06) | –0.82 (–2.65, 1.69) |
|  | ERR_H_/Gy | 0.15 (–0.05, 0.44) | 0.15 (–0.05, 0.44) | 0.13 (–0.07, 0.43) | 0.14 (–0.08, 0.45) |
|  | *p* value ^a^ | 0.077 | 0.168 | 0.247 | 0.41 |
| 0.020 | ERR_L_/Gy | –1.21 (–2.23, 0.14) | –1.12 (–2.17, 0.28) | –0.93 (–2.12, 0.66) | –0.80 (–2.19, 1.10) |
|  | ERR_H_/Gy | 0.16 (–0.05, 0.45) | 0.16 (–0.05, 0.45) | 0.14 (–0.07, 0.44) | 0.15 (–0.07, 0.46) |
|  | *p* value ^a^ | **0.048** | 0.157 | 0.168 | 0.293 |
| 0.025 | ERR_L_/Gy | –1.10 (–1.94, 0.03) | –1.00 (–1.87, 0.18) | –0.86 (–1.84, 0.47) | –0.90 (–2.00, 0.62) |
|  | ERR_H_/Gy | 0.16 (–0.05, 0.45) | 0.16 (–0.05, 0.46) | 0.14 (–0.06, 0.44) | 0.15 (–0.07, 0.46) |
|  | *p* value ^a^ | **0.031** | 0.151 | 0.125 | 0.157 |
| 0.030 | ERR_L_/Gy | –0.68 (–1.49, 0.41) | –0.59 (–1.43, 0.54) | –0.43 (–1.36, 0.84) | –0.45 (–1.49, 0.99) |
|  | ERR_H_/Gy | 0.15 (–0.06, 0.45) | 0.15 (–0.06, 0.45) | 0.14 (–0.07, 0.44) | 0.14 (–0.07, 0.46) |
|  | *p* value ^a^ | 0.12 | 0.172 | 0.343 | 0.377 |
| 0.035 | ERR_L_/Gy | –0.66 (–1.39, 0.33) | –0.59 (–1.34, 0.43) | –0.43 (–1.27, 0.71) | –0.44 (–1.38, 0.86) |
|  | ERR_H_/Gy | 0.16 (–0.05, 0.46) | 0.16 (–0.05, 0.46) | 0.14 (–0.07, 0.44) | 0.15 (–0.07, 0.46) |
|  | *p* value ^a^ | 0.098 | 0.161 | 0.289 | 0.334 |
| 0.040 | ERR_L_/Gy | –0.52 (–1.23, 0.43) | –0.45 (–1.18, 0.53) | –0.31 (–1.11, 0.79) | –0.34 (–1.24, 0.89) |
|  | ERR_H_/Gy | 0.16 (–0.05, 0.47) | 0.16 (–0.05, 0.46) | 0.14 (–0.07, 0.45) | 0.15 (–0.07, 0.47) |
|  | *p* value ^a^ | 0.147 | 0.158 | 0.384 | 0.395 |
| 0.045 | ERR_L_/Gy | –0.29 (–1.01, 0.67) | –0.25 (–0.98, 0.73) | –0.09 (–0.90, 1.00) | –0.12 (–1.02, 1.11) |
|  | ERR_H_/Gy | 0.16 (–0.06, 0.46) | 0.15 (–0.06, 0.46) | 0.14 (–0.08, 0.44) | 0.14 (–0.08, 0.46) |
|  | *p* value ^a^ | 0.33 | 0.179 | > 0.50 | > 0.50 |
| 0.050 | ERR_L_/Gy | –0.30 (–0.98, 0.61) | –0.25 (–0.94, 0.68) | –0.13 (–0.88, 0.89) | –0.18 (–1.00, 0.95) |
|  | ERR_H_/Gy | 0.16 (–0.06, 0.46) | 0.15 (–0.06, 0.46) | 0.14 (–0.08, 0.44) | 0.15 (–0.08, 0.47) |
|  | *p* value ^a^ | 0.299 | 0.363 | > 0.50 | > 0.50 |
| *Notes:*  Numbers in bold indicate significant differences. The dataset for the analysis was stratified by sex, attained age, calendar period, smoking status, alcohol consumption, alpha dose.  CI, confidence interval. ERR/Gy, excess relative risk per unit gray of gamma-ray dose. IHD, ischemic heart disease (ICD-9 codes: 410–414). n/a denotes that it was not possible to estimate the lower or upper bound of the confidence interval.  ^a^ Likelihood ratio test comparing the models with and without cutpoint. | | | | | |

Table S20 Excess relative risk per Gy of IHD mortality in relation to 10-year lagged cumulative liver absorbed gamma-ray doses from external exposure (sensitivity analyses – various parameters of the adjustment for alpha and neutron dose, males, residents)

| Cutpoint,  Gy/year | Model  parameters | Sensitivity analysis type | | |
| --- | --- | --- | --- | --- |
|  |  | Exclusion of the adjustment for liver absorbed alpha dose | The alternative adjustment for liver absorbed alpha dose ^a^ | Association with the weighted sum of liver absorbed gamma-ray + neutron dose (Gy) ^b^ |
| 0 (without cutpoint [16]) | ERR/Gy | **0.09 (+0.00, 0.19)** | 0.06 (–0.03, 0.18) | 0.09 (–0.01, 0.22) |
| 0.005 | ERR_L_/Gy | **–5.41 (–7.30, –3.17)** | **–5.99 (–7.85, –3.74)** | **–5.35 (–7.32, –2.98)** |
|  | ERR_H_/Gy | 0.08 (–0.00, 0.17) | 0.07 (–0.02, 0.19) | 0.05 (–0.04, 0.15) |
|  | *p* value ^c^ | **< 0.001** | **< 0.001** | **< 0.001** |
| 0.010 | ERR_L_/Gy | **–3.01 (–3.90, –1.97)** | **–3.25 (–4.14, –2.20)** | **–3.10 (–3.99, –2.06)** |
|  | ERR_H_/Gy | **0.09 (0.01, 0.18)** | **0.09 (+0.00, 0.21)** | 0.06 (–0.02, 0.17) |
|  | *p* value ^c^ | **< 0.001** | **< 0.001** | **< 0.001** |
| 0.015 | ERR_L_/Gy | **–2.03 (–2.65, –1.30)** | **–2.10 (–2.74, –1.34)** | **–2.13 (–2.74, –1.41)** |
|  | ERR_H_/Gy | **0.10 (0.02, 0.19)** | **0.10 (0.01, 0.22)** | 0.07 (–0.01, 0.18) |
|  | *p* value ^c^ | **< 0.001** | **< 0.001** | **< 0.001** |
| 0.020 | ERR_L_/Gy | **–1.27 (–1.78, –0.68)** | **–1.27 (–1.80, –0.63)** | **–1.40 (–1.89, –0.82)** |
|  | ERR_H_/Gy | **0.11 (0.03, 0.21)** | **0.12 (0.02, 0.25)** | **0.09 (+0.00, 0.21)** |
|  | *p* value ^c^ | **< 0.001** | **< 0.001** | **< 0.001** |
| 0.025 | ERR_L_/Gy | **–0.84 (–1.27, –0.34)** | **–0.81 (–1.28, –0.25)** | **–0.97 (–1.40, –0.48)** |
|  | ERR_H_/Gy | **0.11 (0.03, 0.21)** | **0.13 (0.02, 0.26)** | **0.10 (0.01, 0.22)** |
|  | *p* value ^c^ | **< 0.001** | **0.002** | **< 0.001** |
| 0.030 | ERR_L_/Gy | **–0.54 (–0.92, –0.10)** | –0.49 (–0.91, 0.02) | **–0.71 (–1.08, –0.27)** |
|  | ERR_H_/Gy | **0.12 (0.03, 0.22)** | **0.13 (0.02, 0.27)** | **0.11 (0.01, 0.24)** |
|  | *p* value ^c^ | **0.006** | **0.022** | **< 0.001** |
| 0.035 | ERR_L_/Gy | –0.27 (–0.62, 0.14) | –0.17 (–0.58, 0.32) | **–0.42 (–0.77, –0.01)** |
|  | ERR_H_/Gy | **0.11 (0.02, 0.22)** | **0.12 (0.01, 0.26)** | **0.11 (+0.00, 0.23)** |
|  | *p* value ^c^ | 0.073 | 0.249 | **0.019** |
| 0.040 | ERR_L_/Gy | –0.15 (–0.46, 0.22) | –0.03 (–0.40, 0.41) | –0.26 (–0.58, 0.11) |
|  | ERR_H_/Gy | **0.11 (0.02, 0.21)** | 0.11 (–0.00, 0.25) | 0.10 (–0.01, 0.23) |
|  | *p* value ^c^ | 0.179 | > 0.50 | 0.069 |
| 0.045 | ERR_L_/Gy | –0.06 (–0.34, 0.28) | 0.07 (–0.27, 0.48) | –0.18 (–0.47, 0.16) |
|  | ERR_H_/Gy | **0.10 (0.01, 0.21)** | 0.10 (–0.01, 0.24) | 0.10 (–0.01, 0.23) |
|  | *p* value ^c^ | 0.334 | > 0.50 | 0.127 |
| 0.050 | ERR_L_/Gy | –0.01 (–0.28, 0.31) | 0.13 (–0.19, 0.52) | –0.10 (–0.37, 0.22) |
|  | ERR_H_/Gy | **0.10 (0.01, 0.21)** | 0.09 (–0.02, 0.23) | 0.09 (–0.02, 0.22) |
|  | *p* value ^c^ | 0.489 | > 0.50 | 0.275 |
| *Notes:*  Numbers in bold indicate significant differences.  CI, confidence interval. ERR/Gy, excess relative risk per unit gray of gamma-ray dose. IHD, ischemic heart disease (ICD-9 codes: 410–414). n/a denotes that it was not possible to estimate the lower bound or upper of the confidence interval.  ^a^ Unmonitored for plutonium alpha activity workers divided into two subgroups: only workers of reactors and the rest of unmonitored workers.  ^b^ For all workers.  ^c^ Likelihood ratio test comparing the models with and without cutpoint. | | | | |

Table S21 Excess relative risk per Gy of IHD mortality in relation to 10-year lagged cumulative liver absorbed gamma-ray doses from external exposure (sensitivity analyses – various parameters of the adjustment for alpha and neutron dose, females, residents)

| Cutpoint,  Gy/year | Model  parameters | Sensitivity analysis type | | |
| --- | --- | --- | --- | --- |
|  |  | Exclusion of the adjustment for liver absorbed alpha dose | The alternative adjustment for liver absorbed alpha dose ^a^ | Association with the weighted sum of liver absorbed gamma-ray + neutron dose (Gy) ^b^ |
| 0 (without cutpoint [16]) | ERR/Gy | –0.03 (–0.16, 0.14) | 0.14 (–0.07, 0.45) | 0.16 (–0.07, 0.47) |
| 0.005 | ERR_L_/Gy | –2.02 (–6.44, 3.80) | –1.90 (–6.71, 4.67) | –2.25 (–6.93, 4.12) |
|  | ERR_H_/Gy | –0.03 (–0.16, 0.14) | 0.14 (–0.07, 0.45) | 0.13 (–0.07, 0.43) |
|  | *p* value ^c^ | 0.461 | 0.497 | 0.419 |
| 0.010 | ERR_L_/Gy | –1.65 (–3.46, 0.69) | –1.60 (–3.61, 1.10) | –1.67 (–3.63, 0.93) |
|  | ERR_H_/Gy | –0.03 (–0.16, 0.13) | 0.14 (–0.07, 0.44) | 0.14 (–0.07, 0.43) |
|  | *p* value ^c^ | 0.158 | 0.181 | 0.154 |
| 0.015 | ERR_L_/Gy | –0.94 (–2.27, 0.78) | –0.96 (–2.45, 1.04) | –1.22 (–2.62, 0.65) |
|  | ERR_H_/Gy | –0.02 (–0.15, 0.14) | 0.16 (–0.06, 0.47) | 0.16 (–0.06, 0.46) |
|  | *p* value ^c^ | 0.268 | 0.242 | 0.134 |
| 0.020 | ERR_L_/Gy | –0.81 (–1.86, 0.55) | –0.86 (–2.03, 0.72) | –0.98 (–2.08, 0.49) |
|  | ERR_H_/Gy | –0.02 (–0.15, 0.14) | 0.16 (–0.05, 0.47) | 0.16 (–0.05, 0.47) |
|  | *p* value ^c^ | 0.232 | 0.181 | 0.116 |
| 0.025 | ERR_L_/Gy | –0.79 (–1.66, 0.35) | –0.83 (–1.79, 0.48) | –0.89 (–1.79, 0.35) |
|  | ERR_H_/Gy | –0.02 (–0.15, 0.15) | 0.17 (–0.05, 0.48) | 0.16 (–0.05, 0.47) |
|  | *p* value ^c^ | 0.169 | 0.123 | 0.088 |
| 0.030 | ERR_L_/Gy | –0.45 (–1.28, 0.63) | –0.39 (–1.32, 0.88) | –0.50 (–1.37, 0.68) |
|  | ERR_H_/Gy | –0.02 (–0.15, 0.15) | 0.16 (–0.06, 0.48) | 0.16 (–0.06, 0.47) |
|  | *p* value ^c^ | 0.398 | 0.355 | 0.245 |
| 0.035 | ERR_L_/Gy | –0.44 (–1.19, 0.53) | –0.44 (–1.27, 0.69) | –0.48 (–1.27, 0.59) |
|  | ERR_H_/Gy | –0.02 (–0.15, 0.15) | 0.16 (–0.05, 0.48) | 0.16 (–0.05, 0.47) |
|  | *p* value ^c^ | 0.361 | 0.258 | 0.21 |
| 0.040 | ERR_L_/Gy | –0.34 (–1.06, 0.58) | –0.32 (–1.11, 0.76) | –0.38 (–1.13, 0.65) |
|  | ERR_H_/Gy | –0.02 (–0.15, 0.15) | 0.17 (–0.05, 0.49) | 0.17 (–0.05, 0.48) |
|  | *p* value ^c^ | 0.46 | 0.34 | 0.268 |
| 0.045 | ERR_L_/Gy | –0.16 (–0.87, 0.75) | –0.09 (–0.89, 1.00) | –0.19 (–0.95, 0.84) |
|  | ERR_H_/Gy | –0.02 (–0.16, 0.15) | 0.16 (–0.06, 0.48) | 0.16 (–0.06, 0.47) |
|  | *p* value ^c^ | > 0.50 | > 0.50 | 0.473 |
| 0.050 | ERR_L_/Gy | –0.18 (–0.84, 0.68) | –0.10 (–0.86, 0.93) | –0.18 (–0.90, 0.80) |
|  | ERR_H_/Gy | –0.02 (–0.16, 0.15) | 0.16 (–0.06, 0.48) | 0.16 (–0.06, 0.47) |
|  | *p* value ^c^ | > 0.50 | > 0.50 | 0.465 |
| *Notes:*  Numbers in bold indicate significant differences.  CI, confidence interval. ERR/Gy, excess relative risk per unit gray of gamma-ray dose. IHD, ischemic heart disease (ICD-9 codes: 410–414). n/a denotes that it was not possible to estimate the lower bound or upper of the confidence interval.  ^a^ Unmonitored for plutonium alpha activity workers divided into two subgroups: only workers of reactors and the rest of unmonitored workers.  ^b^ For all workers.  ^c^ Likelihood ratio test comparing the models with and without cutpoint. | | | | |

Table S22 Excess relative risk per Gy of IHD mortality in relation to 10-year lagged cumulative liver absorbed gamma-ray doses from external exposure (sensitivity analyses – dataset restricted and additional inclusion of the adjustment, males, residents)

| Cutpoint,  Gy/year | Model  parameters | Sensitivity analysis type | | |
| --- | --- | --- | --- | --- |
|  |  | Dataset restricted to workers employed longer than one year | Inclusion of the adjustment for period of hire | Inclusion of the adjustment for age at hire |
| 0 (without cutpoint [16]) | ERR/Gy | 0.10 (–0.02, 0.25) | 0.06 (–0.04, 0.18) | 0.07 (–0.04, 0.20) |
| 0.005 | ERR_L_/Gy | **–5.68 (–7.60, –3.36)** | **–5.19 (–7.24, –2.72)** | **–5.17 (–7.30, –2.57)** |
|  | ERR_H_/Gy | 0.05 (–0.04, 0.16) | 0.04 (–0.05, 0.16) | 0.05 (–0.05, 0.17) |
|  | *p* value ^a^ | **< 0.001** | **< 0.001** | **< 0.001** |
| 0.010 | ERR_L_/Gy | **–3.23 (–4.12, –2.18)** | **–3.18 (–4.08, –2.11)** | **–3.14 (–4.07, –2.02)** |
|  | ERR_H_/Gy | 0.07 (–0.02, 0.18) | 0.06 (–0.03, 0.17) | 0.07 (–0.03, 0.19) |
|  | *p* value ^a^ | **< 0.001** | **< 0.001** | **< 0.001** |
| 0.015 | ERR_L_/Gy | **–2.22 (–2.83, –1.49)** | **–2.22 (–2.84, –1.50)** | **–2.27 (–2.90, –1.53)** |
|  | ERR_H_/Gy | 0.08 (–0.01, 0.19) | 0.07 (–0.02, 0.18) | 0.07 (–0.02, 0.20) |
|  | *p* value ^a^ | **< 0.001** | **< 0.001** | **< 0.001** |
| 0.020 | ERR_L_/Gy | **–1.45 (–1.95, –0.87)** | **–1.52 (–2.00, –0.95)** | **–1.58 (–2.07, –1.00)** |
|  | ERR_H_/Gy | **0.11 (0.01, 0.22)** | 0.09 (–0.01, 0.21) | 0.10 (–0.00, 0.23) |
|  | *p* value ^a^ | **< 0.001** | **< 0.001** | **< 0.001** |
| 0.025 | ERR_L_/Gy | **–1.02 (–1.44, –0.52)** | **–1.14 (–1.54, –0.65)** | **–1.19 (–1.59, –0.70)** |
|  | ERR_H_/Gy | **0.12 (0.02, 0.24)** | **0.10 (+0.00, 0.23)** | **0.12 (0.01, 0.26)** |
|  | *p* value ^a^ | **< 0.001** | **< 0.001** | **< 0.001** |
| 0.030 | ERR_L_/Gy | **–0.70 (–1.08, –0.26)** | **–0.83 (–1.19, –0.40)** | **–0.90 (–1.26, –0.47)** |
|  | ERR_H_/Gy | **0.12 (0.02, 0.25)** | **0.11 (0.01, 0.25)** | **0.13 (0.02, 0.28)** |
|  | *p* value ^a^ | **0.001** | **< 0.001** | **< 0.001** |
| 0.035 | ERR_L_/Gy | –0.40 (–0.76, 0.02) | **–0.53 (–0.87, –0.13)** | **–0.62 (–0.96, –0.22)** |
|  | ERR_H_/Gy | **0.12 (0.01, 0.25)** | 0.11 (–0.00, 0.25) | **0.13 (0.01, 0.29)** |
|  | *p* value ^a^ | **0.024** | **0.005** | **< 0.001** |
| 0.040 | ERR_L_/Gy | –0.26 (–0.58, 0.12) | **–0.39 (–0.70, –0.02)** | **–0.48 (–0.78, –0.12)** |
|  | ERR_H_/Gy | **0.11 (+0.00, 0.25)** | 0.11 (–0.00, 0.25) | **0.14 (0.02, 0.29)** |
|  | *p* value ^a^ | 0.07 | **0.014** | **0.003** |
| 0.045 | ERR_L_/Gy | –0.15 (–0.44, 0.20) | –0.27 (–0.55, 0.07) | **–0.37 (–0.64, –0.04)** |
|  | ERR_H_/Gy | 0.10 (–0.01, 0.24) | 0.11 (–0.01, 0.25) | **0.14 (0.01, 0.30)** |
|  | *p* value ^a^ | 0.174 | **0.044** | **0.007** |
| 0.050 | ERR_L_/Gy | –0.08 (–0.36, 0.25) | –0.20 (–0.47, 0.12) | –0.29 (–0.54, 0.03) |
|  | ERR_H_/Gy | 0.10 (–0.01, 0.24) | 0.10 (–0.01, 0.25) | **0.14 (0.01, 0.30)** |
|  | *p* value ^a^ | 0.313 | 0.085 | **0.018** |
| *Notes:*  Numbers in bold indicate significant differences.  CI, confidence interval. ERR/Gy, excess relative risk per unit gray of gamma-ray dose. IHD, ischemic heart disease (ICD-9 codes: 410–414). n/a denotes that it was not possible to estimate the lower or upper bound of the confidence interval.  ^a^ Likelihood ratio test comparing the models with and without cutpoint. | | | | |

Table S23 Excess relative risk per Gy of IHD mortality in relation to 10-year lagged cumulative liver absorbed gamma-ray doses from external exposure (sensitivity analyses – dataset restricted and additional inclusion of the adjustment, females, residents)

| Cutpoint,  Gy/year | Model  parameters | Sensitivity analysis type | | |
| --- | --- | --- | --- | --- |
|  |  | Dataset restricted to workers employed for >1 year | Inclusion of the adjustment for period of hire | Inclusion of the adjustment for age at hire |
| 0 (without cutpoint [16]) | ERR/Gy | 0.20 (–0.06, 0.58) | 0.19 (–0.05, 0.56) | **0.43 (0.08, 0.96)** |
| 0.005 | ERR_L_/Gy | –2.22 (–6.93, 4.24) | –1.39 (–6.40, 5.48) | –1.54 (–6.78, 5.82) |
|  | ERR_H_/Gy | 0.10 (–0.10, 0.38) | 0.19 (–0.05, 0.55) | **0.42 (0.08, 0.95)** |
|  | *p* value ^a^ | 0.436 | > 0.50 | > 0.50 |
| 0.010 | ERR_L_/Gy | –1.88 (–3.80, 0.68) | –1.70 (–3.71, 0.99) | –1.57 (–3.76, 1.44) |
|  | ERR_H_/Gy | 0.10 (–0.09, 0.38) | 0.18 (–0.05, 0.54) | **0.41 (0.08, 0.93)** |
|  | *p* value ^a^ | 0.116 | 0.148 | 0.167 |
| 0.015 | ERR_L_/Gy | –1.24 (–2.63, 0.62) | –1.08 (–2.56, 0.91) | –0.90 (–2.56, 1.41) |
|  | ERR_H_/Gy | 0.12 (–0.08, 0.40) | 0.20 (–0.04, 0.57) | **0.44 (0.10, 0.97)** |
|  | *p* value ^a^ | 0.138 | 0.182 | 0.216 |
| 0.020 | ERR_L_/Gy | –1.07 (–2.15, 0.39) | –0.93 (–2.09, 0.67) | –0.81 (–2.13, 1.04) |
|  | ERR_H_/Gy | 0.12 (–0.08, 0.41) | 0.20 (–0.04, 0.57) | **0.45 (0.10, 0.99)** |
|  | *p* value ^a^ | 0.101 | 0.144 | 0.151 |
| 0.025 | ERR_L_/Gy | –1.01 (–1.89, 0.20) | –0.88 (–1.84, 0.46) | –0.64 (–1.76, 0.98) |
|  | ERR_H_/Gy | 0.13 (–0.08, 0.41) | 0.21 (–0.03, 0.57) | **0.46 (0.11, 1.00)** |
|  | *p* value ^a^ | 0.063 | 0.096 | 0.152 |
| 0.030 | ERR_L_/Gy | –0.57 (–1.43, 0.60) | –0.48 (–1.41, 0.82) | –0.13 (–1.25, 1.46) |
|  | ERR_H_/Gy | 0.12 (–0.08, 0.41) | 0.20 (–0.04, 0.57) | **0.45 (0.10, 1.00)** |
|  | *p* value ^a^ | 0.22 | 0.259 | 0.414 |
| 0.035 | ERR_L_/Gy | –0.60 (–1.36, 0.43) | –0.49 (–1.33, 0.68) | –0.20 (–1.20, 1.25) |
|  | ERR_H_/Gy | 0.12 (–0.08, 0.42) | 0.20 (–0.04, 0.57) | **0.46 (0.10, 1.01)** |
|  | *p* value ^a^ | 0.151 | 0.206 | 0.32 |
| 0.040 | ERR_L_/Gy | –0.46 (–1.19, 0.54) | –0.34 (–1.15, 0.80) | –0.01 (–1.00, 1.39) |
|  | ERR_H_/Gy | 0.13 (–0.08, 0.42) | 0.21 (–0.04, 0.58) | **0.46 (0.10, 1.02)** |
|  | *p* value ^a^ | 0.226 | 0.302 | 0.451 |
| 0.045 | ERR_L_/Gy | –0.25 (–0.99, 0.76) | –0.12 (–0.94, 1.04) | 0.30 (–0.72, 1.76) |
|  | ERR_H_/Gy | 0.12 (–0.09, 0.42) | 0.20 (–0.04, 0.58) | **0.44 (0.09, 1.00)** |
|  | *p* value ^a^ | 0.437 | > 0.50 | > 0.50 |
| 0.050 | ERR_L_/Gy | –0.26 (–0.96, 0.69) | –0.12 (–0.90, 0.97) | 0.26 (–0.70, 1.64) |
|  | ERR_H_/Gy | 0.12 (–0.09, 0.42) | 0.20 (–0.04, 0.58) | **0.44 (0.09, 0.99)** |
|  | *p* value ^a^ | 0.399 | > 0.50 | > 0.50 |
| *Notes:*  Numbers in bold indicate significant differences.  CI, confidence interval. ERR/Gy, excess relative risk per unit gray of gamma-ray dose. IHD, ischemic heart disease (ICD-9 codes: 410–414). n/a denotes that it was not possible to estimate the lower or upper bound of the confidence interval.  ^a^ Likelihood ratio test comparing the models with and without cutpoint. | | | | |

Table S24 Excess relative risk per Gy of IHD mortality in relation to 10-year lagged cumulative liver absorbed gamma-ray doses from external exposure (non-linear analysis, males, residents)

| Cutpoint,  Gy/year | Model  parameters | Models ^a^ | | | |
| --- | --- | --- | --- | --- | --- |
|  |  | (1) | (2) ^b^ | (3) ^b^ | (4) ^b^ |
| 0.005 | β_L1_ | **–5.46 (–7.41, –3.13)** | **–8.35 (–14.55, –2.14)** | **–5.46 (–7.66, –3.27)** | **–8.33 (–14.54, –2.12)** |
|  | β_H1_ | 0.04 (–0.04, 0.15) | 0.04 (–0.05, 0.13) | 0.03 (–0.16, 0.23) | 0.04 (–0.16, 0.23) |
|  | β_L2_ | – | 47.11 (–51.31, 145.50) | – | 46.90 (–51.45, 145.20) |
|  | β_H2_ | – | – | +0.00 (–0.07, 0.08) | +0.00 (–0.07, 0.08) |
|  | *p* value ^c^ | – | 0.343 | > 0.50 | > 0.50 |
| 0.010 | β_L1_ | **–3.11 (–4.01, –2.06)** | –2.67 (–5.78, 0.44) | **–3.13 (–4.11, –2.15)** | –2.69 (–5.82, 0.44) |
|  | β_H1_ | 0.06 (–0.02, 0.17) | 0.06 (–0.03, 0.16) | 0.09 (–0.10, 0.28) | 0.09 (–0.10, 0.29) |
|  | β_L2_ | – | –3.59 (–26.99, 19.81) | – | –3.56 (–27.09, 19.98) |
|  | β_H2_ | – | – | –0.01 (–0.09, 0.06) | –0.01 (–0.09, 0.06) |
|  | *p* value ^c^ | – | > 0.50 | > 0.50 | > 0.50 |
| 0.015 | β_L1_ | **–2.14 (–2.76, –1.42)** | **–2.31 (–4.48, –0.15)** | **–2.18 (–2.87, –1.49)** | **–2.37 (–4.56, –0.18)** |
|  | β_H1_ | 0.07 (–0.01, 0.18) | 0.07 (–0.02, 0.17) | 0.13 (–0.06, 0.33) | 0.13 (–0.06, 0.32) |
|  | β_L2_ | – | 0.97 (–10.42, 12.37) | – | 1.07 (–10.43, 12.56) |
|  | β_H2_ | – | – | –0.03 (–0.10, 0.05) | –0.03 (–0.10, 0.05) |
|  | *p* value ^c^ | – | > 0.50 | 0.485 | > 0.50 |
| 0.020 | β_L1_ | **–1.42 (–1.91, –0.84)** | **–2.56 (–4.19, –0.93)** | **–1.47 (–2.04, –0.91)** | **–2.63 (–4.28, –0.98)** |
|  | β_H1_ | **0.10 (+0.00, 0.21)** | 0.09 (–0.00, 0.19) | 0.18 (–0.02, 0.39) | 0.18 (–0.02, 0.37) |
|  | β_L2_ | – | 4.74 (–1.92, 11.41) | – | 4.82 (–1.90, 11.54) |
|  | β_H2_ | – | – | –0.04 (–0.11, 0.04) | –0.04 (–0.11, 0.04) |
|  | *p* value ^c^ | – | 0.164 | 0.309 | 0.225 |
| 0.025 | β_L1_ | **–1.00 (–1.42, –0.50)** | **–1.43 (–2.80, –0.06)** | **–1.06 (–1.53, –0.58)** | **–1.51 (–2.90, –0.12)** |
|  | β_H1_ | **0.11 (0.01, 0.23)** | **0.11 (+0.00, 0.21)** | **0.21 (+0.00, 0.43)** | **0.21 (+0.00, 0.42)** |
|  | β_L2_ | – | 1.42 (–2.91, 5.75) | – | 1.48 (–2.89, 5.85) |
|  | β_H2_ | – | – | –0.05 (–0.13, 0.03) | –0.05 (–0.12, 0.03) |
|  | *p* value ^c^ | – | > 0.50 | 0.231 | 0.39 |
| 0.030 | β_L1_ | **–0.69 (–1.07, –0.25)** | –1.16 (–2.35, 0.03) | **–0.75 (–1.18, –0.33)** | **–1.23 (–2.43, –0.04)** |
|  | β_H1_ | **0.11 (0.01, 0.24)** | **0.11 (+0.00, 0.22)** | **0.23 (0.01, 0.45)** | **0.22 (0.01, 0.44)** |
|  | β_L2_ | – | 1.29 (–1.85, 4.43) | – | 1.32 (–1.84, 4.48) |
|  | β_H2_ | – | – | –0.05 (–0.13, 0.03) | –0.05 (–0.13, 0.03) |
|  | *p* value ^c^ | – | 0.421 | 0.197 | 0.312 |
| 0.035 | β_L1_ | –0.40 (–0.75, 0.02) | –0.71 (–1.76, 0.34) | **–0.45 (–0.84, –0.06)** | –0.76 (–1.82, 0.30) |
|  | β_H1_ | **0.11 (+0.00, 0.23)** | 0.10 (–0.01, 0.21) | 0.22 (–0.01, 0.45) | 0.21 (–0.01, 0.44) |
|  | β_L2_ | – | 0.72 (–1.60, 3.05) | – | 0.72 (–1.62, 3.06) |
|  | β_H2_ | – | – | –0.05 (–0.13, 0.03) | –0.05 (–0.13, 0.03) |
|  | *p* value ^c^ | – | > 0.50 | 0.233 | 0.408 |
| 0.040 | β_L1_ | –0.26 (–0.58, 0.11) | –0.71 (–1.59, 0.16) | –0.31 (–0.66, 0.05) | –0.76 (–1.65, 0.12) |
|  | β_H1_ | 0.10 (–0.01, 0.23) | 0.10 (–0.01, 0.21) | 0.21 (–0.02, 0.45) | 0.21 (–0.02, 0.43) |
|  | β_L2_ | – | 0.90 (–0.78, 2.57) | – | 0.91 (–0.77, 2.59) |
|  | β_H2_ | – | – | –0.05 (–0.13, 0.04) | –0.05 (–0.13, 0.03) |
|  | *p* value ^c^ | – | 0.294 | 0.249 | 0.293 |
| 0.045 | β_L1_ | –0.15 (–0.44, 0.19) | –0.60 (–1.37, 0.16) | –0.20 (–0.52, 0.13) | –0.66 (–1.43, 0.12) |
|  | β_H1_ | 0.09 (–0.01, 0.23) | 0.09 (–0.02, 0.20) | 0.22 (–0.02, 0.45) | 0.21 (–0.02, 0.44) |
|  | β_L2_ | – | 0.77 (–0.50, 2.04) | – | 0.79 (–0.48, 2.06) |
|  | β_H2_ | – | – | –0.05 (–0.14, 0.03) | –0.05 (–0.13, 0.03) |
|  | *p* value ^c^ | – | 0.231 | 0.231 | 0.23 |
| 0.050 | β_L1_ | –0.09 (–0.36, 0.23) | –0.53 (–1.25, 0.18) | –0.13 (–0.43, 0.18) | –0.59 (–1.31, 0.12) |
|  | β_H1_ | 0.09 (–0.02, 0.22) | 0.09 (–0.02, 0.20) | 0.21 (–0.03, 0.45) | 0.21 (–0.02, 0.44) |
|  | β_L2_ | – | 0.71 (–0.37, 1.78) | – | 0.73 (–0.35, 1.81) |
|  | β_H2_ | – | – | –0.05 (–0.14, 0.03) | –0.05 (–0.14, 0.03) |
|  | *p* value ^c^ | – | 0.205 | 0.229 | 0.205 |
| *Notes:*  Numbers in bold indicate significant differences. The dataset for the analysis was stratified by sex, attained age, calendar period, smoking status, alcohol consumption, alpha dose.  CI, confidence interval. ERR/Gy, excess relative risk per unit gray of gamma-ray dose. IHD, ischemic heart disease (ICD-9 codes: 410–414). n/a denotes that it was not possible to estimate the lower or upper bound of the confidence interval.  ^a^ Equations of the models: (1) λ = λ_0_(1 + β_L1_D_L_ + β_H1_D_H_), (2) λ = λ_0_(1 + β_L1_D_L_+ β_L2_D_L_^2^ + β_Η1_D_H_), (3) λ = λ_0_(1 + β_L1_D_L_+ β_Η1_D_H_ + β_Η2_D_H_^2^),  (4) λ = λ_0_(1 + β_L1_D_L_ + β_L2_D_L_^2^ + β_Η1_D_H_ + β_Η2_D_H_^2^)  ^b^ Wald-type confidence interval.  ^c^ *p*-value denotes significant differences from a linear model. | | | | | |

Table S25 Excess relative risk per Gy of IHD mortality in relation to 10-year lagged cumulative liver absorbed gamma-ray doses from external exposure (non-linear analysis, females, residents)

| Cutpoint,  Gy | Model  parameters | Models ^a^ | | | |
| --- | --- | --- | --- | --- | --- |
|  |  | (1) | (2) ^b^ | (3) ^b^ | (4) ^b^ |
| 0.005 | β_L1_ | –2.33 (–6.98, 4.01) | –0.41 (–13.88, 13.06) | –2.22 (–7.48, 3.03) | 0.16 (–12.91, 13.22) |
|  | β_H1_ | 0.13 (–0.07, 0.44) | 0.14 (–0.12, 0.40) | –0.11 (–0.64, 0.42) | –0.12 (–0.66, 0.42) |
|  | β_L2_ | – | –35.03 (–253.30, 183.30) | – | –43.62 (–254.30, 167.00) |
|  | β_H2_ | – | – | 0.14 (–0.14, 0.41) | 0.14 (–0.14, 0.42) |
|  | *p* value ^c^ | – | > 0.50 | 0.313 | > 0.50 |
| 0.010 | β_L1_ | –1.86 (–3.77, 0.69) | 2.33 (–4.04, 8.70) | –1.73 (–3.84, 0.39) | 2.57 (–3.69, 8.83) |
|  | β_H1_ | 0.14 (–0.07, 0.44) | 0.15 (–0.11, 0.41) | –0.08 (–0.60, 0.45) | –0.10 (–0.65, 0.45) |
|  | β_L2_ | – | –36.25 (–82.47, 9.97) | – | –37.07 (–82.33, 8.18) |
|  | β_H2_ | – | – | 0.12 (–0.16, 0.40) | 0.14 (–0.15, 0.43) |
|  | *p* value ^c^ | – | 0.197 | 0.369 | 0.271 |
| 0.015 | β_L1_ | –1.20 (–2.60, 0.65) | 1.81 (–1.59, 5.22) | –1.09 (–2.65, 0.47) | 1.80 (–1.57, 5.17) |
|  | β_H1_ | 0.16 (–0.05, 0.46) | 0.15 (–0.12, 0.42) | –0.04 (–0.58, 0.51) | –0.09 (–0.65, 0.47) |
|  | β_L2_ | – | **–18.26 (–30.33, –6.19)** | – | **–17.24 (–29.67, –4.81)** |
|  | β_H2_ | – | – | 0.11 (–0.18, 0.40) | 0.12 (–0.17, 0.42) |
|  | *p* value ^c^ | – | 0.185 | 0.427 | 0.32 |
| 0.020 | β_L1_ | –1.00 (–2.10, 0.48) | 0.83 (–3.00, 4.66) | –0.91 (–2.15, 0.34) | 0.85 (–2.93, 4.64) |
|  | β_H1_ | 0.16 (–0.05, 0.47) | 0.18 (–0.10, 0.46) | –0.02 (–0.56, 0.53) | –0.00 (–0.58, 0.57) |
|  | β_L2_ | – | –8.60 (–25.27, 8.06) | – | –8.32 (–24.80, 8.17) |
|  | β_H2_ | – | – | 0.10 (–0.19, 0.40) | 0.10 (–0.21, 0.41) |
|  | *p* value ^c^ | – | 0.301 | 0.471 | 0.462 |
| 0.025 | β_L1_ | –0.92 (–1.83, 0.32) | 0.27 (–2.72, 3.25) | –0.85 (–1.89, 0.19) | 0.30 (–2.66, 3.27) |
|  | β_H1_ | 0.17 (–0.05, 0.48) | 0.18 (–0.10, 0.45) | –0.01 (–0.56, 0.53) | –0.01 (–0.57, 0.56) |
|  | β_L2_ | – | –4.57 (–15.28, 6.14) | – | –4.45 (–15.11, 6.20) |
|  | β_H2_ | – | – | 0.10 (–0.19, 0.40) | 0.11 (–0.21, 0.42) |
|  | *p* value ^c^ | – | 0.383 | 0.47 | > 0.50 |
| 0.030 | β_L1_ | –0.47 (–1.36, 0.73) | 0.08 (–2.81, 2.97) | –0.40 (–1.42, 0.61) | 0.11 (–2.74, 2.96) |
|  | β_H1_ | 0.16 (–0.06, 0.47) | 0.17 (–0.11, 0.44) | –0.09 (–0.64, 0.47) | –0.09 (–0.65, 0.48) |
|  | β_L2_ | – | –1.95 (–11.71, 7.82) | – | –1.81 (–11.41, 7.80) |
|  | β_H2_ | – | – | 0.14 (–0.17, 0.45) | 0.14 (–0.17, 0.46) |
|  | *p* value ^c^ | – | > 0.50 | 0.333 | > 0.50 |
| 0.035 | β_L1_ | –0.50 (–1.29, 0.57) | 0.12 (–2.51, 2.75) | –0.44 (–1.35, 0.47) | 0.15 (–2.43, 2.74) |
|  | β_H1_ | 0.17 (–0.05, 0.48) | 0.17 (–0.10, 0.45) | –0.07 (–0.63, 0.49) | –0.07 (–0.64, 0.51) |
|  | β_L2_ | – | –1.96 (–9.95, 6.03) | – | –1.88 (–9.71, 5.96) |
|  | β_H2_ | – | – | 0.13 (–0.18, 0.45) | 0.14 (–0.18, 0.46) |
|  | *p* value ^c^ | – | > 0.50 | 0.368 | > 0.50 |
| 0.040 | β_L1_ | –0.37 (–1.13, 0.66) | 0.06 (–2.39, 2.51) | –0.31 (–1.19, 0.57) | 0.10 (–2.31, 2.51) |
|  | β_H1_ | 0.17 (–0.05, 0.48) | 0.17 (–0.11, 0.45) | –0.08 (–0.65, 0.50) | –0.08 (–0.66, 0.51) |
|  | β_L2_ | – | –1.28 (–8.19, 5.63) | – | –1.24 (–7.99, 5.52) |
|  | β_H2_ | – | – | 0.14 (–0.18, 0.46) | 0.14 (–0.18, 0.47) |
|  | *p* value ^c^ | – | > 0.50 | 0.352 | > 0.50 |
| 0.045 | β_L1_ | –0.14 (–0.91, 0.89) | 0.10 (–2.31, 2.51) | –0.07 (–0.96, 0.82) | 0.17 (–2.20, 2.54) |
|  | β_H1_ | 0.16 (–0.06, 0.48) | 0.16 (–0.12, 0.44) | –0.13 (–0.72, 0.45) | –0.14 (–0.73, 0.45) |
|  | β_L2_ | – | –0.71 (–7.20, 5.78) | – | –0.70 (–7.04, 5.63) |
|  | β_H2_ | – | – | 0.17 (–0.16, 0.50) | 0.17 (–0.16, 0.51) |
|  | *p* value ^c^ | – | > 0.50 | 0.275 | > 0.50 |
| 0.050 | β_L1_ | –0.15 (–0.88, 0.83) | –0.00 (–2.24, 2.23) | –0.08 (–0.93, 0.77) | 0.05 (–2.15, 2.26) |
|  | β_H1_ | 0.16 (–0.06, 0.48) | 0.16 (–0.12, 0.44) | –0.13 (–0.72, 0.46) | –0.13 (–0.72, 0.46) |
|  | β_L2_ | – | –0.40 (–6.06, 5.26) | – | –0.37 (–5.92, 5.18) |
|  | β_H2_ | – | – | 0.17 (–0.16, 0.50) | 0.17 (–0.17, 0.51) |
|  | *p* value ^c^ | – | > 0.50 | 0.286 | > 0.50 |
| *Notes:*  Numbers in bold indicate significant differences. The dataset for the analysis was stratified by sex, attained age, calendar period, smoking status, alcohol consumption, alpha dose.  CI, confidence interval. ERR/Gy, excess relative risk per unit gray of gamma-ray dose. IHD, ischemic heart disease (ICD-9 codes: 410–414). n/a denotes that it was not possible to estimate the lower or upper bound of the confidence interval.  ^a^ Equations of the models: (1) λ = λ_0_(1 + β_L1_D_L_ + β_H1_D_H_), (2) λ = λ_0_(1 + β_L1_D_L_+ β_L2_D_L_^2^ + β_Η1_D_H_), (3) λ = λ_0_(1 + β_L1_D_L_+ β_Η1_D_H_ + β_Η2_D_H_^2^),  (4) λ = λ_0_(1 + β_L1_D_L_ + β_L2_D_L_^2^ + β_Η1_D_H_ + β_Η2_D_H_^2^)  ^b^ Wald-type confidence interval.  ^c^ *p*-value denotes significant differences from a linear model. | | | | | |
